# Functional and Structural Responses of Arctic and Alpine Soil Prokaryotic and Fungal Communities Under Freeze-Thaw Cycles of Different Frequencies

**DOI:** 10.3389/fmicb.2020.00982

**Published:** 2020-05-25

**Authors:** Carla Perez-Mon, Beat Frey, Aline Frossard

**Affiliations:** Forest Soils and Biogeochemistry, Swiss Federal Institute for Forest, Snow and Landscape Research WSL, Birmensdorf, Switzerland

**Keywords:** alpine, arctic, freeze-thaw cycles, bacteria, fungi, climate change, global warming, snow scarcity

## Abstract

Ongoing climate change involves increasing snow scarcity, which results in more frequent freeze-thaw cycles (FTCs) in polar and alpine soils. Although repeated FTCs have been shown to alter the structure and functions of soil microbial communities, a thorough understanding on the influence of FTCs frequency on polar and especially alpine soil microbiomes is still elusive. Here, we investigated the impact of repeated weekly vs. daily FTC frequencies on the structure and functions of prokaryotic and fungal communities from north- and south-exposed soils from two mountain ridges, one in the Arctic and one in the High-Alps. FTCs affected prokaryotic communities more strongly than fungal communities, where mainly cold-tolerant and opportunistic fungi (e.g., *Mrakia*, *Mortierella*) were responsive. Prokaryotic communities were more affected by weekly FTCs than by daily FTCs. Daily FTCs favored fast-growing bacteria (e.g., *Arthrobacter*), while oligotrophic and largely uncultured taxa (e.g., Verrucomicrobia) benefited from weekly FTCs. FTCs negatively affected microbial respiration but had minor impacts on C-, N- and P-acquiring enzymatic activities. Plausible pre-adaptation of the microbial communities to naturally occurring frequent FTCs at their site of origin did not show a clear influence on the microbial responses to the tested FTCs. Altogether, our study provides an integrative overview on potential structural and functional changes of soil microbial communities in polar and alpine regions in response to the projected increase in FTCs; therefore advancing our understanding on the impact of climate change in these rapidly changing ecosystems.

## Introduction

Climate change scenarios predict an increase in air temperatures worldwide of up to 3.7°C by 2100, along with greater variation in air temperature and precipitation (IPCC, 2014). These climatological changes might have important consequences on soil ecosystems, where fluctuations in soil temperatures and moisture might alter microbial diversity (Balser et al., 2010), microbially mediated ecological functions (Steinweg et al., 2013; Karhu et al., 2014) and, consequently, the above-ground growth of vegetation (Henry, 2013; Classen et al., 2015). Soil temperature oscillations around 0°C cause freeze-thaw cycles (FTCs), which are common in polar and alpine regions, especially during late fall and early spring when the absence of snow means a closer coupling between air and soil temperatures (Henry, 2013). As winters are becoming warmer and temperature oscillations and precipitation are becoming more erratic, the frequency of FTCs is predicted to increase, especially in polar and alpine regions, where the snow is being extensively replaced by rain (Henry, 2013; IPCC, 2014). Snow-covered soils remain at subzero temperatures for long periods, and they thaw gradually as air temperatures increase and snow melts (Freppaz et al., 2007). Snow-free soils, on the contrary, respond to hourly air temperature variations and undergo daily FTCs when night temperatures fall below 0°C (Freppaz et al., 2007).

Polar and alpine regions are both dominated by subzero temperatures, and they have landscape features in common, such as snow, permafrost and glaciers (Margesin, 2009; Donhauser and Frey, 2018). However, alpine regions have more precipitation and UV radiation, as well as larger seasonal and diurnal fluctuations in incoming solar radiation and temperature, than polar regions (Margesin, 2009; Donhauser and Frey, 2018). In addition, alpine regions exhibit strong altitudinal gradients, resulting in marked climatological heterogeneity at the local scale (Margesin, 2009; Donhauser and Frey, 2018). Soils from the south-exposed slopes of mountains are warmer and experience more temperature variations and FTCs than soils from the north-exposed slopes, where snow is present for longer periods (Donhauser and Frey, 2018).

In polar and alpine soils, FTCs have been shown to alter the structure of microbial communities (Eriksson et al., 2001; Larsen et al., 2002; Yergeau and Kowalchuk, 2008; Mannisto et al., 2009; Han et al., 2018; Ren et al., 2018), the patterns of microbial substrate utilization (Schimel and Mikan, 2005; Han et al., 2018), and soil ecosystem processes such as C and N mineralization (Larsen et al., 2002; Schimel and Mikan, 2005; Stres et al., 2010; Han et al., 2018). Freezing decreases soil water availability, increases osmotic pressure and causes severe physical damages to the microbial cells (Mazur, 1984; Panoff et al., 1998). This leads to the death of vulnerable microorganisms and, thereupon, to changes in the biomass and composition of the microbial communities (Eriksson et al., 2001; Yergeau and Kowalchuk, 2008; Mannisto et al., 2009; Han et al., 2018; Ren et al., 2018). Furthermore, the alternation between freezing and thawing causes the breaking of soil aggregates (Feng et al., 2007; Wang et al., 2012), which results in the release of labile carbon and nutrient sources in the soil (Feng et al., 2007). The preferential consumption of these newly available carbon and nutrients by survivor microorganisms to FTCs often translates to metabolic shifts at the community level, from the degradation of complex organic matter to the utilization of simpler carbon sources (Schimel and Mikan, 2005; Stres et al., 2010; Han et al., 2018). Likewise, FTCs might lead to decreases in the rates of soil respiration and N mineralization, likely because of the decrease in microbial biomass (Larsen et al., 2002; Stres et al., 2010) or alterations in microbial activities (Schimel and Mikan, 2005; Han et al., 2018).

Studies in polar regions and the Himalayas suggest that soil-related factors influence the resistance and resilience (tolerance) of microbial communities to FTCs (Bolter et al., 2005; Schimel and Mikan, 2005; Stres et al., 2010). A main influencing factor could be the FTC legacy of the soil (Yergeau and Kowalchuk, 2008; Stres et al., 2010). Soils subjected to frequent FTCs might harbor microbial communities better adapted to these variations in temperature and therefore more tolerant to FTCs (Yergeau and Kowalchuk, 2008; Stres et al., 2010). As alpine soils undergo greater temperature variation than polar soils, distinct responses of microbial communities to an increase in FTC frequency can be expected between alpine and polar soils. Moreover, differences might already be detectable at the local scale, between north- and south-exposed soils, in alpine areas.

An increase in FTC frequency, such as more daily FTCs, could mean more frequent alterations in the polar and alpine soil ecosystems, resulting in considerable changes in soil microbial community structures and functions. The magnitude of the impact of this increase in FTC frequency could vary between the different microbial taxa. For instance, fungal communities have been found to be more tolerant to repeated FTCs than prokaryotes (Bacteria and Archaea) (Sharma et al., 2006; Han et al., 2018; Schostag et al., 2019). Further, the genomic study of Schostag et al. (2019) suggests that the ability of specific bacterial taxa to adjust to FTC temperature variations and associated changes in the soil environment might depend on their physiological traits (e.g., growth rate and metabolic versatility). However, detailed knowledge about the influence of repeated FTCs on polar and especially alpine soil microbiomes is still scarce. Moreover, fungal communities are often overlooked in FTC studies and we are not aware of interregional investigations that evaluate the effect of increasing FTC frequency on both polar and alpine soils. In the rapidly changing polar and alpine ecosystems, a comprehensive understanding on the changes in microbial composition in response to FTCs and their relation to potential alterations on soil microbially mediated processes is much needed.

We conducted a microcosm experiment with north- (N) and south-exposed (S) top soils from two mountain ridges, one in the Swedish Arctic and one in the High-Alps in Switzerland (soils of four different origin: Arctic-N and -S, Alps-N and -S) to evaluate the effects of repeated FTC of differing frequencies on both prokaryotic and fungal communities. The soils were incubated for 28 days under daily FTCs (D-FTC, alternations of 12 h at +5°C and 12 h at −5°C) and weekly FTCs (W-FTC, alternations of 7 days at +5°C and 7 days at −5°C), including controls (+5°C and −5°C) (Supplementary Figure S1). We hypothesized that: (1) repeated short and frequent FTCs cause larger alterations in the structure of soil microbial communities, (2) microbial compositional shifts are coupled to alterations in microbial metabolic functions, and (3) microbial communities from soils with a legacy of more frequent FTCs exhibit a higher tolerance to repeated shorter and more frequent FTCs.

## Materials and Methods

### Site Description

Study sites were located on north- and south-exposed slopes near the summits of *Latnjachorru* in the Arctic (N 68°21.31500′ E 018°31.16600′, 1300 m a.s.l.) and *Muot de Barba Peider* in the Alps (N 46° 29.78040′ E 009°55.88700′, 2979 m a.s.l.). *Latnjachorru* is in Abisko, in the northernmost part of Sweden. Soil temperature measurements made on site at a depth of 5 cm for 1 year (iButtons; Maxim Integrated, San Jose, CA, United States) showed a mean annual temperature (MAT) of −3°C (range −15°C to 20°C) for the north-exposed slope and −1°C (range −12°C to 17°C) for the south-exposed slope. Mean annual precipitation (MAP) in the region is 838 mm, most of which falls as snow from September to June (based on daily soil surface temperatures measured from 2006 to 2015 at Lantjajaure Field Station, 981 m a.s.l.). The bedrock is mainly composed of Cambro-Silurion mica-garnet schists (Beylich et al., 2004). Permafrost in the area is sporadic and may be found at 80 m depth at 1200 m a.s.l. (Beylich et al., 2006).

*Muot de Barba Peider* is situated in the upper Engadine valley, in the eastern part of Switzerland. Soil temperature measurements made at a depth of 5 cm at the summit during two consecutive years showed a MAT of −1.5°C (range −13°C to 23°C) for the north-exposed slope and 2°C (range −5°C to 25°C) for the south-exposed slope. MAP in the region is 1500 mm (Frey et al., 2016). Snow establishes from early in October to late in November and lasts until the end of April or as late as the end of June (Beniston et al., 2003; Rodder and Kneisel, 2012). The bedrock consists of gneiss from the upper Austroalpine Languard nappe, and permafrost is found at a depth of 1.5–2 m (Zenklusen Mutter et al., 2010).

Soil temperature measurements indicated larger temperature variations at the alpine site than at the arctic site (Supplementary Figure S2). Furthermore, oscillation of hourly temperatures around 0°C suggested a mean value of 10 FTCs for the north- and south-slopes at the arctic sites, and mean values of 12 and 25 FTCs at the north- and south-slopes at the alpine site, respectively (1 year measurements). Vegetation is scarce at both sites and is dominated by non-woody tundral species. The arctic site is populated by grass members of the genera *Agrostis*, *Calamagrostris*, *Festuca*, *Poa*, and *Trisetum*, as well as bryophytes and lichens, such as *Cladonia* spp. and *Dicranum* spp. (Jagerbrand and Alatalo, 2015). Vegetation in the alpine site is scarcer than in the arctic site, with some occurrences of *Poa*, *Cerastium*, and *Jacobea* spp. (Frey et al., 2016).

### Sample Collection and Experimental Set-Up

Soil samples were collected in August 2017 at a depth of 5 cm in the north- and south-exposed slopes of the arctic and alpine mountains. In each slope, subsamples were collected with shovels from five locations separated by 3–5 m. In the field, the five subsamples were pooled, mixed and sieved (4 mm mesh size). All steps were performed using sterilized materials. Four soil pooled samples were obtained: north-exposed arctic soil (Arctic-N), south-exposed arctic soil (Arctic-S), north-exposed alpine soil (Alps-N), and south-exposed alpine soil (Alps-S). Soil samples were stored at 5°C for 1 month. For each soil sample, 16 microcosms were prepared by adding 50 g of fresh soil to 150 ml Erlenmeyer flasks, which were then covered with gas-permeable lids made from cotton wool to allow for aeration (Luláková et al., 2019). The microcosms were divided into four groups (each with four replicates), which were subsequently incubated under different temperature regimes (Supplementary Figure S1): (1) daily FTCs (D-FTC) with alternations of 12 h at +5°C and 12 h at −5°C, (2) weekly FTCs (W-FTC) with alternations of 7 days at +5°C and 7 days at −5°C, (3) controls with constant +5°C (+5°C), and (4) controls with constant −5°C (−5°C). The incubations lasted 28 days. Water content (WC) of the soil’s microcosms was determined at the start of the experiment by weighing soil samples before and after drying at 105°C. WC values for the soils of different origin corresponded in average to 50% of water holding capacity (WHC) for the north-exposed and the south-exposed arctic soils, 32% for the north-exposed alpine soils and 40% for the south-exposed alpine soils (Supplementary Table S1). WC were maintained constant during the incubation by replacing the evaporated water with sterilized milliQ water. The experimental temperature oscillations (between +5°C and −5°C) were chosen in relation to the observed natural range of top soil temperature variations occurring at both the arctic and alpine sites during the months of September/October and April/May, when FTCs were observed (Supplementary Figure S2). Monitoring of temperatures in dummy soils via iButtons during the tested FTCs in the incubators indicated that soil temperatures changed from +5 to −5°C and *vice versa* in about 2 h or less (freezing rates of −0.07 ± 0.003°C min^–1^ and thawing rates of 0.09 ± 0.005°C min^–1^, *n* = 2).

### Soil Physico-Chemical Properties

Soil texture was analyzed by the hydrometer method (Gee and Bauder, 1986). Water-holding capacity (WHC) was measured as the differences in weight between drained and dried soils as described in Frossard et al. (2017). Soil organic matter (OM) content was determined by the weight loss-on-ignition method (Davies, 1974). pH was measured in 0.01 M CaCl_2_ soil slurries (2:1 v/w). Total carbon (C) and nitrogen (N) were measured for dried (65°C) and fine-grained soil samples, using an elemental analyzer (NC-2500; CE Instruments, Wigan, United Kingdom). To measure labile C and nutrients, soil extracts were prepared in milliQ water (water:soil 10:1 v/w, shaken over night at room temperature) and filtered through DF 5895-150 ashless paper (Albert LabScience, Dassel, Germany). Labile organic C and total N were measured with a TOC/DTN analyzer (Sakalar Analytical B.V, Breda, Netherlands) after acid digestion with 3 M HCl to remove carbonates. Ammonium was measured photometrically with an FIAS 300 flow injection system (Perkin-Elmer, Waltham, MA, United States).

### Basal Respiration Rates

Soil basal respiration was measured once per week during the incubation period in the D-FTC, W-FTC and control +5°C microcosms. Measurements were performed at +5°C 2 h after the onset of the thawing phase of the D-FTC samples (four measurements) and at the end of the thawing phase of the W-FTC microcosms (two measurements). The respiration measurements were performed 7 days after the start of the incubation, to avoid the potential artifacts on microbial activities created by the soil manipulation during experiment set-up. The microcosms were gas-tight sealed, and CO_2_ concentrations accumulating in the flasks were measured at fixed intervals over a total time span of 8 h using an infrared absorption CO_2_ analyzer (EGM-4 Environmental Gas Monitor; PP systems, Amesbury, MA, United States) (Luláková et al., 2019). CO_2_ rates were calculated as the slopes of fitted linear regressions of CO_2_ concentrations over time. Regressions with *R*^2^ < 0.8 were discarded. Before and after each respiration measuring period, microcosms we aeriated and covered with the gas-permeable lids.

### Enzymatic Activity Rates

The potential activity of six extracellular enzymes, chosen based on their metabolic functions, was assessed: (1) β-glucosidases (BG), involved in the degradation of cellulose (C-acquiring enzyme); (2) β-xylosidases (XYL), involved in the degradation of hemicellulose (C-acquiring enzyme); (3) N-acetyl-glucosaminidases (NAG), involved in the degradation of β-1,4 glucosamines (C- and N-acquiring enzyme); (4) Leucine aminopeptidases (LAP), involved in the degradation of peptides (N-acquiring enzyme); (5) Acid phosphatases (AP), involved in the degradation of phosphomonoesters (P-acquiring enzyme); and (6) Phenol peroxidases (PP), involved in polyphenol oxidation (lignin-degrading enzyme) (Sinsabaugh et al., 2008). Activity measurements were performed on soil slurries (buffer:soil 3:1 v/w, acetate buffer, pH = 4, autoclaved) as previously described (Frossard et al., 2012). The activity of the first five enzymes (BG, XYL, NAG, LAP, and AP) were measured by fluorescence, using substrate analogs attached to fluorescent molecules 4-methylumbelliferone (MUB) or 7-amino-4-methylcoumarin (AMC). Phenol peroxidase activity (PP) was measured by colorimetry through the oxidative reaction of the substrate analog 3-3,4-dihydroxyphenylalanine (L-DOPA) in the presence of H_2_O_2_. The substrate analogs were added to final concentrations of 40 μM, after determining that these concentrations saturated potential enzymatic activities in the tested soils. The extracellular enzymatic activities were measured at the end of the 28-day incubation period. The assays were performed at +5°C, and controls were included in the assays to correct for variations in fluorescence and absorbance backgrounds of the substrate solutions and the slurries.

### Microbial Abundance

Bacterial and fungal abundances were determined by quantitative PCR (qPCR) on a 7500 Fast Real-Time PCR System (Thermo Fisher Scientific, Waltham, MA, United States). qPCR reactions were prepared using the universal primers pairs 27F/519R amplifying the V1-V3 region of the 16S rRNA gene (prokaryotes) and ITS3/ITS4 amplifying the internal transcribed spacer region 2 (ITS2) of the eukaryotic ribosomal operon (fungi). qPCR programs were run as described by Hartmann et al. (2014).

### DNA Extraction and High-Throughput Sequencing

Total soil DNA was extracted with the DNeasy PowerSoil Kit (Qiagen, Hilden, Germany) and DNA concentrations were quantified with the PicoGreen dsDNA quantification kit (Thermo Fisher) according to the manufacturer’s instructions. The 16S rRNA_*V*__3__–V__4_ genomic region (prokaryotes) and the ITS2 region (fungi) were amplified by PCR using primer pairs 341F/806R and ITS3/ITS4, respectively, and 10 ng of extracted DNA, as previously described (Frey et al., 2016). A first amplification was performed in triplicate, and the pooled prokaryotic and eukaryotic amplicons were sent to the Génome Québec Innovation Centre at McGill University (Montreal, QC, Canada), where they were purified, quantified, barcoded and paired-end sequenced using the Illumina MiSeq v3 platform (Illumina Inc., San Diego, CA, United States).

Prokaryotic and eukaryotic raw sequences were quality filtered, chimeras were removed, and high-quality reads were clustered into operational taxonomic units (OTUs) based on 97% identity threshold as described previously, using a customized pipeline largely based on UPARSE (Edgar, 2013; Edgar and Flyvbjerg, 2015) and implemented in USEARCH v.9.2 (Edgar, 2010). Singletons were removed. Taxonomic classification of the OTUs was performed by querying centroid sequences against reference databases using the naive Bayesian classifier (Wang et al., 2007), implemented in MOTHUR (Schloss et al., 2009), with a minimum bootstrap support of 60%. Prokaryotic sequences were queried against the SILVA database v.132 (Quast et al., 2013). Eukaryotic sequences were first curated using a custom-made ITS2 database retrieved from NCBI GenBank, and sequences assigned to fungi were classified to finer taxonomic levels using the UNITE database v.8.0 (Abarenkov et al., 2010).

### Data Analyses

Statistical analyses were completed, and graphs were generated using the open-source software R [version 3.6.0, R Core Team (2017) with the *ggplot2* package, Wickham (2016)]. A significance level of 0.05 was considered for all analyses. Overall effects of treatment (D-FTC, W-FTC, +5°C and −5°C), soil origin (Arctic-N, Arctic-S, Alps-N, and Alps-S) and their interactions on the soil physico-chemical properties, basal respiration rates and enzymatic activity rates were tested with two-way ANOVAs. Normality of residuals and homoscedasticity of the response variables were checked, and transformations (normalization or log10) were applied when ANOVA conditions were not met. The effects of the FTC treatments (D-FTC and W-FTC) on the variables *basal respiration rate* and *enzymatic activity rate* were further tested with one-way ANOVAs and Tukey HSD *post hoc* tests for each of the four different soils, separately. To ease the visualization and comparison of trends among the soils of different origin, respiration and enzymatic activity rates were transformed into relative values by dividing the rates by the mean values of the +5°C controls within the four soil subsets. The relative rates of the enzymatic activities were further log_2_ transformed.

The α-diversity parameters richness, Pielou’s evenness and Shannon diversity index were calculated on prokaryotic and fungal OTUs rarefied to the minimum number of reads (25936 for prokaryotes and 14324 for fungi), using an iterative approach [100 repetitions; *EcolUtils* package in R, Salazar (2019)]. Differences in microbial β-diversity were assessed by computing Bray-Curtis dissimilarity matrices based on the relative abundance of the prokaryotic or fungi OTUs and visualized with Principal Coordinate Analyses (PCoAs) [*vegan* package in R, Oksanen et al. (2019) and *cmdscale* function]. The statistical significance of observed differences was assessed with permutational analyses of variance [PERMANOVA, 10^5^ permutations, Anderson (2001)]. Multivariate homogeneity of group dispersions was checked prior to the PERMANOVAs to ensure that detected significant differences were associated with the tested factors and not with differences in the within-group variabilities (Anderson and Walsh, 2013). Where dispersion among groups was significant, statistics were performed using Chord distance instead of Bray-Curtis matrices (Legendre and Gallagher, 2001).

Indicator analyses were performed on the OTU relative abundances using the multipatt function implemented in the *indicspecies* package in R (v1.7.9; Cáceres and Legendre, 2009). Fungal OTUs indicators were functionally annotated with FungGuild v1.1 (Nguyen et al., 2016).

## Results

### Soil Physico-Chemical Properties

Soil properties, determined at the beginning of the incubation experiment for the four soils of different origin, showed marked differences across the soils (Supplementary Figure S3). At the end of the 28 days of incubation, soil origin showed a stronger influence than FTC treatments on all measured soil physico-chemical and microbial parameters (two-way ANOVAs, Table 1). All soils were acidic (pH 4.0–4.6) and had a high sand content (67–90%) (Supplementary Table S1). WHC, OM, carbon (C), and nitrogen (N) were higher (≥2-fold) in the arctic soils than in the alpine soils (Supplementary Table S1). Similar trends were observed between the south-exposed soils and the north-exposed soils for both arctic and alpine sites, where soils from the south-exposed slope consistently showed higher values for WHC, OM, C, and N (Supplementary Table S1). The FTC treatments had a significant effect on labile N (two-way ANOVAs, *F* = 16.87, *p* < 0.001), labile ammonium (*F* = 3.71, *p* < 0.05), and labile organic carbon (*F* = 3.22, *p* < 0.05) concentrations (Table 1). However, effects were consistent across soils only partially for labile N, which was four times higher in the W-FTC treatment than in the +5°C control for both north- and south-exposed alpine soils (Supplementary Tables S2, S3).

**TABLE 1:** Effects of treatment, soil origin and their interaction on soil properties, microbial activities, microbial abundance and prokaryotic and fungal α-diversity.

	**Variables**	**Treatment**	**Soil origin**	**Treatment × Soil origin**
		***F*_3_,_48_**	***F*_3_,_48_**	***F*_9_,_48_**
Soil properties^1^	pH	2.79	226.50***	1.11
	OM	2.29	454.78***	0.99
	labile OC	3.22*	50.10***	3.43**
	labile N	16.87***	368.69***	7.53***
	Ammonium	3.71*	356.26***	4.63***
Microbial activities^2^	Respiration^3^	87.64***	3.82*	3.82**
	BG	1.03	1.94	0.65
	XYL	0.27	1.02	0.45
	AP	0.25	4.72**	0.76
	NAG	0.51	2.50	0.79
	LAP	0.97	8.22***	3.65**
	PP	0.40	0.17	0.99
Microbial abundance	DNA content	1.42	97.13***	1.33
	Prokaryotic abundance^4^	1.58	1105.43***	1.64
	Fungal abundance^5^	2.11	6.35**	1.10
Prokaryotic α-diversity	Richness	6.61***	96.37***	3.85***
	Evenness	5.29**	238.80***	8.76***
	Shannon	9.62***	297.33***	9.51***
Fungal α-diversity	Richness	1.10	191.10***	4.02***
	Evenness	0.72	10.51***	2.23*
	Shannon	0.64	19.90***	2.46*

Significance of effects was assessed by two-way ANOVAs, with α = 0.05. ^1^Analyses were performed on normalized soil variables relevant to microbial life. OM, organic matter; Labile OC, water-extractable organic carbon; Labile N, water-extractable total nitrogen. ^2^Analyses were performed on log-transformed variables. GLS, β-glucosidases; XYL, β-xylosidases; AP, Acid phosphatases; NAG, N-acetyl-glucosaminidases; LAP, Leucine aminopeptidases; PP, Phenol peroxidases. ^3^Degrees of freedom of F-values were lower for respiration because rates with R^2^ < 0.8 were removed: Treatment F_2,34_, Soil origin F_3,34_, Treatment × Soil origin F_3,34_. ^4^Prokaryotic abundance: 16S gene copy number g^−1^ DW. ^5^Fungal abundance: ITS gene copy number g^−1^ DW. Numbers in bold and asterisks indicate significant differences between groups, with ***p < 0.001, **p < 0.01, *p < 0.05.

### Soil Microbial Activities

**FIGURE 1:**
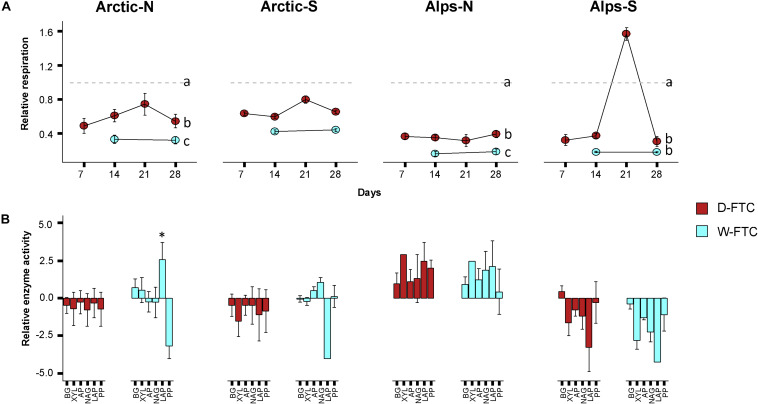
Microbial activities. **(A)** Basal respiration. Values are given as the ratio of the daily freeze-thaw cycles (D-FTC; red) or weekly freeze-thaw cycles (W-FTC; blue) divided by the +5°C controls. Lower-case letters represent statistically different groups. **(B)** Extracellular enzymatic activities. Relative activity is represented as the binary logarithm of the ratio of the daily freeze-thaw cycles (D-FTC) or the weekly freeze-thaw cycles (W-FTC) divided by the mean of the +5°C controls. Ratios were limited to the range of (–4, 4). BG, β-glucosidases; XYL, β-xylosidases; AP, Acid Phosphatases; NAG, N-acetyl-glucosaminidases; LAP, Leucine aminopeptidases; PP, Phenol Peroxidases. Mean ± SE (*n* = 4). Arctic, arctic soils; Alps, alpine soils; -N, north-exposed; -S, south-exposed. ^∗^significant difference of LAP, W-FTC vs. control, *F*_1,3_ = 6.160, *p* = 0.048. Dotted line represents the intercept with the value 0 in the *y* axis.

Freeze-thaw cycles had a strong influence on soil basal respiration (Table 1). Both the W-FTC and D-FTC treatments led to a decrease in the rates of basal respiration relative to the +5°C controls at the end of the incubation (day 28) in all but the south-exposed arctic soils (Figure 1 and Supplementary Tables S3, S4). The decrease in basal respiration was greater for the W-FTCs (range 31–66% across all soils) than for the D-FTCs (18–44%). These differences between the W-FTC and the D-FTC treatments were significant in the north-exposed soils of both Arctic and Alpine soils (*p* < 0.05, Figure 1 and Supplementary Table S3).

Extracellular enzymatic activities were measured to evaluate changes in microbial acquisition of C-, N-, and P-compounds. At the end of the incubation, extracellular enzymatic activities did not exhibit consistent response patterns across the four soils (Table 1 and Figure 1 and Supplementary Table S4). In the arctic soils, D-FTCs led to a general decrease in all enzymatic activities relative to the +5°C controls. The W-FTC treatment caused a significant increase in Leucine aminopeptidase activity in the north-exposed arctic soil, while it drastically decreased in the south-exposed soil. In the north-exposed alpine soils, both FTC treatments led to an increase in all enzymatic activities relative to the +5°C controls. In contrast, in the south-exposed alpine soils, all enzymatic activities decreased after the FTC treatments (Figure 1 and Supplementary Table S4).

### Microbial Abundance

Microbial abundance, measured by quantitative PCR of the 16S rRNA gene (prokaryotes) and the ITS2 (fungi) genomic regions, was not affected by the FTCs but by soil origin (Table 1). Arctic soils had a higher prokaryotic (ranging from 4 × 10^8^ to 2 × 10^9^ 16S gene copies g^–1^ DW) and fungal (2 × 10^6^ to 1 × 10^7^ ITS gene copies g^–1^ DW) abundance than alpine soils (5 × 10^4^ to 7 × 10^5^ 16S gene copies g^–1^ DW and 3 × 10^5^ to 3 × 10^6^ ITS gene copies g^–1^ DW; Supplementary Figure S4).

### Microbial Community Composition

The composition of the soil prokaryotic and fungal communities was revealed by amplicon sequencing of the 16S rRNA and ITS2 genomic regions, respectively. A total of 2 965 563 (39 021 ± 4899 per sample) prokaryotic and 2 216 494 (29 165 ± 5901) eukaryotic high-quality filtered reads were obtained, which clustered into 11 309 prokaryotic and 3976 eukaryotic OTUs. Prokaryotes mostly consisted of Bacteria (99.6% of prokaryotic reads, 11 270 OTUs), where most abundant phyla (>1% of total reads) were Proteobacteria, Chloroflexi, Acidobacteria, Verrucomicrobia, Planctomycetes, Actinobacteria, Bacteroidetes, Gemmatimonadetes, WD272 and Parcubacteria (Supplementary Table S5). Fungi (95% of eukaryotic reads, 3298 OTUs) mostly consisted of Ascomycota, Basidiomycota and Zygomycota [former classification, see Spatafora et al. (2016)] (Supplementary Table S5).

**FIGURE 2:**
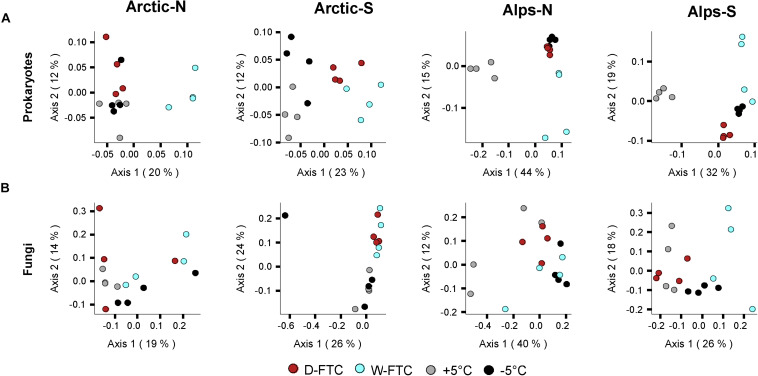
Microbial β-diversity of the incubated soils. PCoAs computed on Bray-Curtis dissimilarities based on OTU relative abundances of **(A)** Prokaryotes and **(B)** Fungi. Arctic, arctic soils; Alps, alpine soils; -N, north-exposed; -S, south-exposed. D-FTC: daily freeze-thaw cycles (red), W-FTC: weekly freeze-thaw cycles (blue), +5°C: controls +5°C (gray), –5°C: controls –5°C (black).

Similar to the physico-chemical variables, the microbial composition of the four soils, assessed at the beginning and at the end of the incubation experiment, primarily differed according to their soil origin (Supplementary Figure S3). FTCs showed a significant influence on prokaryotic α-diversity at the end of the incubation period (OTUs richness, Pielou’s evenness and Shannon’s diversity; *p* < 0.01; Table 1). However, when analyzed independently, changes in α-diversity linked to the FTCs treatments were minor, and they varied depending on their origin (Supplementary Figure S5 and Supplementary Table S3). FTCs showed clear effects on prokaryotic β-diversity (*p* < 0.001, Table 2), where the structure of prokaryotic communities from the FTC treatments (D-FTC and W-FTC) and the controls (+5°C and −5°C) were significantly different (*p* < 0.05, Table 2 and Figure 2). Overall, dissimilarities in β-diversity were larger between the W-FTC and the +5°C controls than between the D-FTC and the +5°C controls (Figure 2 and Supplementary Figure S6).

**TABLE 2:** Effects of treatment, soil origin and their interaction on microbial β-diversity.

	**Prokaryotes**								**Fungi**								
**PERMANOVA**																	
	**DF**	**SS**		**MS**	**F^1^**	**R2**		***p***	**DF**	**SS**		**MS**		**F**	**R2**		***p***
Treatment	3	0.389		0.130	8.436	0.041		**<0.001^2^**	3	0.574		0.191		2.047	0.026		**0.013**
Soil origin	3	7.891		2.630	171.151	0.838		**<0.001**	3	15.723		5.241		56.101	0.706		**<0.001**
Treatment × Soil origin	9	0.395		0.044	2.853	0.042		**<0.001**	9	1.475		0.164		1.754	0.066		**0.004**
Residuals	48	0.738		0.015		0.078			48	4.484		0.093			0.201		
Total	63	9.413				1			63	22.256					1		

**Pairwise PERMANOVA**																	
	**Arctic**				**Alps**				**Arctic**					**Alps**			
	**N**		**S**		**N^3^**		**S**		**N**		**S**			**N**		**S^3^**	
	**F**	***p***	**F**	***p***	**F**	***p***	**F**	***p***	**F**	***p***	**F**		***P***	**F**	***p***	**F**	***P***

D-FTC vs. +5°C	1.793	**0.028**	2.829	0.028	9.189	**0.029**	8.15	**0.029**	1.014	0.488	2.636		**0.028**	1.874	0.144	1.099	0.309
W-FTC vs. +5°C	1.900	**0.030**	3.571	**0.029**	8.543	**0.031**	6.53	**0.029**	2.176	0.027	3.442		**0.028**	2.188	0.166	2.242	0.06
D-FTC vs. −5°C	3.174	**0.028**	1.991	**0.028**	3.997	**0.03**	5.008	**0.027**	1.19	0.227	1.572		**0.03**	2.036	**0.032**	2.702	0.03
W-FTC vs. −5°C	1.590	0.055	2.851	**0.028**	3.689	**0.027**	2.989	**0.028**	0.952	0.518	1.818		**0.03**	1.266	0.14	1.728	0.059
D-FTC vs. W-FTC	2.971	**0.026**	1.573	0.057	3.325	**0.03**	4.044	**0.028**	1.207	0.258	1.23		0.201	1.098	0.262	2.776	**0.03**
+5°C vs. −5°C	3.078	**0.026**	1.717	**0.032**	10.413	**0.029**	9.175	**0.032**	1.466	0.03	1.002		0.482	4.755	**0.027**	2.365	**0.03**

*General effects of treatment and soil origin on β-diversity were assessed by PERMANOVA tests. Differences among treatments within each four different soils were assessed by pairwise PERMANOVAs, with α = 0.05. ^1^ F correspond to the pseudo-F ratio. ^2^ Numbers in bold indicate *p* < 0.05. ^3^ Based on Chord distances to meet the condition of homogeneity of dispersion between groups. DF, Degrees of freedom; SS, Sum of squares. MS, mean squares. D-FTC, daily freeze-thaw cycles; W-FTC, weekly freeze-thaw cycles; +5°C, controls +5°C; −5°C, controls −5°C; Arctic, arctic soils; Alps, alpine soils; -N, north-exposed; -S, south-exposed.*

In contrast, fungal communities were barely affected by the FTC treatments (Tables 1, 2 and Figure 2). FTCs had no impact on fungal α-diversity (Supplementary Figure S5 and Supplementary Table S3), and significant differences in fungal β-diversity between D-FTCs, W-FTCs and controls were only detected for the south-exposed arctic soils (*p* < 0.05, Table 2).

### Indicator Taxa Associated With FTCs

**FIGURE 3:**
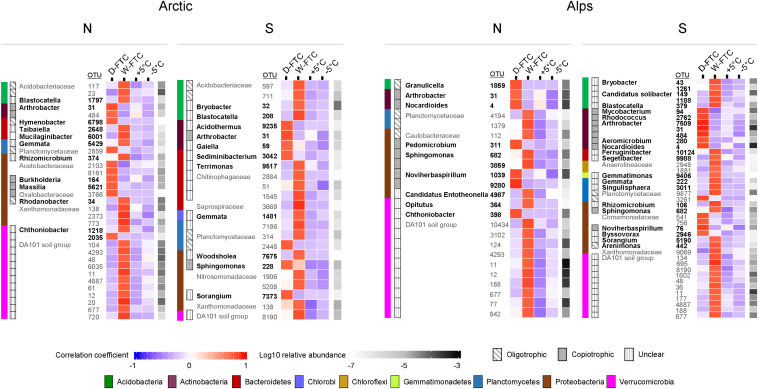
Bacterial indicators OTUs significantly associated to the daily freeze-thaw cycles (D-FTC) or the weekly freeze-thaw cycles (W-FTC) but not associated with the +5°C controls (+5°C) for the four different soils. Indicator taxa with and *indicator value index* to the W-FTC or the D-FTC greater than 0.75 and assigned beyond the family level are represented. Classification in *oligotrophic* and *copiotrophic* is based on the classifications in bacterial life strategies proposed by Fierer et al. (2007) and Ho et al. (2017), and on information about ecological and cultivation traits of bacterial groups compiled by Rosenberg et al. (2013). *Unclear* refers to taxa with insufficient information (taxa with fewer than 10 cultivated strains recorded in the NCBI database or candidate taxa) to be classified to a life strategy. Families containing both oligotrophic and copiotrophic taxa were not assigned a life strategy. Names in bold correspond to Genus taxonomic levels. –5°C: controls –5°C. Arctic, arctic soils; Alps, alpine soils; -N, north-exposed; -S, south-exposed.

*Indicator species analyses* were conducted for each soil independently to identify bacterial and fungal OTUs significantly associated with the D-FTC and W-FTC treatments at the end of the incubation. A total of 988 prokaryotic (8% of total prokaryotic OTUs) and 130 fungal OTU indicators (4% of total fungal OTUs) were significantly associated with the FTC treatments (*p* < 0.05). Only one bacterial indicator OTU (Figure 3, OTU 31, *Arthrobacter*) was common to all four soils. D-FTC samples were enriched in bacterial indicator OTUs from Actinobacteria, a phylum with numerous members with copiotrophic lifestyles (Figure 3 and Supplementary Table S6). Moreover, indicator OTUs from copiotrophic genera such as *Arthrobacter, Nocardioides* (Actinobacteria), *Noviherbaspirillum* (Proteobacteria), *Masillia, Burkholderia* (Proteobacteria), and *Rhodococcus* showed strong associations (≥0.75 indicator value index) with the D-FTCs and were negatively associated with the W-FTCs (Figure 3). In contrast, W-FTC treatments were enriched in indicator OTUs from oligotrophic taxa, such as those in Verrucomicrobia (mainly DA101), Acidobacteria, Planctomycetes, Parcubacteria and WD272 phyla (Figure 3 and Supplementary Table S6). Indicator OTUs from oligotrophic genera such as *Blastocatella, Bryobacter* (Acidobacteria), and *Gemmata* showed strong associations (≥0.75) with the W-FTCs and were negatively associated with the D-FTCs (Figure 3).

**FIGURE 4:**
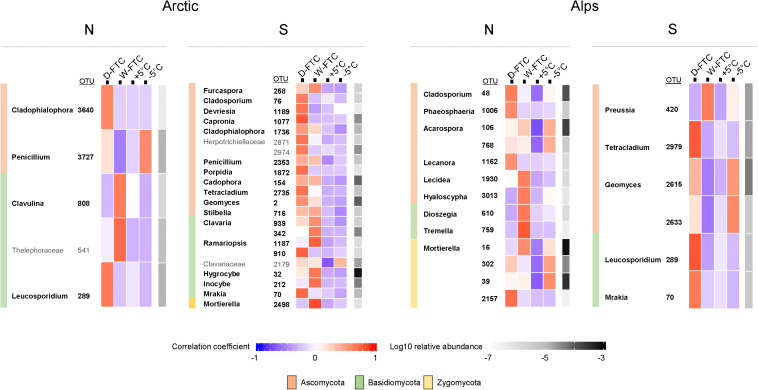
Fungal indicators OTUs significantly associated to the daily freeze-thaw cycles (D-FTC) or the weekly freeze-thaw cycles (W-FTC) but not associated with the +5°C controls (+5°C) for the four different soils. Indicators assigned beyond the family level are displayed. Names in bold correspond to Genus. –5°C: controls –5°C. Arctic, arctic soils; Alps, alpine soils; -N, north-exposed; -S: south-exposed.

Fungal indicator OTUs were mostly composed of saprotrophic and pathogenic taxa (Figure 4 and Supplementary Table S7). These included ubiquitous cold-tolerant and opportunistic genera such as *Mrakia*, *Geomyces, Mortierella, Dioszegia*, *Leucosporidium*, and *Penicillium* (Figure 4). Interestingly, several indicator OTUs associated with the FTC treatments were also associated with the −5°C controls, especially *Mortierella* and *Geomyces* in the alpine soils (Figure 4). Complete information and statistics for all prokaryotic and fungal indicator OTUs at various taxonomical levels from phylum to genus are provided in Supplementary Tables S8, S9.

## Discussion

To our knowledge, this is the first experimental study comparing the effects of repeated FTCs between arctic and alpine soils, and in which changes on both taxonomical and functional components of the soil microbial communities are analyzed at fine-scale resolution. We acknowledge that our analyses of the microbial communities are based on DNA and thereby include both active and inactive microorganisms. Nevertheless, by comparing the highly frequent daily FTCs with the infrequent weekly FTCs and including compositional and functional data, we provide an integrative overview of potential dynamic responses that arctic and alpine soil microbiomes might undergo in the future, as snow becomes scarce and FTCs become more frequent.

Repeated FTCs have been shown to cause large alterations in the soil microbiota (Schimel and Clein, 1996; Feng et al., 2007; Han et al., 2018). We therefore hypothesized that shorter and more frequent FTCs (daily; D-FTCs) would cause larger compositional changes on the soil prokaryotic and fungal communities than longer and less frequent FTCs (weekly; W-FTCs). However, we observed the opposite: although both FTCs treatments led to significant shifts in β-diversity in the prokaryotic communities compared with the thaw controls, effects were larger for the W-FTCs than for the D-FTCs. Furthermore, we identified an apparent trait-based pattern of response of the prokaryotic communities to the tested FTC frequencies: D-FTCs stimulated copiotrophic and opportunistic bacteria over oligotrophic taxa while W-FTCs promoted oligotrophic bacteria over copiotrophs. We use the designation of “copiotrophs” for fast-growing and metabolically versatile microbial taxa, in opposition to “oligotroph,” which refers to slow-growing, stress-tolerant microorganisms that thrive on low nutrient concentrations (Koch, 2001). The categorization of microbial phylogenetic groups into copiotrophs or oligotrophs is, however, debatable (Ho et al., 2017), since physiological traits in microorganisms are context-dependent and they are highly variable, even at the deepest taxonomical level of species (Lauro et al., 2009; Morrissey et al., 2016; Ho et al., 2017). Nevertheless, we apply the categorization copiotroph-oligotroph in our study, since this categorization has been proven to be useful for describing general trends of association between microbial composition and function, and their ecological meaning in natural soil systems (Meisner et al., 2018; Schostag et al., 2019).

In the D-FTC treatment, the freezing phase was short (12 h) and freeze-thawing occurred daily. In each cycle, freeze-thawing causes the lysis of FTC-sensitive cells and the mobilization of labile carbon and nutrient sources from the physical disaggregation of soil organic materials (Feng et al., 2007; Wang et al., 2012). In this scenario, we propose that copiotrophic microorganisms outcompeted oligotrophs because they were able to withstand environmental changes and grow faster during the thawing phases by rapidly utilizing the newly labile substrates (Koch, 2001; Fierer et al., 2007). This could explain the increase in the number of indicator OTUs associated with the D-FTCs from fast-growing genera such as *Arthrobacter*, *Massilia*, and *Rhodococcus. Arthrobacter* sp., which was observed in all four soils, exhibit a high diversity of cold-shock proteins and adjust quickly to temperature changes and cooling (Berger et al., 1996). *Arthrobacter* sp. and *Massilia* grow well in nutrient-amended media (Lee et al., 2004; Liu et al., 2018), and RISA fingerprinting has shown that *Rhodococcus* becomes dominant in arctic soils exposed to 24-h FTCs (Eriksson et al., 2001).

In the longer W-FTC treatment, freeze-thawing occurred only twice, and the freezing phase lasted for 7 days, compared to 12 h in the D-FTC treatment. Prolonged freezing leads to conditions of desiccation and starvation in the soil (Panoff et al., 1998; Margesin, 2012), and it triggers the activation of complex cell survival mechanisms (Walker et al., 2006; De Maayer et al., 2014; Koponen and Baath, 2016), including extensive synthesis of cold-shock proteins and cryoprotectants, which are energetically costly (De Maayer et al., 2014). Oligotrophs have lower growth rates than copiotrophs but they use nutritional resources more efficiently, thus requiring less energy to grow (Koch, 2001; Fierer et al., 2007). Therefore, oligotrophic microorganisms could have survived the freezing phases of the W-FTCs in better physiological conditions or in larger numbers than the copiotrophs, thus taking over during the thawing phase.

The higher tolerance of oligotrophic microorganisms to freezing compared to copiotrophic taxa could explain the higher proportion of indicator OTUs associated with the W-FTCs treatment from phyla such as Verrucomicrobia, Acidobacteria, Planctomycetes, and Parcubacteria, which, based on current knowledge (Fierer et al., 2007; Ho et al., 2017), are enriched in oligotrophic members. Members belonging to Verrucomicrobia and Acidobacteria seem to thrive in nutrient-depleted media (Sangwan et al., 2005; Tanaka et al., 2017). In addition, Acidobacteria and Parcubacteria have been shown to be abundant in permafrost (Steven et al., 2007), where microbial activity has been reported (Coolen and Orsi, 2015; Nikrad et al., 2016) despite the permanent freezing conditions. Furthermore, members from Acidobacteria phyla has been shown to increase in abundance in boreal wetland soils subjected to slow freeze-thawing (Ren et al., 2018). At finer taxonomical level, indicator OTUs associated with the W-FTCs mainly classified to uncultured genera (e.g., *Blastocatella* and *Gemmata*), which highlights the special growing requirements of these taxa and the vast microbial diversity still unknown in alpine and arctic soils.

In a future climate with less snow, the occurrence of shorter and more frequent FTCs might be disadvantageous for taxa with oligotrophic attributes in arctic and alpine soils, which, if failing to adapt, could be overtaken by copiotrophic groups. In addition, copiotrophic microorganisms would further benefit from the increase in soil temperatures, as observed in studies on permafrost affected soils (including the active layer) (Schostag et al., 2015; Luláková et al., 2019) and glacier forefields (Rime et al., 2015; Kim et al., 2017).

The weak response of the fungal communities to the FTC treatments is in line with previous studies (Sharma et al., 2006; Han et al., 2018) and might be attributed to the high tolerance of fungi to temperature fluctuations (Sharma et al., 2006) and their ability to endure freezing conditions (Lipson et al., 2002; Ozerskaya et al., 2009). In our study, we observed an accumulation of fungal indicator OTUs from cold-adapted, opportunistic genera, such as *Mortierella*, *Mrakia*, and *Leucosporidium*, in association with both the D-FTC and W-FTC treatments. *Mortierella* sp. have been reported to grow at subzero temperatures (Schmidt et al., 2008) and to be dominant in alpine and subalpine ecosystems (Schmidt et al., 2012; Adamczyk et al., 2019). The basidiomycetous yeasts *Mrakia* and *Leucosporidium* have been reported to be abundant in permafrost (Frey et al., 2016). Thus, alterations within arctic and alpine soil fungal communities subsequent to FTCs might only be relevant after years of ongoing variations in the soil environmental conditions linked to climate change (Rinnan et al., 2007; Xiong et al., 2014).

In agreement with our second hypothesis, the compositional shifts caused by the FTCs were accompanied by an overall decrease in the rates of soil basal respiration. However, this decrease in respiration was not associated with changes in microbial abundances, thereby suggesting a drop in microbial activity (Grogan et al., 2004; Bolter et al., 2005; Sharma et al., 2006; Stres et al., 2010), which could have resulted from the slowing down of metabolic processes of microbial cells acclimatizing to freezing conditions. The stress that the prolonged freezing conditions pose to the microbial cells could be reflected in the higher levels of labile nitrogenous compounds detected in the alpine soils subjected to W-FTCs, associated to the increase in necromass (Schimel and Clein, 1996; Han et al., 2018). The lack of changes in microbial abundance together with the larger shifts in microbial composition in the W-FTCs treatments could indicate higher turnover rates, where the dying cells would be rapidly replaced by the microorganisms tolerant to freezing. Further experiments targeting specifically the active microbial populations (i.e., RNA based) and assessing growth rates would be necessary to test how turnover rates are associated with changes in microbial abundance and composition caused by FTCs.

Freeze-thaw cycles provoked only weak changes in C-, N-, and P-acquiring enzymatic activities, they did not affect the concentrations of soil OM, and they altered labile C only to a very limited extent. These results contrast with previously published findings that report an increase in labile organics in the soil along with a shift in OM microbial degradation processes subsequent to FTCs, from the utilization of complex substrates contained in the OM to the consumption of simple compounds (Schimel and Mikan, 2005; Han et al., 2018). The large differences in soil properties and microbial diversity among the different soils could explain the inconsistent changes in enzymatic activities observed across the FTC treatments. The influence of multiple soil factors on the synthesis of C-, N-, and P-acquiring enzymatic activities could have overridden the possible effects of FTCs in the measured enzymatic activities. The synthesis of extracellular enzymes is regulated by the interplay of multiple factors, including the content and quality of the OM and the specific energetic and nutritional demands of the microbial community members (Sinsabaugh et al., 2009; Burns, 2010; Steinweg et al., 2013; Kim, 2015; Mooshammer et al., 2017). This notwithstanding, the decoupling of the changes in microbial structure associated with the FTCs from the shifts in C-, N-, and P-acquiring enzymatic activities might additionally be due to functional redundancy (Frossard et al., 2012; Mooshammer et al., 2017).

In our third hypothesis, we predicted that soils with a legacy of more frequent FTCs harbor microbial communities more tolerant to the repeated shorter and more frequent FTCs (e.g., microbial communities from south-exposed soils were expected to be better adapted to frequent FTCs than those from north-exposed soils), as proposed previously (Yergeau and Kowalchuk, 2008; Stres et al., 2010). However, in the present study, soil FTC legacy did not show a clear influence on the tolerance of the microbial communities to the tested FTCs of distinct frequencies. This observation could indicate that FTCs might be governed by common climatological factors between alpine and arctic soil ecosystems, such as the dominance of subzero temperatures and snow coverage, which are known to shape microbial adaptations to temperature variations (Koponen and Baath, 2016). Further investigations will be needed to identify such factors and understand their influence on the local and global microbial responses of arctic and alpine soils to FTCs in a changing world.

## Conclusion

Overall, our integrative investigation of the impact of FTC frequency on the structure and functionality of microbial communities of arctic and alpine soils suggested a trait-based response of prokaryotic communities to FTC frequency, where repeated short and frequent FTCs seemed to benefit copiotrophic bacteria while longer and less frequent FTCs seemed to promote oligotrophic bacteria. Opportunistic, cold-adapted fungi were prevalent after FTCs. Compositional shifts were coupled with a decline in microbial respiration rates, which was larger in the soils exposed to longer and less frequent FTCs. Changes in specific metabolic responses (C-, N-, and P-acquiring extracellular enzymes) to FTCs were heterogenous among the soils, and microbial tolerance to FTCs may be modulated by climatological factors common to arctic and alpine regions rather than by FTC soil legacies. Although limited to the comparison of only two sites, our findings indicate that future climate scenarios with projected snow-scarce winters in the Arctic and the Alps, and therefore more frequent FTCs, could lead to noticeable compositional and functional shifts in the soil microbiome from both ecosystems. In turn, this could result on potential changes in OM quality, soil C stocks, and soil CO_2_ fluxes at the ecosystem level.

## Data Availability Statement

All raw sequencing data generated for this study have been deposited in the NCBI Sequence Read Archive under the BioProject accession identifier PRJNA535397.

## Author Contributions

CP-M, AF, and BF designed the study. CP-M and BF participated in sample collection. CP-M performed the experiment and wrote the manuscript with the help of AF and BF.

## Conflict of Interest

The authors declare that the research was conducted in the absence of any commercial or financial relationships that could be construed as a potential conflict of interest.
