# Supplementary material for: Functional and Structural Responses of Arctic and Alpine Soil Prokaryotic and Fungal Communities Under Freeze-Thaw Cycles of Different Frequencies
Source: Front Microbiol. 2020 May 25;11:982. doi: 10.3389/fmicb.2020.00982 (PMC7261861; doi:10.3389/fmicb.2020.00982)
Supplement: Supplementary file 7 [file Data_Sheet_1.docx]

**SUPPLEMENTARY INFORMATION**

Functional and structural responses of arctic and alpine soil prokaryotic and fungal communities under freeze-thaw cycles of different frequencies

**Carla Perez-Mon, Beat Frey, Aline Frossard***

Forest Soils and Biogeochemistry, Swiss Federal Institute for Forest, Snow and Landscape Research WSL, Birmensdorf, Switzerland

∗**Corresponding author:** Aline Frossard, Swiss Federal Research Institute WSL, Zürcherstrasse 111, CH-8903 Birmensdorf, Switzerland. Tel: +41-44-739-2807; E-mail: aline.frossard@wsl.ch

**SUPPLEMENTARY FIGURES LEGENDS**

**Fig. S1.** Scheme of the study design. Soil sample groups (each with four replicates) were subjected to (1) daily freeze-thaw cycles (D-FTC, 12h +5°C / 12h -5°C; red line); (2) weekly freeze-thaw cycles (W-FTC, 7 days +5°C / 7 days -5°C; blue line); (3) constant +5°C conditions (+5°C; gray line); or (4) constant -5°C conditions (-5°C; black line). Basal respiration (CO_2_) was measured once per week during the incubation period (28 days total).

**Fig. S2.** Yearly patterns of soil surface temperatures in the studied sites: Latnjachorru (Arctic) and Muot de Barba Peider (Alps). Values correspond to hourly temperature means; n=5 for the alpine soils and n=2 for the arctic soils. FTCs occur during the transitions from autumn to winter and spring to summer, as shown at higher time resolution in the lower part of the figure (Mean ± SD).

**Fig. S3.** Differences among the four soils at the beginning of the experiment. **(A)** PCA computed on normalized soil physico-chemical variables (Table S1), **(B)** PCoA computed on Bray-Curtis dissimilarities based on prokaryotic OTU relative abundances, **(C)** PCoA computed on Bray-Curtis dissimilarities based on fungal OTU relative abundances. Arctic: arctic soils, Alps: alpine soils, -N: north-exposed, -S: south-exposed.

**Fig. S4.** Prokaryotic **(A)** and fungal **(B)** abundances. Abundance was measured as log10 of 16S gene copy number g^-1^ DW (prokaryotic abundance) and ITS gene copy number g^-1^ DW (fungal abundance). Mean ± SE (n=4). Arctic: arctic soils, Alps: alpine soils, -N: north-exposed, -S: south-exposed. D-FTC: daily freeze-thaw cycles (red), W-FTC: weekly freeze-thaw cycles (blue), +5°C: controls +5°C (gray), -5°C: controls -5°C (black).

**Fig. S5.** α-diversity of the prokaryotic and fungal communities for each treatment and soils. OTUs Richness **(A** and **B)**, Pielou’s evenness index **(C** and **D)** and Shannon diversity index **(E** and **F)**. Asterisks over lines indicate statistically different groups (p < 0.05). Mean ± SE (n=4). Arctic: arctic soils, Alps: alpine soils, -N: north-exposed, -S: south-exposed. D-FTC: daily freeze-thaw cycles (red), W-FTC: weekly freeze-thaw cycles (blue), +5°C: controls +5°C (gray), -5°C: controls -5°C (black).

**Fig. S6.** Dissimilarities between FTC treatments and controls. Bray-Curtis dissimilarity between daily freeze-thaw cycles (D-FTC) and controls +5°C (+5) and weekly freeze-thaw cycles and controls +5°C. Mean ± SD (n=4).

**SUPPLEMENTARY TABLES**

**Table S1.** Physico-chemical properties of the studied soils. Mean ± SD (n=3).

|  | **Arctic** | | **Alps** | |
| --- | --- | --- | --- | --- |
|  | **N** | **S** | **N** | **S** |
| **Sand [%]** | 66.8 ± 0.14 | 66.8 ± 1.13 | 89.5 ± 0.00 | 86.3 ± 0.14 |
| **Silt [%]** | 27.30 ± 0.42 | 28.30 ± 0.99 | 7.00 ± 0.42 | 11.10 ± 0.28 |
| **Clay [%]** | 5.90 ± 0.28 | 4.90 ± 0.14 | 3.50 ± 0.42 | 2.60 ± 0.14 |
| **pH [CaCl_2_]** | 4.24 ± 0.01 | 4.00 ± 0.05 | 4.59 ± 0.02 | 4.52 ± 0.01 |
| **WHC [g H_2_O g^-1^]** | 0.46 ± 0.09 | 0.95 ± 0.21 | 0.22 ± 0.02 | 0.30 ± 0.01 |
| **WC [g H_2_O g^-1^]** | 0.25 ± 0.02 | 0.48 ± 0.01 | 0.07 ± 0.00 | 0.12 ± 0.01 |
| **C [%]** | 1.33 ± 0.02 | 4.05 ± 0.05 | 0.34 ± 0.00 | 0.82 ± 0.01 |
| **N [%]** | 0.08 ± 0.00 | 0.23 ± 0.00 | 0.03 ± 0.00 | 0.05 ± 0.00 |
| **OM [%]** | 2.43 ± 0.07 | 7.66 ± 0.55 | 1.13 ± 0.05 | 2.45 ± 0.18 |

WHC: water holding capacity

WC: water content

OM: organic matter

Arctic: arctic soils, Alps: alpine soils, -N: north-exposed, -S: south-exposed

**Table S2.** Chemical properties in the soils at the end of the incubation for each FTCs treatment. Mean ± SD (n=4). Pair-wise statistical differences among treatments were assessed by Tukey HSD post-hoc tests in each subset of soils separately. Values in bold with different superscript letters represent groups in which differences were statistically significant (p < 0.05).

|  | **Treatment** |  | **pH** | | |  | **OM^1^** | | |  | **labile OC^2^** | | |  | **labile N^3^** | | |  | **Ammonium** | | |  | **Nitrate^4^** | | |  | **Nitrite** | | |
| --- | --- | --- | --- | --- | --- | --- | --- | --- | --- | --- | --- | --- | --- | --- | --- | --- | --- | --- | --- | --- | --- | --- | --- | --- | --- | --- | --- | --- | --- |
|  |  |  |  |  |  |  |  |  |  |  |  |  |  |  |  |  |  |  |  |  |  |  |  |  |  |  |  |  |  |
| **Arctic-N** | D-FTC |  | 4.26 | **±** | 0.01 |  | 2.23 | ± | 0.11 |  | **57.744** | ± | **15.79^ab^** |  | 1.01 | ± | 0.53 |  | 0.71 | ± | 0.49 |  | 0.84 | ± | 0.56 |  | b.d.l |  |  |
|  | W-FTC |  | 4.27 | **±** | 0.02 |  | 2.33 | ± | 0.10 |  | **44.91** | ± | **10.45^b^** |  | 1.11 | ± | 0.53 |  | 0.64 | ± | 0.25 |  | 3.35 | ± | 3.87 |  | 0.26 | ± | 0.00 |
|  | +5°C |  | 4.22 | **±** | 0.01 |  | 2.40 | ± | 0.06 |  | **103.04** | ± | **31.82^a^** |  | 1.32 | ± | 0.39 |  | 0.63 | ± | 0.23 |  | 0.59 | ± | 0.12 |  | b.d.l |  |  |
|  | -5°C |  | 4.28 | **±** | 0.06 |  | 2.19 | ± | 0.17 |  | **46.73** | ± | **11.10^b^** |  | 0.99 | ± | 0.28 |  | 0.69 | ± | 0.16 |  | 1.38 | ± | 0.74 |  | 0.16 | ± | 0.07 |
| **Arctic-S** | D-FTC |  | 4.15 | ± | 0.07 |  | 8.79 | ± | 0.32 |  | 156.42 | ± | 30.76 |  | 11.45 | ± | 1.89 |  | **6.42** | **±** | **1.45^a^** |  | 4.63 | ± | 1.32 |  | 0.35 | ± | 0.15 |
|  | W-FTC |  | 4.18 | ± | 0.11 |  | 8.54 | ± | 0.21 |  | 134.97 | ± | 15.29 |  | 12.58 | ± | 0.84 |  | **7.02** | **±** | **0.87^a^** |  | 12.03 | ± | 0.72 |  | 0.2 | ± | 0.05 |
|  | +5°C |  | 4.00 | ± | 0.03 |  | 7.38 | ± | 0.22 |  | 133.87 | ± | 28.58 |  | 9.26 | ± | 3.17 |  | **3.63** | **±** | **1.10^ab^** |  | 8.81 | ± | 6.48 |  | 0.67 | ± | 0.32 |
|  | -5°C |  | 4.09 | ± | 0.08 |  | 7.71 | ± | 0.74 |  | 145.90 | ± | 22.88 |  | 12.28 | ± | 3.00 |  | **5.85** | **±** | **0.71^b^** |  | 4.14 | ± | 1.44 |  | 0.33 | ± | 0.12 |
|  |  |  |  |  |  |  |  |  |  |  |  |  |  |  |  |  |  |  |  |  |  |  |  |  |  |  |  |  |  |
| **Alps-N** | D-FTC |  | 4.57 | ± | 0.02 |  | 1.24 | ± | 0.05 |  | 42.20 | ± | 22.08 |  | **0.62** | ± | **0.43^c^** |  | 0.63 | ± | 0.36 |  | b.d.l |  |  |  | b.d.l |  |  |
|  | W-FTC |  | 4.57 | ± | 0.01 |  | 1.22 | ± | 0.04 |  | 43.60 | ± | 14.92 |  | **2.73** | ± | **0.60^b^** |  | 0.48 | ± | 0.05 |  | 6.14 | ± | 3.81 |  | 0.32 | ± | 0.07 |
|  | +5°C |  | 4.60 | ± | 0.02 |  | 1.25 | ± | 0.03 |  | 27.80 | ± | 7.50 |  | **0.66** | ± | **0.29^c^** |  | 0.35 | ± | 0.08 |  | b.d.l |  |  |  | b.d.l |  |  |
|  | -5°C |  | 4.57 | ± | 0.00 |  | 1.22 | ± | 0.05 |  | 30.54 | ± | 10.46 |  | **3.96** | ± | **0.82^a^** |  | 0.73 | ± | 0.37 |  | 6.03 | ± | 2.73 |  | 0.33 | ± | 0.18 |
| **Alps-S** | D-FTC |  | 4.51 | ± | 0.01 |  | 2.27 | **±** | 0.17 |  | 32.04 | ± | 10.62 |  | **0.57** | ± | **0.46^c^** |  | 0.66 | ± | 0.26 |  | b.d.l | ± |  |  | b.d.l |  |  |
|  | W-FTC |  | 4.51 | ± | 0.01 |  | 2.33 | **±** | 0.04 |  | 42.07 | ± | 43.13 |  | **2.26** | ± | **0.59^b^** |  | 0.79 | ± | 0.14 |  | 4.03 | ± | 2.83 |  | 0.39 | ± | 0.26 |
|  | +5°C |  | 4.48 | ± | 0.00 |  | 2.15 | **±** | 0.05 |  | 32.04 | ± | 8.00 |  | **0.55** | ± | **0.33^c^** |  | 0.62 | ± | 0.28 |  | b.d.l | ± |  |  | b.d.l |  |  |
|  | -5°C |  | 4.50 | ± | 0.01 |  | 2.22 | **±** | 0.08 |  | 22.88 | ± | 7.91 |  | **3.32** | ± | **0.21^a^** |  | 0.90 | ± | 0.17 |  | 5.16 | ± | 1.46 |  | 0.37 | ± | 0.09 |

^1^OM: organic matter, % DW

^2^Labile OC: water-extractable organic carbon

^3^Labile N: water-extractable total nitrogen

^4^Nitrate and Nitrite were measured for the soil water extracts with a Dionex Integrion high pressure chromatograph equipped with an AS9-HC 2*50 mm Viper Fitting pre-column coupled with an AS9-HC 2*250 mm Viper Fitting column (Thermo Fisher Scientific, Waltham, Massachusetts, USA).

Labile OC, Labile N, Ammonium, Nitrate and Nitrite are measured in µg g^-1^ DW

Arctic: arctic soils, Alps: alpine soils, -N: north-exposed, -S: south-exposed

D-FTC: daily freeze-thaw cycles, W-FTC: weekly freeze-thaw cycles, +5°C: controls +5°C, -5°C: controls -5°C

b.d.l.: below detection limit

**Table S3.** Tukey HSD post-hoc tests on differences among FTCs treatments for soil properties, microbial activities and microbial α-diversity parameters. Tests were performed in each subset of soils separately. Only cases in which significant differences between pairs were detected are shown. α= 0.05

|  | | **Arctic-N** | | | | **Arctic-S** | | | | **Alps-N** | | | | **Alps-S** | | | |
| --- | --- | --- | --- | --- | --- | --- | --- | --- | --- | --- | --- | --- | --- | --- | --- | --- | --- |
|  | **Pairs** | **diff^1^** | **lwr^2^** | **upr^3^** | ***p*** | **diff** | **lwr** | **upr** | **p.adj** | **diff** | **lwr** | **upr** | **p.adj** | **diff** | **lwr** | **upr** | ***p*** |
| **Labile OC^4^** | **D-FTC vs +5°C** | -0.57 | -1.15 | 0.01 | 0.06 |  |  |  |  |  |  |  |  |  |  |  |  |
|  | **W-FTC vs +5°C** | -0.81 | -1.39 | -0.23 | **0.01** |  |  |  |  |  |  |  |  |  |  |  |  |
|  | **D-FTC vs -5°C** | 0.20 | -0.38 | 0.78 | 0.73 |  |  |  |  |  |  |  |  |  |  |  |  |
|  | **W-FTC vs -5°C** | -0.04 | -0.62 | 0.54 | 1.00 |  |  |  |  |  |  |  |  |  |  |  |  |
|  | **D-FTC vs W-FTC** | -0.25 | -0.83 | 0.33 | 0.60 |  |  |  |  |  |  |  |  |  |  |  |  |
|  | **+5°C vs -5°C** | -0.77 | -1.35 | -0.19 | **0.01** |  |  |  |  |  |  |  |  |  |  |  |  |
|  |  |  |  |  |  |  |  |  |  |  |  |  |  |  |  |  |  |
| **Labile N^5^** | **D-FTC vs +5°C** |  |  |  |  |  |  |  |  | -0.04 | -1.24 | 1.15 | 1.00 | 0.02 | -0.87 | 0.91 | 1.00 |
|  | **W-FTC vs +5°C** |  |  |  |  |  |  |  |  | 2.07 | 0.87 | 3.26 | 0.00 | 1.71 | 0.83 | 2.60 | **0.00** |
|  | **D-FTC vs -5°C** |  |  |  |  |  |  |  |  | -3.35 | -4.54 | -2.15 | 0.00 | -2.75 | -3.63 | -1.86 | **0.00** |
|  | **W-FTC vs -5°C** |  |  |  |  |  |  |  |  | -1.24 | -2.43 | -0.04 | 0.04 | -1.06 | -1.94 | -0.17 | **0.02** |
|  | **D-FTC vs W-FTC** |  |  |  |  |  |  |  |  | 2.11 | 0.91 | 3.31 | 0.00 | 1.69 | 0.81 | 2.58 | **0.00** |
|  | **+5°C vs -5°C** |  |  |  |  |  |  |  |  | 3.30 | 2.10 | 4.50 | 0.00 | 2.77 | 1.88 | 3.65 | **0.00** |
|  |  |  |  |  |  |  |  |  |  |  |  |  |  |  |  |  |  |
| **Ammonium** | **D-FTC vs +5°C** |  |  |  |  | 2.79 | 0.54 | 5.04 | **0.01** |  |  |  |  |  |  |  |  |
|  | **W-FTC vs +5°C** |  |  |  |  | 3.39 | 1.14 | 5.64 | **0.00** |  |  |  |  |  |  |  |  |
|  | **D-FTC vs -5°C** |  |  |  |  | 0.58 | -1.67 | 2.82 | 0.87 |  |  |  |  |  |  |  |  |
|  | **W-FTC vs -5°C** |  |  |  |  | 1.18 | -1.07 | 3.42 | 0.44 |  |  |  |  |  |  |  |  |
|  | **D-FTC vs W-FTC** |  |  |  |  | 0.60 | -1.65 | 2.85 | 0.86 |  |  |  |  |  |  |  |  |
|  | **+5°C vs -5°C** |  |  |  |  | 2.21 | -0.03 | 4.46 | 0.05 |  |  |  |  |  |  |  |  |
|  |  |  |  |  |  |  |  |  |  |  |  |  |  |  |  |  |  |
| **Respiration** | **W-FTC vs +5°C** | -1.56E-07 | -2.25E-07 | -8.65E-08 | **5.21E-04** |  |  |  |  | -2.14E-07 | -2.88E-07 | -1.40E-07 | **8.79E-05** | -2.40E-07 | -2.94E-07 | -1.86E-07 | **1.52E-06** |
|  | **D-FTC vs +5°C** | -2.32E-07 | -2.96E-07 | -1.68E-07 | **1.71E-05** |  |  |  |  | -2.87E-07 | -3.60E-07 | -2.13E-07 | **1.01E-05** | -2.85E-07 | -3.38E-07 | -2.31E-07 | **3.56E-07** |
|  | **D-FTC vs W-FTC** | -7.63E-08 | -1.45E-07 | -7.19E-09 | **3.24E-02** |  |  |  |  | -7.27E-08 | -1.41E-07 | -4.40E-09 | **3.82E-02** | -4.48E-08 | -9.86E-08 | 8.98E-09 | 1.03E-01 |
|  |  |  |  |  |  |  |  |  |  |  |  |  |  |  |  |  |  |
| **Prokaryotic Richness** | **D-FTC vs +5°C** |  |  |  |  |  |  |  |  | 179.00 | -4.55 | 362.55 | 0.06 |  |  |  |  |
|  | **W-FTC vs +5°C** |  |  |  |  |  |  |  |  | 170.75 | -12.80 | 354.30 | 0.07 |  |  |  |  |
|  | **D-FTC vs -5°C** |  |  |  |  |  |  |  |  | -297.50 | -481.05 | -113.95 | **0.00** |  |  |  |  |
|  | **W-FTC vs -5°C** |  |  |  |  |  |  |  |  | -305.75 | -489.30 | -122.20 | **0.00** |  |  |  |  |
|  | **D-FTC vs W-FTC** |  |  |  |  |  |  |  |  | -8.25 | -191.80 | 175.30 | 1.00 |  |  |  |  |
|  | **+5°C vs -5°C** |  |  |  |  |  |  |  |  | 476.50 | 292.95 | 660.05 | **0.00** |  |  |  |  |
|  |  |  |  |  |  |  |  |  |  |  |  |  |  |  |  |  |  |
| **Prokaryotic evenness** | **D-FTC vs +5°C** | -0.02 | -0.03 | -0.01 | **0.00** |  |  |  |  | 0.04 | 0.01 | 0.08 | **0.01** | -0.01 | -0.01 | 0.00 | 0.10 |
|  | **W-FTC vs +5°C** | -0.01 | -0.02 | 0.00 | 0.05 |  |  |  |  | 0.04 | 0.00 | 0.07 | **0.02** | 0.01 | 0.01 | 0.02 | **0.00** |
|  | **D-FTC vs -5°C** | -0.01 | -0.02 | 0.00 | 0.14 |  |  |  |  | 0.00 | -0.03 | 0.03 | 1.00 | -0.02 | -0.02 | -0.01 | **0.00** |
|  | **W-FTC vs -5°C** | 0.00 | -0.01 | 0.01 | 0.98 |  |  |  |  | -0.01 | -0.04 | 0.02 | 0.77 | 0.00 | 0.00 | 0.01 | 0.38 |
|  | **D-FTC vs W-FTC** | 0.01 | 0.00 | 0.02 | 0.26 |  |  |  |  | -0.01 | -0.04 | 0.02 | 0.87 | 0.02 | 0.01 | 0.03 | **0.00** |
|  | **+5°C vs -5°C** | -0.01 | -0.02 | 0.00 | 0.09 |  |  |  |  | 0.05 | 0.02 | 0.08 | **0.00** | 0.01 | 0.00 | 0.02 | **0.03** |
|  |  |  |  |  |  |  |  |  |  |  |  |  |  |  |  |  |  |
| **Prokaryotic shannon** | **D-FTC vs +5°C** | -0.15 | -0.26 | -0.03 | **0.01** |  |  |  |  | 0.40 | 0.12 | 0.69 | **0.01** | -0.05 | -0.18 | 0.09 | 0.71 |
|  | **W-FTC vs +5°C** | -0.06 | -0.17 | 0.06 | 0.50 |  |  |  |  | 0.34 | 0.05 | 0.63 | **0.02** | 0.17 | 0.04 | 0.31 | **0.01** |
|  | **D-FTC vs -5°C** | -0.06 | -0.17 | 0.06 | 0.52 |  |  |  |  | -0.12 | -0.41 | 0.17 | 0.60 | -0.18 | -0.31 | -0.04 | **0.01** |
|  | **W-FTC vs -5°C** | 0.03 | -0.08 | 0.15 | 0.82 |  |  |  |  | -0.19 | -0.47 | 0.10 | 0.27 | 0.05 | -0.09 | 0.18 | 0.73 |
|  | **D-FTC vs W-FTC** | 0.09 | -0.03 | 0.21 | 0.16 |  |  |  |  | -0.06 | -0.35 | 0.22 | 0.91 | 0.22 | 0.09 | 0.36 | **0.00** |
|  | **+5°C vs -5°C** | -0.09 | -0.21 | 0.03 | 0.15 |  |  |  |  | 0.53 | 0.24 | 0.81 | **0.00** | 0.13 | -0.01 | 0.26 | 0.07 |
|  |  |  |  |  |  |  |  |  |  |  |  |  |  |  |  |  |  |
| **Fungal richness** | **D-FTC vs +5°C** |  |  |  |  |  |  |  |  | 55.50 | -42.42 | 153.42 | 0.37 |  |  |  |  |
|  | **W-FTC vs +5°C** |  |  |  |  |  |  |  |  | 64.50 | -33.42 | 162.42 | 0.26 |  |  |  |  |
|  | **D-FTC vs -5°C** |  |  |  |  |  |  |  |  | -70.75 | -168.67 | 27.17 | 0.19 |  |  |  |  |
|  | **W-FTC vs -5°C** |  |  |  |  |  |  |  |  | -61.75 | -159.67 | 36.17 | 0.29 |  |  |  |  |
|  | **D-FTC vs W-FTC** |  |  |  |  |  |  |  |  | 9.00 | -88.92 | 106.92 | 0.99 |  |  |  |  |
|  | **+5°C vs -5°C** |  |  |  |  |  |  |  |  | 126.25 | 28.33 | 224.17 | **0.01** |  |  |  |  |

^1^diff: difference in observed means

^2^lwr: lower end point in the interval

^3^upr: upper end point in the interval

^4^Labile OC: water-extractable organic carbon

^5^Labile N: water-extractable total nitrogen

Arctic: arctic soils, Alps: alpine soils, -N: north-exposed, -S: south-exposedD-FTC: daily freeze-thaw cycles, W-FTC: weekly freeze-thaw cycles, +5°C: controls +5°C, -5°C: controls -5°C

Numbers in bold indicate p-values < 0.05

**Table S4.** Microbial activities in the soils at the end of the incubation for each FTCs treatment. Mean ± SD (n=4)

|  | **Treatment** | **Respiration^1^** | | | **GLS^2^** | | | **XYL** | | | **AP** | | | **NAG** | | | **LAP** | | | **PP^1^** | | |
| --- | --- | --- | --- | --- | --- | --- | --- | --- | --- | --- | --- | --- | --- | --- | --- | --- | --- | --- | --- | --- | --- | --- |
| **Arctic-N** | D-FTC | 4.11 | ± | 1.12 | 72.43 | ± | 36.64 | 11.54 | ± | 8.32 | 204.3 | ± | 135.11 | 19.21 | ± | 13.68 | 17.62 | ± | 17.1 | 1.21 | ± | 0.99 |
|  | W-FTC | 2.59 | ± | 7.27 | 189.17 | ± | 145.34 | 28.19 | ± | 29.49 | 232.25 | ± | 235.43 | 32.25 | ± | 30.18 | 212.57 | ± | 341.93 | 0.18 | ± | 0.37 |
|  | +5◦C | 8.22 | ± | 0.50 | 92.49 | ± | 46.44 | 12.45 | ± | 4.67 | 194.26 | ± | 73.5 | 22.25 | ± | 11.3 | 13.25 | ± | 22.43 | 1.22 | ± | 1.21 |
| **Arctic-S** | D-FTC | 13.46 | ± | 0.77 | 273.7 | ± | 167.9 | 48.78 | ± | 49.06 | 304.84 | ± | 199.91 | 51.26 | ± | 52.76 | 101.08 | ± | 160.38 | 1.64 | ± | 2.1 |
|  | W-FTC | 8.80 | ± | 0.52 | 274.1 | ± | 80 | 69.89 | ± | 25.16 | 471.05 | ± | 159.69 | 76.36 | ± | 30.63 | 0 | ± | 0 | 1.71 | ± | 1.96 |
|  | +5◦C | 17.47 | ± | 12.41 | 236.27 | ± | 174.45 | 65.79 | ± | 58.56 | 274.46 | ± | 146.53 | 29.6 | ± | 21.74 | 36.3 | ± | 40.11 | 0.87 | ± | 0.68 |
|  |  |  |  |  |  |  |  |  |  |  |  |  |  |  |  |  |  |  |  |  |  |  |
| **Alps-N** | D-FTC | 1.71 | ± | 0.34 | 92.85 | ± | 93.97 | 15.82 | ± | 22.97 | 144.35 | ± | 172.33 | 17.19 | ± | 24.8 | 105.42 | ± | 132.03 | 0.75 | ± | 0.6 |
|  | W-FTC | 0.80 | ± | 0.30 | 74.6 | ± | 54.91 | 13.11 | ± | 15.4 | 138.71 | ± | 126.21 | 16.68 | ± | 19.3 | 149 | ± | 206.9 | 0.45 | ± | 0.36 |
|  | +5◦C | 4.35 | ± | 0.58 | 32.98 | ± | 12.37 | 0.26 | ± | 0.2 | 41.51 | ± | 11.64 | 1.9 | ± | 1.42 | 7.73 | ± | 1.78 | 0.15 | ± | 0.21 |
| **Alps-S** | D-FTC | 2.40 | ± | 0.81 | 96.99 | ± | 50.79 | 8.49 | ± | 11.32 | 91.7 | ± | 68.16 | 8.25 | ± | 10.72 | 72.12 | ± | 133.14 | 0.6 | ± | 0.73 |
|  | W-FTC | 1.46 | ± | 0.13 | 54.45 | ± | 23.12 | 2.5 | ± | 2.63 | 56.57 | ± | 10.76 | 2.57 | ± | 2.1 | 20.08 | ± | 32.2 | 0.29 | ± | 0.35 |
|  | +5◦C | 7.46 | ± | 0.62 | 61 | ± | 61.66 | 14.09 | ± | 25.35 | 126.67 | ± | 150.68 | 9.26 | ± | 15.72 | 110.66 | ± | 219.53 | 0.3 | ± | 0.35 |

^1^Respiration is measured in nmol h^-1^ g DW

^2^GLS, XYL, AP, NAG and LAP are measured in µmol h^-1^ g DW, PP is measured in nmol h^-1^ g DW. GLS: β-glucosidases, XYL: β-xylosidases, AP: Acid phosphatases, NAG: N-acetyl-glucosaminidases, LAP: Leucine aminopeptidases, PP: Phenol peroxidases

Arctic: arctic soils, Alps: alpine soils, -N: north-exposed, -S: south-exposed

D-FTC: daily freeze-thaw cycles, W-FTC: weekly freeze-thaw cycles, +5°C: controls +5°C, -5°C: controls -5°C

**Table S5.** Most common prokaryotes and fungal phyla across the treatments and soils of different origin at the end of the incubation. Prokaryotes phyla correspond to > 1% of the total prokaryotic reads.

|  | % reads^1^ | # OTUs |
| --- | --- | --- |
| **PROKARYOTES** |  |  |
| Proteobacteria | 21 | 2674 |
| Chloroflexi | 20 | 1203 |
| Acidobacteria | 17 | 892 |
| Verrucomicrobia | 12 | 800 |
| Planctomycetes | 10 | 1353 |
| Actinobacteria | 6 | 526 |
| Bacteroidetes | 4 | 614 |
| Gemmatimonadetes | 2 | 152 |
| WD272 | 1.5 | 93 |
| Parcubacteria | 1 | 739 |
|  |  |  |
| **FUNGI** |  |  |
| Ascomycota | 56 | 1790 |
| Basidiomycota | 14 | 832 |
| Zygomycota | 8 | 76 |

^1^% reads: number of reads assigned to the phyla divided by the total of prokaryotic reads (2 965 563) or fungal (2 110 838) reads, respectively

**Table S6.** Prokaryotes and fungal phyla indicators associated with daily freeze-thaw cycles or weekly freeze-thaw cycles. ANOVAs were conducted to test differences in indicator taxa between the daily freeze-thaw cycle (D-FTC) and weekly freeze-thaw cycle (W-FTC) treatments across the soils.

|  | **Arctic-N** | |  | **Arctic-S** | |  | **Alps-N** | |  | **Alps-S** | |  | **Total OTUs** | **Mean seq** | **sd** |  | **Lifestyle** |  |
| --- | --- | --- | --- | --- | --- | --- | --- | --- | --- | --- | --- | --- | --- | --- | --- | --- | --- | --- |
|  | **D-FTC** | **W-FTC** |  | **D-FTC** | **W-FTC** |  | **D-FTC** | **W-FTC** |  | **D-FTC** | **W-FTC** |  |  |  |  |  |  |  |
| **PROKARYOTES** |  |  |  |  |  |  |  |  |  |  |  |  |  |  |  |  |  |  |
| Planctomycetes | 18 | 20 |  | 17 | 24 |  | 17 | 18 |  | 25 | 45 |  | 184 | 12477 | 5149 |  | o |  |
| Acidobacteria | 8 | 21 |  | 24 | 48 |  | 25 | 24 |  | 9 | 22 |  | 181 | 25565 | 9422 |  | o |  |
| Verrucomicrobia | 4 | 35 |  | 16 | 29 |  | 6 | 23 |  | 2 | 33 |  | 148 | 37745 | 26545 |  | o | *** |
| Chloroflexi | 10 | 11 |  | 25 | 19 |  | 18 | 14 |  | 13 | 22 |  | 132 | 27861 | 24126 |  | o |  |
| Actinobacteria | 13 |  |  | 21 | 10 |  | 25 | 10 |  | 17 | 9 |  | 105 | 16349 | 13974 |  | c (o) | * |
| Alphaproteobacteria | 7 | 4 |  | 8 | 10 |  | 12 | 10 |  | 20 | 24 |  | 95 | 13446 | 9951 |  | c (o) |  |
| Betaproteobacteria | 4 | 6 |  | 14 | 21 |  | 16 | 13 |  | 10 | 10 |  | 94 | 10750 | 6140 |  | c |  |
| Gammaproteobacteria | 1 | 8 |  | 10 | 10 |  | 8 | 8 |  | 4 | 11 |  | 60 | 11020 | 4630 |  | c (o) |  |
| Deltaproteobacteria |  | 1 |  | 6 | 2 |  | 5 | 5 |  | 10 | 10 |  | 39 | 706 | 638 |  | o |  |
| Bacteroidetes | 2 | 6 |  | 11 | 20 |  | 2 | 1 |  |  | 14 |  | 56 | 2226 | 870 |  | c (o) |  |
| WD272 | 1 | 13 |  | 2 | 3 |  | 1 | 13 |  | 4 | 12 |  | 49 | 5522 | 2797 |  | u$ | * |
| Gemmatimonadetes |  | 1 |  | 4 | 5 |  | 5 | 4 |  | 1 | 4 |  | 24 | 1179 | 1260 |  | u |  |
| Parcubacteria | 1 | 2 |  |  | 5 |  |  | 1 |  |  | 5 |  | 14 | 60 | 49 |  | u | * |
| Armatimonadetes |  | 2 |  | 1 | 1 |  | 2 | 2 |  |  | 4 |  | 12 | 136 | 102 |  | u |  |
| Chlorobi |  | 1 |  |  |  |  |  |  |  | 1 | 6 |  | 8 | 229 | 252 |  |  |  |
| Cyanobacteria | 2 | 1 |  |  | 1 |  |  | 1 |  | 2 | 1 |  | 8 | 741 | 1438 |  |  |  |
| Nitrospirae | 1 | 1 |  | 1 | 2 |  |  | 1 |  | 1 | 1 |  | 8 | 360 | 368 |  | u |  |
| Saccharibacteria |  | 1 |  | 1 | 3 |  | 1 |  |  |  |  |  | 6 | 51 | 22 |  | u |  |
| Firmicutes |  |  |  | 1 | 1 |  |  |  |  | 1 | 2 |  | 5 | 55 | 34 |  | c (o) |  |
| TA18 | 2 | 1 |  |  |  |  |  |  |  | 1 | 1 |  | 5 | 146 | 81 |  | u$ |  |
| Microgenomates |  |  |  | 2 | 2 |  |  |  |  |  |  |  | 4 | 180 |  |  | u$ |  |
| SM2F11 |  |  |  |  | 1 |  |  |  |  | 0 | 3 |  | 4 | 432 | 586 |  | u$ |  |
| Elusimicrobia | 1 |  |  |  | 2 |  |  |  |  |  |  |  | 3 | 23 | 17 |  | u |  |
| SHA-109 |  |  |  |  |  |  |  |  |  | 1 | 1 |  | 2 | 26 |  |  | u$ |  |
| Thermotogae |  |  |  |  |  |  |  |  |  | 1 | 1 |  | 2 | 23 |  |  |  |  |
| TM6 | 1 |  |  |  | 1 |  |  |  |  |  |  |  | 2 | 18 | 19 |  | u |  |
| WCHB1-60 |  |  |  |  | 2 |  |  |  |  |  |  |  | 2 | 39 |  |  | u |  |
| Chlamydiae |  |  |  |  |  |  |  |  |  |  | 1 |  | 1 | 6 |  |  |  |  |
| Deinococcus-Thermus |  |  |  |  |  |  | 1 |  |  |  |  |  | 1 | 60 |  |  |  |  |
| Gracilibacteria |  |  |  |  | 1 |  |  |  |  |  |  |  | 1 | 3 |  |  | u$ |  |
| Hydrogenedentes |  |  |  |  | 1 |  |  |  |  |  |  |  | 1 | 19 |  |  | u$ |  |
| unclassified | 2 |  |  | 1 | 2 |  | 1 | 4 |  | 2 |  |  | 12 | 87 | 90 |  |  |  |
| **TOTAL** | **78** | **135** |  | **165** | **226** |  | **145** | **152** |  | **125** | **242** |  |  |  |  |  |  |  |
|  |  |  |  |  |  |  |  |  |  |  |  |  |  |  |  |  |  |  |
| **FUNGI** |  |  |  |  |  |  |  |  |  |  |  |  |  |  |  |  |  |  |
| Ascomycota | 5 | 5 |  | 35 | 27 |  | 7 | 12 |  | 4 | 10 |  | 105 | 13538 | 13057 |  |  |  |
| Basidiomycota | 2 | 3 |  | 7 | 8 |  | 0 | 3 |  | 2 | 1 |  | 26 | 5796 | 9804 |  |  |  |
| Zygomycota |  |  |  | 0 | 1 |  | 3 | 2 |  | 0 | 1 |  | 7 | 10154 | 13684 |  |  |  |
| unclassified | 1 | 2 |  | 7 | 6 |  | 3 | 5 |  | 3 | 2 |  | 29 | 2437 | 2577 |  |  |  |
| **TOTAL** | **8** | **10** |  | **49** | **42** |  | **13** | **22** |  | **9** | **14** |  |  |  |  |  |  |  |

Arctic: arctic soils, Alps: alpine soils, -N: north-exposed, -S: south-exposed

o: oligotrophic, c: copiotrophic, c(o): mostly copiotrophic but also association with oligotrophs, o(c): oligotrophic but also association with copiotrophs, u: unclear; taxa with insufficient information (taxa with fewer than 10 cultivated strains recorded in the NCBI database or candidate taxa) to be classified to a life strategy.

Mean seq and sd: mean number of reads across the samples classified to the specified taxa and 1 standard deviation

$ = candidate taxa

***p<0.001, * p<0.05

**Table S7.** FunGuild functional assignments for fungal FTC-indicators.

| **soil** | **otus** | **Taxon** | **Trophic.Mode** | **Guild** | **Growth.Morphology** | **Confidence.Ranking** | **D-FTC** | **W-FTC** | | **+5°C** | **-5°C** |
| --- | --- | --- | --- | --- | --- | --- | --- | --- | --- | --- | --- |
| **Alps-N** | OTUe_1006 | Phaeosphaeria | Saprotroph | Undefined Saprotroph | NULL | Probable | 1 | 0 | 0 | | 0 |
| **Alps-N** | OTUe_106 | Acarospora | Symbiotroph | Lichenized | Thallus | Highly Probable | 1 | 1 | 0 | | 1 |
| **Alps-N** | OTUe_1162 | Lecanora | Symbiotroph | Lichenized | Thallus | Highly Probable | 1 | 0 | 0 | | 0 |
| **Alps-N** | OTUe_16 | Mortierellaceae | Saprotroph-Symbiotroph | Endophyte-Litter Saprotroph-Soil Saprotroph-Undefined Saprotroph | Microfungus | Possible | 0 | 1 | 0 | | 1 |
| **Alps-N** | OTUe_1930 | Lecidea | Symbiotroph | Lichenized | Thallus | Highly Probable | 0 | 1 | 0 | | 0 |
| **Alps-N** | OTUe_2000 | Hypocreales | Saprotroph | Undefined Saprotroph | Microfungus | Possible | 1 | 1 | 0 | | 0 |
| **Alps-N** | OTUe_2157 | Mortierellaceae | Saprotroph-Symbiotroph | Endophyte-Litter Saprotroph-Soil Saprotroph-Undefined Saprotroph | Microfungus | Possible | 1 | 0 | 0 | | 0 |
| **Alps-N** | OTUe_3013 | Hyaloscypha | Saprotroph | Undefined Saprotroph | NULL | Probable | 0 | 1 | 0 | | 0 |
| **Alps-N** | OTUe_302 | Mortierellaceae | Saprotroph-Symbiotroph | Endophyte-Litter Saprotroph-Soil Saprotroph-Undefined Saprotroph | Microfungus | Possible | 1 | 0 | 0 | | 1 |
| **Alps-N** | OTUe_39 | Mortierellaceae | Saprotroph-Symbiotroph | Endophyte-Litter Saprotroph-Soil Saprotroph-Undefined Saprotroph | Microfungus | Possible | 1 | 1 | 0 | | 1 |
| **Alps-N** | OTUe_48 | Cladosporium | Pathotroph-Saprotroph-Symbiotroph | Animal Pathogen-Endophyte-Lichen Parasite-Plant Pathogen-Wood Saprotroph | Microfungus | Possible | 1 | 0 | 0 | | 0 |
| **Alps-N** | OTUe_610 | Tremellales | Pathotroph-Saprotroph-Symbiotroph | Fungal Parasite-Undefined Saprotroph | Tremelloid-Yeast | Possible | 0 | 1 | 0 | | 0 |
| **Alps-N** | OTUe_759 | Tremella | Pathotroph | Fungal Parasite-Lichen Parasite | Facultative Yeast-Microfungus-Tremelloid | Probable | 0 | 1 | 0 | | 0 |
| **Alps-N** | OTUe_768 | Acarospora | Symbiotroph | Lichenized | Thallus | Highly Probable | 1 | 1 | 0 | | 1 |
| **Alps-S** | OTUe_2615 | Geomyces | Saprotroph | Soil Saprotroph | Microfungus | Probable | 1 | 0 | 0 | | 1 |
| **Alps-S** | OTUe_2633 | Geomyces | Saprotroph | Soil Saprotroph | Microfungus | Probable | 1 | 0 | 0 | | 1 |
| **Alps-S** | OTUe_2979 | Tetracladium | Saprotroph | Undefined Saprotroph | NULL | Probable | 1 | 0 | 0 | | 0 |
| **Alps-S** | OTUe_420 | Sporormiaceae | Saprotroph | Dung Saprotroph-Plant Saprotroph | Microfungus | Probable | 0 | 1 | 0 | | 0 |
| **Arctic-N** | OTUe_24 | Tomentella | Symbiotroph | Ectomycorrhizal | Corticioid | Highly Probable | 0 | 1 | 0 | | 0 |
| **Arctic-N** | OTUe_3640 | Cladophialophora | Saprotroph | Undefined Saprotroph | Facultative Yeast | Probable | 1 | 0 | 0 | | 0 |
| **Arctic-N** | OTUe_3727 | Eurotiales | Saprotroph | Undefined Saprotroph | Microfungus | Possible | 1 | 0 | 0 | | 1 |
| **Arctic-N** | OTUe_541 | Thelephoraceae | Saprotroph-Symbiotroph | Ectomycorrhizal-Undefined Saprotroph | Clavarioid | Probable | 0 | 1 | 0 | | 0 |
| **Arctic-N** | OTUe_808 | Clavulina | Symbiotroph | Ectomycorrhizal | Clavarioid | Highly Probable | 0 | 1 | 0 | | 0 |
| **Arctic-S** | OTUe_1077 | Capronia | Symbiotroph | Endophyte | Facultative Yeast | Highly Probable | 1 | 0 | 0 | | 0 |
| **Arctic-S** | OTUe_1187 | Ramariopsis | Saprotroph | Undefined Saprotroph | Clavarioid | Probable | 0 | 1 | 0 | | 0 |
| **Arctic-S** | OTUe_1189 | Devriesia | Pathotroph | Plant Pathogen | NULL | Probable | 1 | 0 | 0 | | 0 |
| **Arctic-S** | OTUe_154 | Cadophora | Symbiotroph | Endophyte | Microfungus | Highly Probable | 1 | 1 | 0 | | 0 |
| **Arctic-S** | OTUe_1736 | Cladophialophora | Saprotroph | Undefined Saprotroph | Facultative Yeast | Probable | 1 | 1 | 0 | | 0 |
| **Arctic-S** | OTUe_1872 | Porpidia | Symbiotroph | Lichenized | Thallus | Highly Probable | 1 | 0 | 0 | | 0 |
| **Arctic-S** | OTUe_2 | Geomyces | Saprotroph | Soil Saprotroph | Microfungus | Probable | 1 | 1 | 0 | | 0 |
| **Arctic-S** | OTUe_212 | Inocybaceae | Symbiotroph | Ectomycorrhizal | Agaricoid | Probable | 0 | 1 | 0 | | 0 |
| **Arctic-S** | OTUe_2179 | Clavariaceae | Saprotroph-Symbiotroph | Lichenized-Undefined Saprotroph | Clavarioid | Probable | 1 | 1 | 0 | | 1 |
| **Arctic-S** | OTUe_2353 | Eurotiales | Saprotroph | Undefined Saprotroph | Microfungus | Possible | 1 | 1 | 0 | | 0 |
| **Arctic-S** | OTUe_2498 | Mortierellaceae | Saprotroph-Symbiotroph | Endophyte-Litter Saprotroph-Soil Saprotroph-Undefined Saprotroph | Microfungus | Possible | 0 | 1 | 0 | | 0 |
| **Arctic-S** | OTUe_2735 | Tetracladium | Saprotroph | Undefined Saprotroph | NULL | Probable | 1 | 0 | 0 | | 0 |
| **Arctic-S** | OTUe_2871 | Herpotrichiellaceae | Pathotroph-Saprotroph | Animal Pathogen-Fungal Parasite-Undefined Saprotroph | Facultative Yeast-Microfungus | Probable | 1 | 0 | 0 | | 0 |
| **Arctic-S** | OTUe_2974 | Herpotrichiellaceae | Pathotroph-Saprotroph | Animal Pathogen-Fungal Parasite-Undefined Saprotroph | Facultative Yeast-Microfungus | Probable | 1 | 0 | 0 | | 0 |
| **Arctic-S** | OTUe_3179 | Sebacina | Symbiotroph | Ectomycorrhizal-Orchid Mycorrhizal-Root Associated Biotroph | NULL | Possible | 1 | 1 | 0 | | 0 |
| **Arctic-S** | OTUe_32 | Hygrocybe | Saprotroph-Symbiotroph | Undefined Saprotroph-Undefined Biotroph | Agaricoid | Probable | 1 | 1 | 0 | | 0 |
| **Arctic-S** | OTUe_342 | Clavaria | Saprotroph | Undefined Saprotroph | Clavarioid | Probable | 0 | 1 | 0 | | 0 |
| **Arctic-S** | OTUe_473 | Sebacina | Symbiotroph | Ectomycorrhizal-Orchid Mycorrhizal-Root Associated Biotroph | NULL | Possible | 1 | 1 | 0 | | 0 |
| **Arctic-S** | OTUe_716 | Stilbella | Saprotroph-Symbiotroph | Dung Saprotroph-Endophyte-Wood Saprotroph | NULL | Possible | 1 | 1 | 0 | | 0 |
| **Arctic-S** | OTUe_76 | Cladosporium | Pathotroph-Saprotroph-Symbiotroph | Animal Pathogen-Endophyte-Lichen Parasite-Plant Pathogen-Wood Saprotroph | Microfungus | Possible | 1 | 0 | 0 | | 0 |
| **Arctic-S** | OTUe_910 | Ramariopsis | Saprotroph | Undefined Saprotroph | Clavarioid | Probable | 1 | 0 | 0 | | 0 |
| **Arctic-S** | OTUe_939 | Clavaria | Saprotroph | Undefined Saprotroph | Clavarioid | Probable | 1 | 1 | 0 | | 0 |

1: significantly associated (p < 0.05), 0: non-significantly associated.

pink: associated with the daily freeze-thaw cycles (D-FTC), yellow: associated with the weekly freeze-thaw cycles (W-FTC), blue: associated with both FTC treatments

Arctic: arctic soils, Alps: alpine soils, -N: north-exposed, -S: south-exposed

+5°C: controls +5°C, -5°C: controls -5°C

**Table S8.** Indicator species analyses for Prokaryotes

**Table S9.** Indicator species analyses for Fungi

These two tables are provided separately because of their large extension.
