# Supplementary material for: Functional and Structural Responses of Arctic and Alpine Soil Prokaryotic and Fungal Communities Under Freeze-Thaw Cycles of Different Frequencies
Source: Front Microbiol. 2020 May 25;11:982. doi: 10.3389/fmicb.2020.00982 (PMC7261861; doi:10.3389/fmicb.2020.00982)
Supplement: Supplementary file 8 [file Data_Sheet_2.zip › Supplementary Table 8.PDF]

Soil	Otus	Kingdom	Phylum	Class	Order	Family	Genus	Species	ctrl +5C	ctrl -5°C	D-FTC	W-FTC	s.ctrl +5°C	ctrl -5°C	s.D-FTC	s.W-FTC	stat	*p*
**Arctic-N**	OTUp_4	Bacteria	Actinobacteria	Actinobacteria	Propionibacteriales	Nocardioidaceae	Nocardioides	unclassified	0.005	-0.384	0.665	-0.285	0	0	1	0	0.66	0.048
**Arctic-N**	OTUp_7	Bacteria	Proteobacteria	Alphaproteobacteria	Rhizobiales	Beijerinckiaceae	uncultured	uncultured_bacterium	-0.126	0.534	0.253	-0.661	0	1	1	0	0.68	0.030
**Arctic-N**	OTUp_11	Bacteria	Verrucomicrobia	Spartobacteria	Chthoniobacterales	DA101_soil_group	uncultured_soil_bacterium	unclassified	-0.333	-0.275	-0.289	0.897	0	0	0	1	0.90	0.002
**Arctic-N**	OTUp_12	Bacteria	Verrucomicrobia	Spartobacteria	Chthoniobacterales	DA101_soil_group	uncultured_Verrucomicrobia_bacterium	unclassified	-0.385	-0.062	-0.352	0.799	0	0	0	1	0.80	0.004
**Arctic-N**	OTUp_14	Bacteria	Acidobacteria	Acidobacteria	Subgroup_2	uncultured_Acidobacteria_bacterium	unclassified	unclassified	-0.253	-0.546	0.033	0.766	0	0	0	1	0.77	0.002
**Arctic-N**	OTUp_15	Bacteria	Proteobacteria	Betaproteobacteria	SC-I-84	uncultured_beta_proteobacterium	unclassified	unclassified	-0.310	-0.472	-0.049	0.832	0	0	0	1	0.83	0.004
**Arctic-N**	OTUp_18	Bacteria	Acidobacteria	Acidobacteria	Subgroup_3	Unknown_Family	Candidatus_Solibacter	uncultured_bacterium	-0.282	-0.490	0.227	0.546	0	0	1	1	0.67	0.026
**Arctic-N**	OTUp_20	Bacteria	Verrucomicrobia	Spartobacteria	Chthoniobacterales	DA101_soil_group	uncultured_Verrucomicrobia_bacterium	unclassified	-0.441	-0.168	-0.320	0.929	0	0	0	1	0.93	0.002
**Arctic-N**	OTUp_21	Bacteria	Planctomycetes	Planctomycetacia	Planctomycetales	Planctomycetaceae	uncultured	uncultured_Zavarzinella_sp.	-0.632	0.519	-0.037	0.149	0	1	1	1	0.63	0.039
**Arctic-N**	OTUp_22	Bacteria	Acidobacteria	Acidobacteria	Subgroup_4	RB41	uncultured_Acidobacteria_bacterium	unclassified	-0.284	-0.174	-0.348	0.807	0	0	0	1	0.81	0.001
**Arctic-N**	OTUp_23	Bacteria	Acidobacteria	Acidobacteria	Acidobacteriales	Acidobacteriaceae	uncultured	uncultured_bacterium	-0.187	-0.117	-0.499	0.803	0	0	0	1	0.80	0.001
**Arctic-N**	OTUp_30	Bacteria	Verrucomicrobia	Spartobacteria	Chthoniobacterales	Xiphinematobacteraceae	Candidatus_Xiphinematobacter	uncultured_bacterium	-0.365	0.461	0.309	-0.405	0	1	1	0	0.67	0.027
**Arctic-N**	OTUp_31	Bacteria	Actinobacteria	Actinobacteria	Micrococcales	Micrococcaceae	Arthrobacter	unclassified	-0.080	-0.382	0.841	-0.379	0	0	1	0	0.84	0.005
**Arctic-N**	OTUp_34	Bacteria	Proteobacteria	Gammaproteobacteria	Xanthomonadales	Xanthomonadaceae	Rhodanobacter	uncultured_Xanthomonadaceae_bacterium	-0.245	-0.273	-0.277	0.795	0	0	0	1	0.80	0.004
**Arctic-N**	OTUp_36	Bacteria	Verrucomicrobia	Spartobacteria	Chthoniobacterales	DA101_soil_group	uncultured_Prosthecobacter_sp.	unclassified	-0.241	-0.218	-0.250	0.710	0	0	0	1	0.71	0.017
**Arctic-N**	OTUp_46	Bacteria	WD272	uncultured_Firmicutes_bacterium	unclassified	unclassified	unclassified	unclassified	-0.261	-0.296	-0.325	0.882	0	0	0	1	0.88	0.001
**Arctic-N**	OTUp_48	Bacteria	Verrucomicrobia	Spartobacteria	Chthoniobacterales	DA101_soil_group	uncultured_bacterium	unclassified	-0.428	0.057	-0.401	0.772	0	0	0	1	0.77	0.004
**Arctic-N**	OTUp_50	Bacteria	WD272	unclassified	unclassified	unclassified	unclassified	unclassified	-0.353	-0.222	-0.336	0.911	0	0	0	1	0.91	0.001
**Arctic-N**	OTUp_54	Bacteria	Acidobacteria	Acidobacteria	Subgroup_3	Unknown_Family	Bryobacter	uncultured_Holophaga_sp.	-0.342	-0.324	0.003	0.663	0	0	0	1	0.66	0.037
**Arctic-N**	OTUp_56	Bacteria	Chloroflexi	S085	uncultured_bacterium	unclassified	unclassified	unclassified	-0.416	-0.153	-0.061	0.630	0	0	0	1	0.63	0.050
**Arctic-N**	OTUp_60	Bacteria	Actinobacteria	Actinobacteria	Frankiales	Acidothermaceae	Acidothermus	uncultured_Actinoallomurus_sp.	-0.113	0.229	0.536	-0.652	0	1	1	0	0.66	0.034
**Arctic-N**	OTUp_61	Bacteria	Verrucomicrobia	Spartobacteria	Chthoniobacterales	DA101_soil_group	uncultured_Spartobacteria_bacterium	unclassified	-0.418	-0.132	-0.234	0.784	0	0	0	1	0.78	0.001
**Arctic-N**	OTUp_63	Bacteria	Acidobacteria	Acidobacteria	Subgroup_3	Unknown_Family	Bryobacter	uncultured_bacterium	-0.368	0.286	0.457	-0.375	0	1	1	0	0.64	0.044
**Arctic-N**	OTUp_65	Bacteria	Proteobacteria	Deltaproteobacteria	Myxococcales	BIrii41	uncultured_bacterium	unclassified	-0.159	-0.376	-0.218	0.754	0	0	0	1	0.75	0.008
**Arctic-N**	OTUp_66	Bacteria	Chloroflexi	Ktedonobacteria	Ktedonobacterales	unclassified	unclassified	unclassified	-0.064	-0.342	0.635	-0.229	0	0	1	0	0.63	0.005
**Arctic-N**	OTUp_73	Bacteria	WD272	uncultured_bacterium	unclassified	unclassified	unclassified	unclassified	-0.172	-0.223	-0.427	0.822	0	0	0	1	0.82	0.002
**Arctic-N**	OTUp_74	Bacteria	Cyanobacteria	Chloroplast	unclassified	unclassified	unclassified	unclassified	-0.150	-0.232	0.621	-0.239	0	0	1	0	0.62	0.038
**Arctic-N**	OTUp_75	Bacteria	Acidobacteria	Holophagae	Subgroup_7	uncultured_proteobacterium	unclassified	unclassified	-0.117	-0.703	0.323	0.497	0	0	1	1	0.71	0.010
**Arctic-N**	OTUp_83	Bacteria	WD272	uncultured_bacterium	unclassified	unclassified	unclassified	unclassified	-0.082	-0.489	-0.230	0.801	0	0	0	1	0.80	0.006
**Arctic-N**	OTUp_90	Bacteria	Planctomycetes	Planctomycetacia	Planctomycetales	Planctomycetaceae	uncultured	uncultured_bacterium	-0.664	0.347	0.197	0.121	0	1	1	1	0.66	0.043
**Arctic-N**	OTUp_95	Bacteria	Chloroflexi	Ktedonobacteria	Ktedonobacterales	JG30a-KF-32	unclassified	unclassified	-0.693	0.345	-0.034	0.382	0	1	1	1	0.69	0.012
**Arctic-N**	OTUp_103	Bacteria	Bacteroidetes	Sphingobacteriia	Sphingobacteriales	unclassified	unclassified	unclassified	-0.329	0.364	-0.503	0.468	0	1	0	1	0.72	0.011
**Arctic-N**	OTUp_104	Bacteria	Verrucomicrobia	Spartobacteria	Chthoniobacterales	DA101_soil_group	uncultured_bacterium	unclassified	-0.298	-0.064	-0.416	0.778	0	0	0	1	0.78	0.005
**Arctic-N**	OTUp_110	Bacteria	Planctomycetes	Planctomycetacia	Planctomycetales	Planctomycetaceae	uncultured	uncultured_bacterium	-0.533	0.484	0.277	-0.228	0	1	1	0	0.66	0.041
**Arctic-N**	OTUp_117	Bacteria	Acidobacteria	Acidobacteria	Acidobacteriales	Acidobacteriaceae	unclassified	unclassified	-0.492	-0.196	-0.120	0.809	0	0	0	1	0.81	0.005
**Arctic-N**	OTUp_121	Bacteria	Chloroflexi	JG37-AG-4	uncultured_Chloroflexi_bacterium	unclassified	unclassified	unclassified	-0.142	0.310	0.499	-0.667	0	1	1	0	0.70	0.020
**Arctic-N**	OTUp_123	Bacteria	Proteobacteria	Alphaproteobacteria	Rhizobiales	Xanthobacteraceae	Variibacter	uncultured_bacterium	-0.042	0.001	-0.600	0.642	0	0	0	1	0.64	0.047
**Arctic-N**	OTUp_126	Bacteria	Acidobacteria	Acidobacteria	Subgroup_2	uncultured_forest_soil_bacterium	unclassified	unclassified	-0.171	-0.366	-0.169	0.706	0	0	0	1	0.71	0.018
**Arctic-N**	OTUp_127	Bacteria	Acidobacteria	Holophagae	Subgroup_7	uncultured_proteobacterium	unclassified	unclassified	-0.251	-0.503	0.187	0.567	0	0	1	1	0.65	0.047
**Arctic-N**	OTUp_138	Bacteria	Proteobacteria	Gammaproteobacteria	Xanthomonadales	Xanthomonadaceae	unclassified	unclassified	-0.025	-0.401	-0.427	0.853	0	0	0	1	0.85	0.002
**Arctic-N**	OTUp_147	Bacteria	Proteobacteria	Alphaproteobacteria	Rhizobiales	Rhodobiaceae	uncultured	uncultured_Rhizobiales_bacterium	-0.123	0.450	0.380	-0.707	0	1	1	0	0.72	0.011
**Arctic-N**	OTUp_153	Bacteria	Planctomycetes	Phycisphaerae	WD2101_soil_group	uncultured_bacterium	unclassified	unclassified	-0.326	0.109	-0.429	0.646	0	1	0	1	0.65	0.044
**Arctic-N**	OTUp_154	Bacteria	Actinobacteria	Acidimicrobiia	Acidimicrobiales	uncultured	unclassified	unclassified	0.044	-0.200	0.643	-0.487	0	0	1	0	0.64	0.008
**Arctic-N**	OTUp_164	Bacteria	Proteobacteria	Betaproteobacteria	Burkholderiales	Burkholderiaceae	Burkholderia	Burkholderia_sordidicola	0.049	-0.424	0.784	-0.409	0	0	1	0	0.78	0.005
**Arctic-N**	OTUp_199	Bacteria	Planctomycetes	Planctomycetacia	Planctomycetales	Planctomycetaceae	uncultured	uncultured_Planctomycetia_bacterium	-0.567	0.429	-0.275	0.413	0	1	0	1	0.73	0.014
**Arctic-N**	OTUp_213	Bacteria	Nitrospirae	Nitrospira	Nitrospirales	0319-6A21	uncultured_candidate_division_SPAM_bacterium	unclassified	-0.492	-0.287	0.779	0.001	0	0	1	0	0.78	0.002
**Arctic-N**	OTUp_214	Bacteria	Actinobacteria	Thermoleophilia	Solirubrobacterales	TM146	uncultured_Conexibacteraceae_bacterium	unclassified	-0.143	0.468	0.316	-0.640	0	1	1	0	0.68	0.016
**Arctic-N**	OTUp_225	Bacteria	Proteobacteria	Alphaproteobacteria	Rhizobiales	unclassified	unclassified	unclassified	-0.483	0.565	0.279	-0.361	0	1	1	0	0.73	0.007
**Arctic-N**	OTUp_227	Bacteria	WD272	uncultured_bacterium	unclassified	unclassified	unclassified	unclassified	-0.128	-0.340	-0.286	0.754	0	0	0	1	0.75	0.009
**Arctic-N**	OTUp_229	Bacteria	Planctomycetes	Planctomycetacia	Planctomycetales	Planctomycetaceae	uncultured	uncultured_planctomycete	-0.378	-0.026	-0.260	0.665	0	0	0	1	0.66	0.034
**Arctic-N**	OTUp_239	Bacteria	Nitrospirae	Nitrospira	Nitrospirales	Nitrospiraceae	Nitrospira	uncultured_Nitrospira_sp.	-0.253	-0.292	-0.094	0.639	0	0	0	1	0.64	0.044
**Arctic-N**	OTUp_243	Bacteria	Bacteroidetes	Cytophagia	Cytophagales	Cytophagaceae	Cytophaga	uncultured_bacterium	-0.639	0.465	-0.185	0.359	0	1	0	1	0.71	0.014
**Arctic-N**	OTUp_249	Bacteria	Acidobacteria	Acidobacteria	Subgroup_3	Unknown_Family	Candidatus_Solibacter	unclassified	-0.254	-0.133	-0.300	0.687	0	0	0	1	0.69	0.024
**Arctic-N**	OTUp_251	Bacteria	WD272	uncultured_bacterium	unclassified	unclassified	unclassified	unclassified	-0.166	-0.300	-0.449	0.916	0	0	0	1	0.92	0.002
**Arctic-N**	OTUp_259	Bacteria	Bacteroidetes	Sphingobacteriia	Sphingobacteriales	Chitinophagaceae	uncultured	uncultured_soil_bacterium	-0.103	-0.533	-0.054	0.691	0	0	0	1	0.69	0.020
**Arctic-N**	OTUp_275	Bacteria	Planctomycetes	Phycisphaerae	WD2101_soil_group	uncultured_bacterium	unclassified	unclassified	-0.129	-0.487	-0.047	0.663	0	0	0	1	0.66	0.029
**Arctic-N**	OTUp_283	Bacteria	Proteobacteria	Gammaproteobacteria	Xanthomonadales	Xanthomonadaceae	Rhodanobacter	uncultured_Xanthomonadaceae_bacterium	-0.155	-0.467	-0.014	0.636	0	0	0	1	0.64	0.046
**Arctic-N**	OTUp_287	Bacteria	Acidobacteria	Acidobacteria	Subgroup_3	Unknown_Family	Candidatus_Solibacter	uncultured_bacterium	-0.102	-0.192	-0.351	0.645	0	0	0	1	0.64	0.034
**Arctic-N**	OTUp_293	Bacteria	Chloroflexi	Ktedonobacteria	Ktedonobacterales	JG30a-KF-32	uncultured_Ktedobacteria_bacterium	unclassified	-0.511	0.417	0.378	-0.284	0	1	1	0	0.69	0.020
**Arctic-N**	OTUp_309	Bacteria	Actinobacteria	Actinobacteria	Frankiales	Acidothermaceae	Acidothermus	uncultured_Thermomonosporaceae_bacterium	-0.442	0.443	0.548	-0.549	0	1	1	0	0.86	0.000
**Arctic-N**	OTUp_350	Bacteria	WD272	uncultured_bacterium	unclassified	unclassified	unclassified	unclassified	-0.257	-0.438	-0.059	0.753	0	0	0	1	0.75	0.012
**Arctic-N**	OTUp_374	Bacteria	Proteobacteria	Alphaproteobacteria	Rhizobiales	Rhizobiales_Incertae_Sedis	Rhizomicrobium	uncultured_proteobacterium	-0.519	-0.333	0.035	0.817	0	0	0	1	0.82	0.005
**Arctic-N**	OTUp_384	Bacteria	WD272	uncultured_Firmicutes_bacterium	unclassified	unclassified	unclassified	unclassified	-0.384	-0.135	-0.320	0.840	0	0	0	1	0.84	0.004
**Arctic-N**	OTUp_385	Bacteria	Verrucomicrobia	Spartobacteria	Chthoniobacterales	DA101_soil_group	uncultured_Verrucomicrobia_bacterium	unclassified	-0.555	0.337	-0.346	0.564	0	1	0	1	0.78	0.005
**Arctic-N**	OTUp_387	Bacteria	Proteobacteria	Betaproteobacteria	TRA3-20	unclassified	unclassified	unclassified	-0.468	-0.319	0.401	0.387	0	0	1	1	0.68	0.028
**Arctic-N**	OTUp_416	Bacteria	Planctomycetes	Planctomycetacia	Planctomycetales	Planctomycetaceae	uncultured	unclassified	-0.727	0.275	-0.060	0.512	0	1	1	1	0.73	0.013
**Arctic-N**	OTUp_422	Bacteria	Verrucomicrobia	Spartobacteria	Chthoniobacterales	Chthoniobacteraceae	Chthoniobacter	bacterium_Ellin506	-0.195	-0.331	-0.110	0.637	0	0	0	1	0.64	0.048
**Arctic-N**	OTUp_449	Bacteria	Proteobacteria	Gammaproteobacteria	Cellvibrionales	Cellvibrionaceae	Cellvibrio	unclassified	-0.554	0.338	-0.332	0.548	0	1	0	1	0.77	0.010
**Arctic-N**	OTUp_484	Bacteria	Actinobacteria	Actinobacteria	Micrococcales	Micrococcaceae	Arthrobacter	unclassified	0.102	-0.395	0.751	-0.458	0	0	1	0	0.75	0.010
**Arctic-N**	OTUp_486	Bacteria	Verrucomicrobia	OPB35_soil_group	unclassified	unclassified	unclassified	unclassified	-0.371	0.323	-0.389	0.438	0	1	0	1	0.66	0.044
**Arctic-N**	OTUp_510	Bacteria	Proteobacteria	Alphaproteobacteria	Rhodospirillales	Rhodospirillales_Incertae_Sedis	Reyranella	uncultured_bacterium	-0.442	-0.329	0.271	0.500	0	0	1	1	0.67	0.037
**Arctic-N**	OTUp_547	Bacteria	Actinobacteria	Acidimicrobiia	Acidimicrobiales	uncultured	uncultured_Ferrimicrobium_sp.	unclassified	-0.358	0.307	0.518	-0.468	0	1	1	0	0.71	0.018
**Arctic-N**	OTUp_552	Bacteria	Verrucomicrobia	Spartobacteria	Chthoniobacterales	01D2Z36	uncultured_cyanobacterium	unclassified	-0.007	-0.559	-0.236	0.803	0	0	0	1	0.80	0.001
**Arctic-N**	OTUp_577	Bacteria	Cyanobacteria	Melainabacteria	Obscuribacterales	uncultured_cyanobacterium	unclassified	unclassified	-0.207	-0.128	-0.487	0.822	0	0	0	1	0.82	0.002
**Arctic-N**	OTUp_581	Bacteria	Planctomycetes	Planctomycetacia	Planctomycetales	Planctomycetaceae	Singulisphaera	uncultured_bacterium	-0.398	0.691	0.262	-0.555	0	1	1	0	0.83	0.002
**Arctic-N**	OTUp_646	Bacteria	Acidobacteria	Acidobacteria	Acidobacteriales	Acidobacteriaceae	Candidatus_Koribacter	uncultured_Acidobacteria_bacterium	-0.241	-0.423	0.000	0.663	0	0	0	1	0.66	0.038
**Arctic-N**	OTUp_661	Bacteria	Chloroflexi	Anaerolineae	Anaerolineales	Anaerolineaceae	uncultured	uncultured_Gemmatimonadetes_bacterium	-0.225	-0.475	0.060	0.639	0	0	0	1	0.64	0.049
**Arctic-N**	OTUp_677	Bacteria	Verrucomicrobia	Spartobacteria	Chthoniobacterales	DA101_soil_group	uncultured_Verrucomicrobia_bacterium	unclassified	-0.198	-0.233	-0.364	0.795	0	0	0	1	0.79	0.005
**Arctic-N**	OTUp_695	Bacteria	Verrucomicrobia	Spartobacteria	Chthoniobacterales	DA101_soil_group	unclassified	unclassified	-0.306	-0.333	-0.097	0.737	0	0	0	1	0.74	0.006
**Arctic-N**	OTUp_704	Bacteria	Planctomycetes	Phycisphaerae	WD2101_soil_group	uncultured_bacterium	unclassified	unclassified	-0.303	-0.093	-0.270	0.666	0	0	0	1	0.67	0.036
**Arctic-N**	OTUp_720	Bacteria	Verrucomicrobia	Spartobacteria	Chthoniobacterales	DA101_soil_group	uncultured_Verrucomicrobia_bacterium	unclassified	-0.376	-0.349	-0.155	0.880	0	0	0	1	0.88	0.001
**Arctic-N**	OTUp_727	Bacteria	Chloroflexi	unclassified	unclassified	unclassified	unclassified	unclassified	-0.200	-0.318	0.755	-0.237	0	0	1	0	0.75	0.016
**Arctic-N**	OTUp_730	Bacteria	WD272	uncultured_cyanobacterium	unclassified	unclassified	unclassified	unclassified	-0.295	-0.189	-0.357	0.841	0	0	0	1	0.84	0.000
**Arctic-N**	OTUp_749	Bacteria	Verrucomicrobia	OPB35_soil_group	uncultured_bacterium	unclassified	unclassified	unclassified	-0.377	-0.181	-0.202	0.759	0	0	0	1	0.76	0.002
**Arctic-N**	OTUp_773	Bacteria	Proteobacteria	Gammaproteobacteria	Xanthomonadales	Xanthomonadaceae	uncultured	unclassified	-0.449	-0.244	-0.219	0.912	0	0	0	1	0.91	0.002
**Arctic-N**	OTUp_815	Bacteria	WD272	uncultured_forest_soil_bacterium	unclassified	unclassified	unclassified	unclassified	-0.499	-0.213	-0.125	0.837	0	0	0	1	0.84	0.005
**Arctic-N**	OTUp_836	Bacteria	WD272	uncultured_Firmicutes_bacterium	unclassified	unclassified	unclassified	unclassified	-0.165	-0.082	-0.433	0.681	0	0	0	1	0.68	0.026
**Arctic-N**	OTUp_903	Bacteria	Acidobacteria	Acidobacteria	Subgroup_13	uncultured_Acidobacteria_bacterium	unclassified	unclassified	-0.147	0.377	-0.611	0.381	0	1	0	1	0.66	0.042
**Arctic-N**	OTUp_904	Bacteria	Planctomycetes	Planctomycetacia	Planctomycetales	Planctomycetaceae	uncultured	uncultured_planctomycete	-0.335	0.510	0.241	-0.416	0	1	1	0	0.65	0.039
**Arctic-N**	OTUp_940	Bacteria	Planctomycetes	Planctomycetacia	Planctomycetales	Planctomycetaceae	uncultured	unclassified	-0.397	0.086	-0.324	0.636	0	0	0	1	0.64	0.044
**Arctic-N**	OTUp_969	Bacteria	Planctomycetes	Planctomycetacia	Planctomycetales	Planctomycetaceae	Singulisphaera	uncultured_bacterium	-0.536	0.453	0.387	-0.305	0	1	1	0	0.73	0.014
**Arctic-N**	OTUp_1015	Bacteria	Proteobacteria	Betaproteobacteria	unclassified	unclassified	unclassified	unclassified	-0.278	-0.174	-0.203	0.655	0	0	0	1	0.66	0.043
**Arctic-N**	OTUp_1055	Bacteria	Planctomycetes	Planctomycetacia	Planctomycetales	Planctomycetaceae	uncultured	uncultured_bacterium	-0.661	0.356	0.148	0.157	0	1	1	1	0.66	0.045
**Arctic-N**	OTUp_1069	Bacteria	Verrucomicrobia	OPB35_soil_group	unclassified	unclassified	unclassified	unclassified	-0.231	-0.362	0.733	-0.140	0	0	1	0	0.73	0.010
**Arctic-N**	OTUp_1082	Bacteria	Proteobacteria	Alphaproteobacteria	Rickettsiales	Mitochondria	unclassified	unclassified	-0.180	-0.244	0.668	-0.244	0	0	1	0	0.67	0.027

**Arctic-N**	OTUp_1101	Bacteria	Acidobacteria	Acidobacteria	Subgroup_13	uncultured_Acidobacteria_bacterium	unclassified	unclassified	-0.428	-0.416	0.179	0.665	0	0	1	1	0.73	0.011
**Arctic-N**	OTUp_1171	Bacteria	Planctomycetes	Planctomycetacia	Planctomycetales	Planctomycetaceae	uncultured	unclassified	-0.465	0.303	0.498	-0.336	0	1	1	0	0.69	0.024
**Arctic-N**	OTUp_1218	Bacteria	Verrucomicrobia	Spartobacteria	Chthoniobacterales	Chthoniobacteraceae	Chthoniobacter	bacterium_Ellin506	-0.173	-0.488	-0.127	0.788	0	0	0	1	0.79	0.006
**Arctic-N**	OTUp_1296	Bacteria	Chlorobi	Chlorobia	Chlorobiales	OPB56	uncultured_bacterium	unclassified	-0.477	0.404	-0.291	0.365	0	1	0	1	0.67	0.029
**Arctic-N**	OTUp_1326	Bacteria	Planctomycetes	Phycisphaerae	WD2101_soil_group	uncultured_bacterium	unclassified	unclassified	-0.457	0.405	-0.299	0.351	0	1	0	1	0.65	0.050
**Arctic-N**	OTUp_1344	Bacteria	Parcubacteria	uncultured_bacterium	unclassified	unclassified	unclassified	unclassified	-0.303	-0.303	-0.080	0.686	0	0	0	1	0.69	0.027
**Arctic-N**	OTUp_1400	Bacteria	Proteobacteria	Betaproteobacteria	TRA3-20	uncultured_beta_proteobacterium	unclassified	unclassified	-0.226	-0.255	-0.253	0.733	0	0	0	1	0.73	0.024
**Arctic-N**	OTUp_1402	Bacteria	Planctomycetes	Planctomycetacia	Planctomycetales	Planctomycetaceae	uncultured	uncultured_bacterium	-0.518	0.632	-0.239	0.124	0	1	0	1	0.66	0.043
**Arctic-N**	OTUp_1434	Bacteria	Acidobacteria	Acidobacteria	Subgroup_2	uncultured_forest_soil_bacterium	unclassified	unclassified	-0.317	-0.332	-0.103	0.751	0	0	0	1	0.75	0.012
**Arctic-N**	OTUp_1435	Bacteria	Proteobacteria	Betaproteobacteria	Nitrosomonadales	Nitrosomonadaceae	uncultured	unclassified	-0.218	-0.173	-0.305	0.695	0	0	0	1	0.70	0.017
**Arctic-N**	OTUp_1581	Bacteria	Chloroflexi	Ktedonobacteria	Ktedonobacterales	1921-3	uncultured_bacterium	unclassified	-0.533	0.506	-0.304	0.331	0	1	0	1	0.73	0.019
**Arctic-N**	OTUp_1599	Bacteria	Saccharibacteria	bacterium_LWQ8	unclassified	unclassified	unclassified	unclassified	-0.397	-0.289	0.019	0.667	0	0	0	1	0.67	0.027
**Arctic-N**	OTUp_1602	Bacteria	Verrucomicrobia	Spartobacteria	Chthoniobacterales	DA101_soil_group	uncultured_bacterium	unclassified	-0.402	0.156	-0.495	0.741	0	1	0	1	0.78	0.005
**Arctic-N**	OTUp_1665	Bacteria	Verrucomicrobia	OPB35_soil_group	uncultured_bacterium	unclassified	unclassified	unclassified	-0.509	-0.139	-0.030	0.678	0	0	0	1	0.68	0.026
**Arctic-N**	OTUp_1699	Bacteria	Verrucomicrobia	Spartobacteria	Chthoniobacterales	Chthoniobacteraceae	Chthoniobacter	uncultured_bacterium	-0.392	-0.205	-0.130	0.727	0	0	0	1	0.73	0.011
**Arctic-N**	OTUp_1709	Bacteria	Planctomycetes	Phycisphaerae	WD2101_soil_group	uncultured_bacterium	unclassified	unclassified	-0.287	-0.018	-0.339	0.644	0	0	0	1	0.64	0.035
**Arctic-N**	OTUp_1797	Bacteria	Acidobacteria	Acidobacteria	Subgroup_4	Unknown_Family	Blastocatella	unclassified	-0.242	-0.322	-0.190	0.754	0	0	0	1	0.75	0.008
**Arctic-N**	OTUp_1846	Bacteria	Verrucomicrobia	Spartobacteria	Chthoniobacterales	DA101_soil_group	unclassified	unclassified	-0.442	-0.015	-0.278	0.735	0	0	0	1	0.73	0.013
**Arctic-N**	OTUp_1939	Bacteria	unclassified	unclassified	unclassified	unclassified	unclassified	unclassified	-0.062	-0.325	0.706	-0.319	0	0	1	0	0.71	0.039
**Arctic-N**	OTUp_1946	Bacteria	Actinobacteria	Acidimicrobiia	Acidimicrobiales	uncultured	Acidimicrobidae_bacterium_Ellin7143	unclassified	-0.378	0.284	0.507	-0.414	0	1	1	0	0.69	0.029
**Arctic-N**	OTUp_1951	Bacteria	Verrucomicrobia	Verrucomicrobiae	Verrucomicrobiales	Verrucomicrobiaceae	uncultured	unclassified	-0.313	0.401	-0.470	0.383	0	1	0	1	0.68	0.019
**Arctic-N**	OTUp_2031	Bacteria	Planctomycetes	Phycisphaerae	WD2101_soil_group	uncultured_bacterium	unclassified	unclassified	-0.277	-0.277	-0.277	0.831	0	0	0	1	0.83	0.029
**Arctic-N**	OTUp_2035	Bacteria	Verrucomicrobia	Spartobacteria	Chthoniobacterales	Chthoniobacteraceae	Chthoniobacter	unclassified	-0.295	-0.219	0.757	-0.242	0	0	1	0	0.76	0.008
**Arctic-N**	OTUp_2103	Bacteria	Proteobacteria	Alphaproteobacteria	Rhodospirillales	Acetobacteraceae	unclassified	unclassified	-0.126	-0.054	0.772	-0.591	0	0	1	0	0.77	0.005
**Arctic-N**	OTUp_2210	Bacteria	Chloroflexi	Thermomicrobia	JG30-KF-CM45	uncultured_bacterium	unclassified	unclassified	-0.023	-0.447	0.745	-0.274	0	0	1	0	0.74	0.023
**Arctic-N**	OTUp_2252	Bacteria	Bacteroidetes	Sphingobacteriia	Sphingobacteriales	Chitinophagaceae	uncultured	uncultured_bacterium	-0.414	0.247	-0.349	0.516	0	1	0	1	0.66	0.037
**Arctic-N**	OTUp_2272	Bacteria	Gemmatimonadetes	Gemmatimonadetes	Gemmatimonadales	Gemmatimonadaceae	Gemmatimonas	uncultured_bacterium	-0.341	0.181	-0.481	0.641	0	1	0	1	0.71	0.022
**Arctic-N**	OTUp_2296	Bacteria	Planctomycetes	Planctomycetacia	Planctomycetales	Planctomycetaceae	uncultured	uncultured_bacterium	-0.255	-0.131	0.737	-0.351	0	0	1	0	0.74	0.003
**Arctic-N**	OTUp_2299	Bacteria	Proteobacteria	Betaproteobacteria	SC-I-84	uncultured_bacterium	unclassified	unclassified	-0.311	-0.378	0.025	0.664	0	0	0	1	0.66	0.043
**Arctic-N**	OTUp_2311	Bacteria	Acidobacteria	Acidobacteria	Subgroup_2	uncultured_Acidobacteria_bacterium	unclassified	unclassified	-0.256	-0.321	-0.149	0.725	0	0	0	1	0.73	0.015
**Arctic-N**	OTUp_2373	Bacteria	Proteobacteria	Gammaproteobacteria	Xanthomonadales	Xanthomonadaceae	unclassified	unclassified	-0.168	-0.559	-0.182	0.910	0	0	0	1	0.91	0.002
**Arctic-N**	OTUp_2442	Bacteria	Acidobacteria	Acidobacteria	Subgroup_4	Unknown_Family	Blastocatella	uncultured_Acidobacteria_bacterium	-0.176	-0.239	-0.262	0.677	0	0	0	1	0.68	0.027
**Arctic-N**	OTUp_2447	Bacteria	Actinobacteria	Actinobacteria	Frankiales	Acidothermaceae	Acidothermus	uncultured_bacterium	-0.103	-0.234	0.668	-0.332	0	0	1	0	0.67	0.028
**Arctic-N**	OTUp_2598	Bacteria	Verrucomicrobia	Spartobacteria	Chthoniobacterales	DA101_soil_group	uncultured_Spartobacteria_bacterium	unclassified	-0.558	0.367	-0.381	0.572	0	1	0	1	0.81	0.001
**Arctic-N**	OTUp_2633	Bacteria	Verrucomicrobia	Spartobacteria	Chthoniobacterales	DA101_soil_group	uncultured_bacterium	unclassified	-0.548	0.269	-0.207	0.487	0	1	0	1	0.65	0.027
**Arctic-N**	OTUp_2648	Bacteria	Bacteroidetes	Sphingobacteriia	Sphingobacteriales	Chitinophagaceae	Taibaiella	unclassified	-0.270	-0.320	-0.213	0.802	0	0	0	1	0.80	0.003
**Arctic-N**	OTUp_2764	Bacteria	Planctomycetes	Planctomycetacia	Planctomycetales	Planctomycetaceae	Gemmata	uncultured_bacterium	-0.746	0.222	0.187	0.336	0	1	1	1	0.75	0.011
**Arctic-N**	OTUp_2859	Bacteria	Planctomycetes	Planctomycetacia	Planctomycetales	Planctomycetaceae	uncultured	uncultured_bacterium	-0.373	-0.261	0.780	-0.147	0	0	1	0	0.78	0.008
**Arctic-N**	OTUp_2899	Bacteria	Parcubacteria	uncultured_bacterium	unclassified	unclassified	unclassified	unclassified	-0.253	-0.253	0.760	-0.253	0	0	1	0	0.76	0.029
**Arctic-N**	OTUp_3230	Bacteria	unclassified	unclassified	unclassified	unclassified	unclassified	unclassified	-0.455	-0.296	0.761	-0.009	0	0	1	0	0.76	0.009
**Arctic-N**	OTUp_3329	Bacteria	Cyanobacteria	Chloroplast	unclassified	unclassified	unclassified	unclassified	-0.227	-0.227	0.681	-0.227	0	0	1	0	0.68	0.002
**Arctic-N**	OTUp_3351	Bacteria	Verrucomicrobia	OPB35_soil_group	uncultured_bacterium	unclassified	unclassified	unclassified	-0.167	-0.405	-0.227	0.799	0	0	0	1	0.80	0.007
**Arctic-N**	OTUp_3552	Bacteria	Verrucomicrobia	Opitutae	Opitutales	Opitutaceae	Opitutus	uncultured_bacterium	-0.292	-0.290	-0.078	0.660	0	0	0	1	0.66	0.040
**Arctic-N**	OTUp_3640	Bacteria	Chloroflexi	Ktedonobacteria	Ktedonobacterales	Ktedonobacteraceae	uncultured	uncultured_Clostridiales_bacterium	-0.007	-0.378	-0.290	0.675	0	0	0	1	0.67	0.032
**Arctic-N**	OTUp_3724	Bacteria	Verrucomicrobia	Spartobacteria	Chthoniobacterales	DA101_soil_group	uncultured_Verrucomicrobia_bacterium	unclassified	-0.106	-0.262	-0.360	0.728	0	0	0	1	0.73	0.011
**Arctic-N**	OTUp_3739	Bacteria	Planctomycetes	Planctomycetacia	Planctomycetales	Planctomycetaceae	uncultured	uncultured_planctomycete	-0.440	0.563	0.304	-0.427	0	1	1	0	0.75	0.007
**Arctic-N**	OTUp_3748	Bacteria	Planctomycetes	Phycisphaerae	WD2101_soil_group	unclassified	unclassified	unclassified	-0.216	-0.216	0.647	-0.216	0	0	1	0	0.65	0.029
**Arctic-N**	OTUp_3766	Bacteria	Proteobacteria	Betaproteobacteria	Burkholderiales	Oxalobacteraceae	unclassified	unclassified	-0.266	-0.266	0.798	-0.266	0	0	1	0	0.80	0.029
**Arctic-N**	OTUp_3767	Bacteria	Planctomycetes	Planctomycetacia	Planctomycetales	Planctomycetaceae	uncultured	unclassified	-0.380	0.625	-0.380	0.135	0	1	0	1	0.66	0.019
**Arctic-N**	OTUp_3771	Bacteria	Planctomycetes	Planctomycetacia	Planctomycetales	Planctomycetaceae	uncultured	unclassified	-0.285	-0.272	-0.174	0.731	0	0	0	1	0.73	0.022
**Arctic-N**	OTUp_3792	Bacteria	Armatimonadetes	unclassified	unclassified	unclassified	unclassified	unclassified	-0.306	-0.306	-0.306	0.917	0	0	0	1	0.92	0.002
**Arctic-N**	OTUp_3869	Bacteria	Proteobacteria	Alphaproteobacteria	Caulobacterales	Hyphomonadaceae	Hirschia	uncultured_bacterium	-0.333	-0.333	-0.033	0.698	0	0	0	1	0.70	0.027
**Arctic-N**	OTUp_3878	Bacteria	Bacteroidetes	Sphingobacteriia	Sphingobacteriales	Chitinophagaceae	uncultured	uncultured_bacterium	-0.065	-0.312	0.648	-0.270	0	0	1	0	0.65	0.018
**Arctic-N**	OTUp_3961	Bacteria	Verrucomicrobia	Spartobacteria	Chthoniobacterales	DA101_soil_group	unclassified	unclassified	-0.380	-0.036	-0.230	0.646	0	0	0	1	0.65	0.046
**Arctic-N**	OTUp_4075	Bacteria	Actinobacteria	Thermoleophilia	Solirubrobacterales	unclassified	unclassified	unclassified	-0.211	-0.313	0.819	-0.296	0	0	1	0	0.82	0.002
**Arctic-N**	OTUp_4293	Bacteria	Verrucomicrobia	Spartobacteria	Chthoniobacterales	DA101_soil_group	uncultured_bacterium	unclassified	-0.423	-0.089	-0.327	0.839	0	0	0	1	0.84	0.002
**Arctic-N**	OTUp_4449	Bacteria	Chloroflexi	JG37-AG-4	unclassified	unclassified	unclassified	unclassified	-0.034	-0.269	-0.383	0.687	0	0	0	1	0.69	0.026
**Arctic-N**	OTUp_4482	Bacteria	WD272	uncultured_bacterium	unclassified	unclassified	unclassified	unclassified	-0.116	-0.475	-0.115	0.706	0	0	0	1	0.71	0.013
**Arctic-N**	OTUp_4588	Bacteria	Parcubacteria	uncultured_bacterium	unclassified	unclassified	unclassified	unclassified	-0.264	-0.264	-0.264	0.791	0	0	0	1	0.79	0.027
**Arctic-N**	OTUp_4789	Bacteria	Chloroflexi	Anaerolineae	Anaerolineales	Anaerolineaceae	uncultured	uncultured_Gemmatimonadetes_bacterium	-0.138	-0.149	0.702	-0.415	0	0	1	0	0.70	0.038
**Arctic-N**	OTUp_4887	Bacteria	Verrucomicrobia	Spartobacteria	Chthoniobacterales	DA101_soil_group	uncultured_soil_bacterium	unclassified	-0.399	-0.056	-0.310	0.765	0	0	0	1	0.77	0.006
**Arctic-N**	OTUp_5019	Bacteria	Verrucomicrobia	Spartobacteria	Chthoniobacterales	DA101_soil_group	uncultured_Verrucomicrobia_bacterium	unclassified	-0.363	-0.009	-0.283	0.656	0	0	0	1	0.66	0.037
**Arctic-N**	OTUp_5118	Bacteria	Verrucomicrobia	Spartobacteria	Chthoniobacterales	DA101_soil_group	uncultured_soil_bacterium	unclassified	-0.436	0.524	-0.474	0.386	0	1	0	1	0.79	0.004
**Arctic-N**	OTUp_5205	Bacteria	Acidobacteria	Acidobacteria	Subgroup_3	Unknown_Family	Candidatus_Solibacter	uncultured_Acidobacteria_bacterium	-0.422	-0.361	0.331	0.451	0	0	1	1	0.68	0.033
**Arctic-N**	OTUp_5244	Bacteria	Chloroflexi	Ktedonobacteria	Thermogemmatisporales	1921-2	uncultured_bacterium	unclassified	-0.685	0.490	0.044	0.152	0	1	1	1	0.69	0.020
**Arctic-N**	OTUp_5282	Bacteria	Acidobacteria	Acidobacteria	Acidobacteriales	Acidobacteriaceae	uncultured	uncultured_bacterium	0.017	-0.570	-0.119	0.672	0	0	0	1	0.67	0.027
**Arctic-N**	OTUp_5429	Bacteria	Planctomycetes	Planctomycetacia	Planctomycetales	Planctomycetaceae	Gemmata	uncultured_bacterium	-0.270	-0.425	-0.061	0.755	0	0	0	1	0.76	0.009
**Arctic-N**	OTUp_5464	Bacteria	Chloroflexi	Anaerolineae	Anaerolineales	Anaerolineaceae	uncultured	uncultured_Gemmatimonadetes_bacterium	-0.271	-0.515	0.204	0.582	0	0	1	1	0.68	0.024
**Arctic-N**	OTUp_5621	Bacteria	Proteobacteria	Betaproteobacteria	Burkholderiales	Oxalobacteraceae	Massilia	uncultured_bacterium	-0.325	-0.286	0.798	-0.186	0	0	1	0	0.80	0.010
**Arctic-N**	OTUp_5638	Bacteria	Chloroflexi	Ktedonobacteria	Ktedonobacterales	Ktedonobacteraceae	Ktedonobacter	uncultured_Chloroflexi_bacterium	-0.143	-0.295	-0.307	0.745	0	0	0	1	0.75	0.013
**Arctic-N**	OTUp_5755	Bacteria	Proteobacteria	TA18	uncultured_Firmicutes_bacterium	unclassified	unclassified	unclassified	-0.645	0.578	-0.031	0.098	0	1	1	1	0.64	0.043
**Arctic-N**	OTUp_6001	Bacteria	Bacteroidetes	Sphingobacteriia	Sphingobacteriales	Sphingobacteriaceae	Mucilaginibacter	unclassified	-0.198	-0.267	-0.382	0.847	0	0	0	1	0.85	0.002
**Arctic-N**	OTUp_6035	Bacteria	Verrucomicrobia	Spartobacteria	Chthoniobacterales	DA101_soil_group	uncultured_Prosthecobacter_sp.	unclassified	-0.317	-0.122	-0.389	0.829	0	0	0	1	0.83	0.002
**Arctic-N**	OTUp_6341	Bacteria	Verrucomicrobia	Spartobacteria	Chthoniobacterales	DA101_soil_group	unclassified	unclassified	-0.356	-0.093	-0.288	0.737	0	0	0	1	0.74	0.009
**Arctic-N**	OTUp_6466	Bacteria	Armatimonadetes	uncultured_Carnobacterium_sp.	unclassified	unclassified	unclassified	unclassified	-0.196	-0.205	-0.233	0.635	0	0	0	1	0.64	0.047
**Arctic-N**	OTUp_6670	Bacteria	Planctomycetes	Planctomycetacia	Planctomycetales	Planctomycetaceae	uncultured	uncultured_bacterium	-0.248	-0.248	0.743	-0.248	0	0	1	0	0.74	0.029
**Arctic-N**	OTUp_6742	Bacteria	Planctomycetes	Planctomycetacia	Planctomycetales	Planctomycetaceae	uncultured	unclassified	-0.432	0.502	-0.321	0.251	0	1	0	1	0.65	0.039
**Arctic-N**	OTUp_6798	Bacteria	Bacteroidetes	Cytophagia	Cytophagales	Cytophagaceae	Hymenobacter	unclassified	-0.258	-0.258	0.774	-0.258	0	0	1	0	0.77	0.027
**Arctic-N**	OTUp_7294	Bacteria	Verrucomicrobia	Spartobacteria	Chthoniobacterales	DA101_soil_group	uncultured_Verrucomicrobia_bacterium	unclassified	-0.618	0.102	-0.158	0.674	0	0	0	1	0.67	0.036
**Arctic-N**	OTUp_7501	Bacteria	Proteobacteria	Gammaproteobacteria	NKB5	uncultured_bacterium	unclassified	unclassified	-0.234	-0.234	-0.234	0.702	0	0	0	1	0.70	0.027
**Arctic-N**	OTUp_7607	Bacteria	Elusimicrobia	Elusimicrobia	FCPU453	uncultured_bacterium	unclassified	unclassified	-0.401	-0.401	0.763	0.040	0	0	1	0	0.76	0.009
**Arctic-N**	OTUp_7707	Bacteria	Planctomycetes	Planctomycetacia	Planctomycetales	Planctomycetaceae	uncultured	unclassified	-0.678	0.490	-0.083	0.270	0	1	1	1	0.68	0.031
**Arctic-N**	OTUp_8161	Bacteria	Proteobacteria	Alphaproteobacteria	Rhodospirillales	Acetobacteraceae	unclassified	unclassified	-0.232	-0.223	0.791	-0.337	0	0	1	0	0.79	0.004
**Arctic-N**	OTUp_8485	Bacteria	Chloroflexi	Ktedonobacteria	unclassified	unclassified	unclassified	unclassified	-0.124	-0.446	0.720	-0.150	0	0	1	0	0.72	0.036
**Arctic-N**	OTUp_8584	Bacteria	Chloroflexi	Ktedonobacteria	Ktedonobacterales	HSB_OF53-F07	unclassified	unclassified	-0.467	-0.093	-0.176	0.736	0	0	0	1	0.74	0.012
**Arctic-N**	OTUp_8874	Bacteria	Proteobacteria	TA18	uncultured_Firmicutes_bacterium	unclassified	unclassified	unclassified	-0.278	-0.321	0.747	-0.148	0	0	1	0	0.75	0.017
**Arctic-N**	OTUp_8930	Bacteria	WD272	uncultured_bacterium	unclassified	unclassified	unclassified	unclassified	-0.551	-0.283	0.512	0.322	0	0	1	1	0.72	0.012
**Arctic-N**	OTUp_9069	Bacteria	Proteobacteria	Gammaproteobacteria	Xanthomonadales	Xanthomonadaceae	unclassified	unclassified	-0.608	-0.314	0.175	0.746	0	0	1	1	0.80	0.002
**Arctic-N**	OTUp_9226	Bacteria	Actinobacteria	Acidimicrobiia	Acidimicrobiales	uncultured	uncultured_bacterium	unclassified	-0.317	-0.317	0.711	-0.077	0	0	1	0	0.71	0.026
**Arctic-N**	OTUp_9991	Bacteria	Verrucomicrobia	Spartobacteria	Chthoniobacterales	Chthoniobacteraceae	Chthoniobacter	unclassified	-0.416	-0.416	0.436	0.397	0	0	1	1	0.72	0.034
**Arctic-N**	OTUp_10215	Bacteria	Actinobacteria	Actinobacteria	Frankiales	Acidothermaceae	Acidothermus	uncultured_actinobacterium	-0.137	-0.209	0.603	-0.257	0	0	1	0	0.60	0.025
**Arctic-N**	OTUp_10216	Bacteria	TM6	uncultured_bacterium	unclassified	unclassified	unclassified	unclassified	-0.266	-0.266	0.799	-0.266	0	0	1	0	0.80	0.029
**Arctic-N**	OTUp_10293	Bacteria	Planctomycetes	Planctomycetacia	Planctomycetales	Planctomycetaceae	Isosphaera	uncultured_planctomycete	-0.317	-0.317	0.711	-0.077	0	0	1	0	0.71	0.026
**Arctic-N**	OTUp_10359	Bacteria	Chloroflexi	Ktedonobacteria	Thermogemmatisporales	1921-2	uncultured_bacterium	unclassified	-0.562	0.525	-0.261	0.298	0	1	0	1	0.71	0.018
**Arctic-N**	OTUp_10757	Bacteria	Acidobacteria	Acidobacteria	Subgroup_3	Unknown_Family	Bryobacter	uncultured_bacterium	-0.513	-0.060	0.653	-0.081	0	0	1	0	0.65	0.030
**Arctic-N**	OTUp_10824	Bacteria	Acidobacteria	Acidobacteria	Subgroup_2	unclassified	unclassified	unclassified	-0.522	-0.252	0.183	0.592	0	0	1	1	0.67	0.037

**Arctic-N**	OTUp_11251	Bacteria	Planctomycetes	Planctomycetacia	Planctomycetales	Planctomycetaceae	uncultured	uncultured_bacterium	-0.456	0.039	0.658	-0.242	0	0	1	0	0.66	0.034
**Arctic-S**	OTUp_5	Bacteria	Gemmatimonadetes	Gemmatimonadetes	Gemmatimonadales	Gemmatimonadaceae	uncultured	uncultured_soil_bacterium	-0.044	-0.279	-0.334	0.657	0	0	0	1	0.66	0.031
**Arctic-S**	OTUp_9	Bacteria	Chloroflexi	JG37-AG-4	uncultured_bacterium	unclassified	unclassified	unclassified	-0.273	-0.500	0.438	0.336	0	0	1	1	0.67	0.034
**Arctic-S**	OTUp_11	Bacteria	Verrucomicrobia	Spartobacteria	Chthoniobacterales	DA101_soil_group	uncultured_soil_bacterium	unclassified	-0.479	-0.350	0.107	0.721	0	0	0	1	0.72	0.014
**Arctic-S**	OTUp_12	Bacteria	Verrucomicrobia	Spartobacteria	Chthoniobacterales	DA101_soil_group	uncultured_Verrucomicrobia_bacterium	unclassified	-0.213	-0.162	-0.257	0.632	0	0	0	1	0.63	0.043
**Arctic-S**	OTUp_14	Bacteria	Acidobacteria	Acidobacteria	Subgroup_2	uncultured_Acidobacteria_bacterium	unclassified	unclassified	-0.329	-0.298	-0.001	0.629	0	0	0	1	0.63	0.037
**Arctic-S**	OTUp_15	Bacteria	Proteobacteria	Betaproteobacteria	SC-I-84	uncultured_beta_proteobacterium	unclassified	unclassified	-0.508	-0.396	0.119	0.785	0	0	0	1	0.78	0.006
**Arctic-S**	OTUp_17	Bacteria	Acidobacteria	Acidobacteria	Acidobacteriales	Acidobacteriaceae	uncultured	uncultured_Acidobacteriaceae_bacterium	-0.482	-0.415	0.331	0.566	0	0	1	1	0.78	0.006
**Arctic-S**	OTUp_20	Bacteria	Verrucomicrobia	Spartobacteria	Chthoniobacterales	DA101_soil_group	uncultured_Verrucomicrobia_bacterium	unclassified	-0.379	-0.519	0.164	0.733	0	0	1	1	0.78	0.008
**Arctic-S**	OTUp_22	Bacteria	Acidobacteria	Acidobacteria	Subgroup_4	RB41	uncultured_Acidobacteria_bacterium	unclassified	-0.397	-0.299	-0.009	0.704	0	0	0	1	0.70	0.015
**Arctic-S**	OTUp_24	Bacteria	Acidobacteria	Acidobacteria	Subgroup_2	uncultured_Acidobacteria_bacterium	unclassified	unclassified	-0.041	-0.487	-0.112	0.639	0	0	0	1	0.64	0.024
**Arctic-S**	OTUp_26	Bacteria	Proteobacteria	Gammaproteobacteria	Xanthomonadales	Xanthomonadales_Incertae_Sedis	Acidibacter	uncultured_gamma_proteobacterium	-0.406	-0.432	0.529	0.308	0	0	1	1	0.73	0.014
**Arctic-S**	OTUp_28	Bacteria	Proteobacteria	Gammaproteobacteria	Xanthomonadales	uncultured	uncultured_eubacterium_WD260	unclassified	-0.278	-0.614	0.577	0.314	0	0	1	1	0.77	0.006
**Arctic-S**	OTUp_30	Bacteria	Verrucomicrobia	Spartobacteria	Chthoniobacterales	Xiphinematobacteraceae	Candidatus_Xiphinematobacter	uncultured_bacterium	-0.369	-0.454	0.454	0.368	0	0	1	1	0.71	0.015
**Arctic-S**	OTUp_31	Bacteria	Actinobacteria	Actinobacteria	Micrococcales	Micrococcaceae	Arthrobacter	unclassified	-0.032	-0.507	0.846	-0.307	0	0	1	0	0.85	0.002
**Arctic-S**	OTUp_32	Bacteria	Acidobacteria	Acidobacteria	Subgroup_3	Unknown_Family	Bryobacter	uncultured_bacterium	-0.316	-0.256	-0.185	0.757	0	0	0	1	0.76	0.004
**Arctic-S**	OTUp_34	Bacteria	Proteobacteria	Gammaproteobacteria	Xanthomonadales	Xanthomonadaceae	Rhodanobacter	uncultured_Xanthomonadaceae_bacterium	-0.382	-0.544	0.300	0.626	0	0	1	1	0.80	0.001
**Arctic-S**	OTUp_39	Bacteria	Acidobacteria	Acidobacteria	Acidobacteriales	Acidobacteriaceae	Granulicella	uncultured_bacterium	-0.417	-0.373	0.379	0.411	0	0	1	1	0.68	0.033
**Arctic-S**	OTUp_50	Bacteria	WD272	unclassified	unclassified	unclassified	unclassified	unclassified	-0.633	-0.225	0.147	0.712	0	0	1	1	0.74	0.009
**Arctic-S**	OTUp_51	Bacteria	Bacteroidetes	Sphingobacteriia	Sphingobacteriales	Chitinophagaceae	uncultured	unclassified	-0.247	-0.632	0.097	0.783	0	0	0	1	0.78	0.002
**Arctic-S**	OTUp_53	Bacteria	Acidobacteria	Acidobacteria	Subgroup_3	Unknown_Family	Candidatus_Solibacter	uncultured_Acidobacteriales_bacterium	-0.567	-0.380	0.455	0.493	0	0	1	1	0.82	0.002
**Arctic-S**	OTUp_55	Bacteria	Acidobacteria	Holophagae	Subgroup_7	uncultured_Acidobacteria_bacterium	unclassified	unclassified	-0.175	-0.304	-0.187	0.666	0	0	0	1	0.67	0.008
**Arctic-S**	OTUp_56	Bacteria	Chloroflexi	S085	uncultured_bacterium	unclassified	unclassified	unclassified	-0.480	-0.449	0.354	0.575	0	0	1	1	0.80	0.003
**Arctic-S**	OTUp_58	Bacteria	Acidobacteria	Acidobacteria	Subgroup_2	uncultured_Acidobacteria_bacterium	unclassified	unclassified	-0.502	-0.292	0.269	0.526	0	0	1	1	0.69	0.021
**Arctic-S**	OTUp_59	Bacteria	Actinobacteria	Thermoleophilia	Gaiellales	Gaiellaceae	Gaiella	unclassified	-0.336	-0.519	0.757	0.098	0	0	1	0	0.76	0.006
**Arctic-S**	OTUp_61	Bacteria	Verrucomicrobia	Spartobacteria	Chthoniobacterales	DA101_soil_group	uncultured_Spartobacteria_bacterium	unclassified	-0.695	0.128	-0.118	0.684	0	1	0	1	0.70	0.017
**Arctic-S**	OTUp_77	Bacteria	Verrucomicrobia	Spartobacteria	Chthoniobacterales	DA101_soil_group	uncultured_Verrucomicrobia_bacterium	unclassified	-0.465	-0.447	0.283	0.629	0	0	1	1	0.79	0.004
**Arctic-S**	OTUp_94	Bacteria	Actinobacteria	Actinobacteria	Corynebacteriales	Mycobacteriaceae	Mycobacterium	unclassified	-0.322	-0.258	0.675	-0.095	0	0	1	0	0.68	0.024
**Arctic-S**	OTUp_97	Bacteria	Verrucomicrobia	Spartobacteria	Chthoniobacterales	DA101_soil_group	unclassified	unclassified	-0.409	-0.369	0.123	0.655	0	0	1	1	0.67	0.033
**Arctic-S**	OTUp_104	Bacteria	Verrucomicrobia	Spartobacteria	Chthoniobacterales	DA101_soil_group	uncultured_bacterium	unclassified	-0.195	-0.451	-0.017	0.663	0	0	0	1	0.66	0.038
**Arctic-S**	OTUp_108	Bacteria	Planctomycetes	Planctomycetacia	Planctomycetales	Planctomycetaceae	Gemmata	uncultured_bacterium	-0.480	-0.321	0.166	0.636	0	0	1	1	0.69	0.018
**Arctic-S**	OTUp_114	Bacteria	Proteobacteria	Betaproteobacteria	Burkholderiales	unclassified	unclassified	unclassified	-0.218	-0.540	0.291	0.467	0	0	1	1	0.66	0.044
**Arctic-S**	OTUp_117	Bacteria	Acidobacteria	Acidobacteria	Acidobacteriales	Acidobacteriaceae	unclassified	unclassified	-0.410	-0.420	0.156	0.673	0	0	1	1	0.72	0.011
**Arctic-S**	OTUp_127	Bacteria	Acidobacteria	Holophagae	Subgroup_7	uncultured_proteobacterium	unclassified	unclassified	-0.594	-0.346	0.420	0.520	0	0	1	1	0.81	0.002
**Arctic-S**	OTUp_132	Bacteria	Proteobacteria	Gammaproteobacteria	Xanthomonadales	Xanthomonadaceae	Silanimonas	uncultured_Xanthomonadaceae_bacterium	-0.185	-0.675	0.532	0.328	0	0	1	1	0.74	0.007
**Arctic-S**	OTUp_137	Bacteria	Planctomycetes	Planctomycetacia	Planctomycetales	Planctomycetaceae	uncultured	unclassified	-0.192	0.407	0.348	-0.563	0	1	1	0	0.65	0.041
**Arctic-S**	OTUp_138	Bacteria	Proteobacteria	Gammaproteobacteria	Xanthomonadales	Xanthomonadaceae	unclassified	unclassified	-0.653	-0.300	0.196	0.756	0	0	1	1	0.82	0.001
**Arctic-S**	OTUp_148	Bacteria	Planctomycetes	Phycisphaerae	WD2101_soil_group	uncultured_bacterium	unclassified	unclassified	-0.503	0.444	0.384	-0.325	0	1	1	0	0.72	0.020
**Arctic-S**	OTUp_151	Bacteria	Proteobacteria	Gammaproteobacteria	Xanthomonadales	uncultured	uncultured_proteobacterium	unclassified	-0.272	-0.681	0.292	0.660	0	0	1	1	0.83	0.002
**Arctic-S**	OTUp_166	Bacteria	Chloroflexi	Thermomicrobia	JG30-KF-CM45	uncultured_Chloroflexi_bacterium	unclassified	unclassified	-0.466	0.207	-0.296	0.555	0	1	0	1	0.66	0.021
**Arctic-S**	OTUp_168	Bacteria	Acidobacteria	Holophagae	Subgroup_7	unclassified	unclassified	unclassified	-0.332	-0.271	-0.212	0.815	0	0	0	1	0.82	0.002
**Arctic-S**	OTUp_169	Bacteria	Chloroflexi	Chloroflexia	Kallotenuales	AKIW781	uncultured_bacterium	unclassified	-0.443	-0.443	0.548	0.339	0	0	1	1	0.77	0.002
**Arctic-S**	OTUp_171	Bacteria	Planctomycetes	Planctomycetacia	Planctomycetales	Planctomycetaceae	Gemmata	uncultured_soil_bacterium	-0.353	-0.410	0.169	0.594	0	0	1	1	0.66	0.037
**Arctic-S**	OTUp_172	Bacteria	Proteobacteria	Alphaproteobacteria	Sphingomonadales	Sphingomonadaceae	Sphingomonas	Sphingomonas_faeni	-0.339	-0.466	0.692	0.112	0	0	1	1	0.70	0.017
**Arctic-S**	OTUp_177	Bacteria	Verrucomicrobia	Spartobacteria	Chthoniobacterales	DA101_soil_group	uncultured_soil_bacterium	unclassified	-0.410	-0.279	0.043	0.646	0	0	0	1	0.65	0.039
**Arctic-S**	OTUp_187	Bacteria	Gemmatimonadetes	Gemmatimonadetes	Gemmatimonadales	Gemmatimonadaceae	uncultured	uncultured_Gemmatimonadales_bacterium	-0.579	-0.353	0.580	0.353	0	0	1	1	0.81	0.002
**Arctic-S**	OTUp_194	Bacteria	Acidobacteria	Acidobacteria	Acidobacteriales	Acidobacteriaceae	uncultured	uncultured_bacterium	-0.457	-0.622	0.480	0.599	0	0	1	1	0.93	0.001
**Arctic-S**	OTUp_200	Bacteria	Chloroflexi	Ktedonobacteria	Ktedonobacterales	Ktedonobacteraceae	uncultured	uncultured_Chloroflexi_bacterium	-0.605	-0.257	0.649	0.214	0	0	1	1	0.75	0.009
**Arctic-S**	OTUp_203	Bacteria	Actinobacteria	Acidimicrobiia	Acidimicrobiales	uncultured	uncultured_Acidimicrobiaceae_bacterium	unclassified	-0.348	-0.150	0.739	-0.241	0	0	1	0	0.74	0.007
**Arctic-S**	OTUp_205	Bacteria	Acidobacteria	Acidobacteria	Subgroup_4	RB41	unclassified	unclassified	-0.296	-0.494	0.171	0.619	0	0	1	1	0.68	0.029
**Arctic-S**	OTUp_208	Bacteria	Acidobacteria	Acidobacteria	Subgroup_4	Unknown_Family	Blastocatella	uncultured_soil_bacterium	-0.365	-0.233	-0.265	0.863	0	0	0	1	0.86	0.002
**Arctic-S**	OTUp_210	Bacteria	Acidobacteria	Acidobacteria	Subgroup_6	unclassified	unclassified	unclassified	-0.637	-0.097	0.063	0.670	0	0	0	1	0.67	0.042
**Arctic-S**	OTUp_216	Bacteria	Proteobacteria	Alphaproteobacteria	Rhizobiales	Xanthobacteraceae	Variibacter	uncultured_Hyphomicrobiaceae_bacterium	-0.130	-0.607	0.051	0.687	0	0	0	1	0.69	0.031
**Arctic-S**	OTUp_222	Bacteria	Planctomycetes	Planctomycetacia	Planctomycetales	Planctomycetaceae	Gemmata	uncultured_planctomycete	-0.021	-0.501	-0.149	0.672	0	0	0	1	0.67	0.033
**Arctic-S**	OTUp_227	Bacteria	WD272	uncultured_bacterium	unclassified	unclassified	unclassified	unclassified	-0.387	-0.108	-0.178	0.673	0	0	0	1	0.67	0.020
**Arctic-S**	OTUp_228	Bacteria	Proteobacteria	Alphaproteobacteria	Sphingomonadales	Sphingomonadaceae	Sphingomonas	unclassified	-0.534	-0.511	0.756	0.289	0	0	1	1	0.90	0.001
**Arctic-S**	OTUp_235	Bacteria	Actinobacteria	Acidimicrobiia	Acidimicrobiales	Acidimicrobiaceae	CL500-29_marine_group	uncultured_bacterium	-0.497	-0.419	0.219	0.696	0	0	1	1	0.79	0.005
**Arctic-S**	OTUp_259	Bacteria	Bacteroidetes	Sphingobacteriia	Sphingobacteriales	Chitinophagaceae	uncultured	uncultured_soil_bacterium	-0.114	-0.670	0.164	0.621	0	0	1	1	0.68	0.013
**Arctic-S**	OTUp_285	Bacteria	Verrucomicrobia	Spartobacteria	Chthoniobacterales	DA101_soil_group	uncultured_Verrucomicrobia_bacterium	unclassified	-0.539	-0.198	0.107	0.630	0	0	1	1	0.64	0.050
**Arctic-S**	OTUp_301	Bacteria	Actinobacteria	Actinobacteria	Frankiales	Frankiaceae	Jatrophihabitans	uncultured_bacterium	-0.397	-0.175	0.683	-0.112	0	0	1	0	0.68	0.024
**Arctic-S**	OTUp_302	Bacteria	Nitrospirae	Nitrospira	Nitrospirales	Nitrospiraceae	Nitrospira	uncultured_bacterium	-0.401	-0.351	0.554	0.198	0	0	1	1	0.65	0.048
**Arctic-S**	OTUp_314	Bacteria	Planctomycetes	Planctomycetacia	Planctomycetales	Planctomycetaceae	unclassified	unclassified	-0.331	-0.257	-0.202	0.789	0	0	0	1	0.79	0.008
**Arctic-S**	OTUp_320	Bacteria	Bacteroidetes	Sphingobacteriia	Sphingobacteriales	Sphingobacteriaceae	Mucilaginibacter	uncultured_Bacteroidetes_bacterium	-0.482	0.529	-0.225	0.178	0	1	0	1	0.61	0.045
**Arctic-S**	OTUp_325	Bacteria	Acidobacteria	Holophagae	Subgroup_10	ABS-19	uncultured_bacterium	unclassified	-0.391	-0.463	0.467	0.387	0	0	1	1	0.74	0.007
**Arctic-S**	OTUp_331	Bacteria	Actinobacteria	Actinobacteria	Frankiales	Sporichthyaceae	uncultured	unclassified	-0.417	-0.070	0.730	-0.244	0	0	1	0	0.73	0.008
**Arctic-S**	OTUp_354	Bacteria	Planctomycetes	Planctomycetacia	Planctomycetales	Planctomycetaceae	uncultured	unclassified	-0.457	0.681	-0.347	0.124	0	1	0	1	0.70	0.023
**Arctic-S**	OTUp_362	Bacteria	Acidobacteria	Holophagae	Subgroup_7	uncultured_Acidobacteria_bacterium	unclassified	unclassified	-0.286	-0.286	-0.093	0.665	0	0	0	1	0.66	0.028
**Arctic-S**	OTUp_366	Bacteria	Armatimonadetes	uncultured_bacterium	unclassified	unclassified	unclassified	unclassified	-0.642	0.589	-0.313	0.366	0	1	0	1	0.83	0.001
**Arctic-S**	OTUp_385	Bacteria	Verrucomicrobia	Spartobacteria	Chthoniobacterales	DA101_soil_group	uncultured_Verrucomicrobia_bacterium	unclassified	-0.418	-0.527	0.534	0.411	0	0	1	1	0.82	0.001
**Arctic-S**	OTUp_391	Bacteria	Acidobacteria	Acidobacteria	Subgroup_6	unclassified	unclassified	unclassified	-0.456	-0.344	0.175	0.625	0	0	1	1	0.69	0.023
**Arctic-S**	OTUp_395	Bacteria	Actinobacteria	Actinobacteria	Micrococcales	Intrasporangiaceae	Janibacter	uncultured_bacterium	-0.149	-0.535	0.716	-0.032	0	0	1	0	0.72	0.011
**Arctic-S**	OTUp_406	Bacteria	Chloroflexi	Anaerolineae	Anaerolineales	Anaerolineaceae	uncultured	unclassified	-0.656	-0.151	0.405	0.403	0	0	1	1	0.70	0.003
**Arctic-S**	OTUp_422	Bacteria	Verrucomicrobia	Spartobacteria	Chthoniobacterales	Chthoniobacteraceae	Chthoniobacter	bacterium_Ellin506	-0.432	-0.374	0.397	0.409	0	0	1	1	0.70	0.016
**Arctic-S**	OTUp_424	Bacteria	Proteobacteria	Betaproteobacteria	Burkholderiales	unclassified	unclassified	unclassified	-0.400	-0.416	0.385	0.431	0	0	1	1	0.71	0.023
**Arctic-S**	OTUp_441	Bacteria	Chloroflexi	KD4-96	uncultured_Chloroflexi_bacterium	unclassified	unclassified	unclassified	-0.193	-0.353	0.716	-0.171	0	0	1	0	0.72	0.015
**Arctic-S**	OTUp_445	Bacteria	Proteobacteria	Alphaproteobacteria	Rhizobiales	A0839	unclassified	unclassified	-0.446	-0.291	0.012	0.725	0	0	0	1	0.72	0.007
**Arctic-S**	OTUp_469	Bacteria	Proteobacteria	Betaproteobacteria	Nitrosomonadales	Nitrosomonadaceae	uncultured	unclassified	-0.453	-0.433	0.293	0.593	0	0	1	1	0.77	0.007
**Arctic-S**	OTUp_477	Bacteria	Acidobacteria	Acidobacteria	Subgroup_2	bacterium_enrichment_culture_clone_heteroC52_4W	unclassified	unclassified	-0.704	0.154	-0.068	0.617	0	1	1	1	0.70	0.024
**Arctic-S**	OTUp_479	Bacteria	Chloroflexi	Anaerolineae	Anaerolineales	Anaerolineaceae	uncultured	uncultured_Gemmatimonadetes_bacterium	-0.274	-0.152	-0.233	0.659	0	0	0	1	0.66	0.040
**Arctic-S**	OTUp_537	Bacteria	Verrucomicrobia	Opitutae	Opitutales	Opitutaceae	Opitutus	uncultured_bacterium	-0.601	-0.138	0.327	0.412	0	0	1	1	0.64	0.046
**Arctic-S**	OTUp_552	Bacteria	Verrucomicrobia	Spartobacteria	Chthoniobacterales	01D2Z36	uncultured_cyanobacterium	unclassified	-0.520	-0.441	0.252	0.709	0	0	1	1	0.83	0.001
**Arctic-S**	OTUp_564	Bacteria	Chloroflexi	Anaerolineae	Anaerolineales	Anaerolineaceae	uncultured	uncultured_soil_bacterium	-0.533	-0.350	0.523	0.359	0	0	1	1	0.76	0.013
**Arctic-S**	OTUp_572	Bacteria	Planctomycetes	Planctomycetacia	Planctomycetales	Planctomycetaceae	uncultured	uncultured_bacterium	-0.710	0.449	0.286	-0.025	0	1	1	1	0.71	0.016
**Arctic-S**	OTUp_582	Bacteria	Acidobacteria	Acidobacteria	Subgroup_6	uncultured_bacterium_270	unclassified	unclassified	-0.376	-0.331	-0.090	0.797	0	0	0	1	0.80	0.001
**Arctic-S**	OTUp_586	Bacteria	Proteobacteria	Betaproteobacteria	unclassified	unclassified	unclassified	unclassified	-0.481	-0.503	0.233	0.751	0	0	1	1	0.85	0.001
**Arctic-S**	OTUp_597	Bacteria	Acidobacteria	Acidobacteria	Acidobacteriales	Acidobacteriaceae	uncultured	uncultured_Acidobacteriaceae_bacterium	-0.320	-0.367	-0.123	0.809	0	0	0	1	0.81	0.004
**Arctic-S**	OTUp_611	Bacteria	Actinobacteria	Actinobacteria	Pseudonocardiales	Pseudonocardiaceae	Pseudonocardia	uncultured_bacterium	-0.344	-0.414	0.585	0.174	0	0	1	1	0.66	0.032
**Arctic-S**	OTUp_622	Bacteria	Proteobacteria	Betaproteobacteria	unclassified	unclassified	unclassified	unclassified	-0.351	-0.147	-0.211	0.710	0	0	0	1	0.71	0.022
**Arctic-S**	OTUp_628	Bacteria	Acidobacteria	Acidobacteria	Subgroup_6	unclassified	unclassified	unclassified	-0.218	-0.582	0.615	0.184	0	0	1	1	0.69	0.016
**Arctic-S**	OTUp_633	Bacteria	Planctomycetes	Planctomycetacia	Planctomycetales	Planctomycetaceae	uncultured	uncultured_Zavarzinella_sp.	-0.295	-0.439	0.390	0.344	0	0	1	1	0.64	0.043
**Arctic-S**	OTUp_646	Bacteria	Acidobacteria	Acidobacteria	Acidobacteriales	Acidobacteriaceae	Candidatus_Koribacter	uncultured_Acidobacteria_bacterium	-0.532	0.403	-0.208	0.337	0	1	0	1	0.64	0.042
**Arctic-S**	OTUp_666	Bacteria	Chloroflexi	JG30-KF-CM66	uncultured_bacterium	unclassified	unclassified	unclassified	-0.326	-0.451	0.342	0.436	0	0	1	1	0.67	0.036
**Arctic-S**	OTUp_669	Bacteria	Chloroflexi	Chloroflexia	Chloroflexales	Roseiflexaceae	Roseiflexus	uncultured_bacterium	-0.313	-0.534	0.479	0.368	0	0	1	1	0.73	0.016
**Arctic-S**	OTUp_691	Bacteria	Verrucomicrobia	Spartobacteria	Chthoniobacterales	Chthoniobacteraceae	Chthoniobacter	uncultured_Verrucomicrobia_bacterium	-0.472	-0.360	0.147	0.685	0	0	1	1	0.72	0.010
**Arctic-S**	OTUp_704	Bacteria	Planctomycetes	Phycisphaerae	WD2101_soil_group	uncultured_bacterium	unclassified	unclassified	-0.392	-0.269	-0.125	0.786	0	0	0	1	0.79	0.003
**Arctic-S**	OTUp_711	Bacteria	Acidobacteria	Acidobacteria	Acidobacteriales	Acidobacteriaceae	uncultured	uncultured_bacterium	-0.412	-0.309	-0.056	0.777	0	0	0	1	0.78	0.007

**Arctic-S**	OTUp_720	Bacteria	Verrucomicrobia	Spartobacteria	Chthoniobacterales	DA101_soil_group	uncultured_Verrucomicrobia_bacterium	unclassified	-0.420	-0.392	0.192	0.620	0	0	1	1	0.70	0.030
**Arctic-S**	OTUp_742	Bacteria	WD272	uncultured_Firmicutes_bacterium	unclassified	unclassified	unclassified	unclassified	-0.650	0.045	0.001	0.604	0	1	1	1	0.65	0.041
**Arctic-S**	OTUp_752	Bacteria	Proteobacteria	Betaproteobacteria	Nitrosomonadales	Nitrosomonadaceae	uncultured	uncultured_Burkholderiaceae_bacterium	-0.304	-0.130	-0.209	0.643	0	0	0	1	0.64	0.044
**Arctic-S**	OTUp_756	Bacteria	Proteobacteria	Betaproteobacteria	Burkholderiales	Comamonadaceae	unclassified	unclassified	-0.152	-0.608	0.608	0.152	0	0	1	1	0.66	0.044
**Arctic-S**	OTUp_773	Bacteria	Proteobacteria	Gammaproteobacteria	Xanthomonadales	Xanthomonadaceae	uncultured	unclassified	-0.685	-0.233	0.369	0.549	0	0	1	1	0.79	0.002
**Arctic-S**	OTUp_787	Bacteria	Proteobacteria	Betaproteobacteria	SC-I-84	uncultured_beta_proteobacterium	unclassified	unclassified	-0.268	-0.506	-0.006	0.780	0	0	0	1	0.78	0.005
**Arctic-S**	OTUp_797	Bacteria	Acidobacteria	Acidobacteria	Subgroup_3	Unknown_Family	Candidatus_Solibacter	uncultured_Acidobacteria_bacterium	-0.611	-0.407	0.525	0.492	0	0	1	1	0.88	0.000
**Arctic-S**	OTUp_803	Bacteria	Actinobacteria	Acidimicrobiia	Acidimicrobiales	unclassified	unclassified	unclassified	-0.370	-0.233	0.702	-0.098	0	0	1	0	0.70	0.017
**Arctic-S**	OTUp_806	Bacteria	Nitrospirae	Nitrospira	Nitrospirales	0319-6A21	uncultured_bacterium	unclassified	-0.228	-0.483	0.006	0.705	0	0	0	1	0.71	0.023
**Arctic-S**	OTUp_807	Bacteria	unclassified	unclassified	unclassified	unclassified	unclassified	unclassified	-0.827	0.166	0.159	0.502	0	1	1	1	0.83	0.002
**Arctic-S**	OTUp_842	Bacteria	Verrucomicrobia	Spartobacteria	Chthoniobacterales	DA101_soil_group	uncultured_Xiphinematobacteriaceae_bacterium	unclassified	-0.354	-0.388	0.134	0.609	0	0	1	1	0.64	0.045
**Arctic-S**	OTUp_845	Bacteria	Planctomycetes	Phycisphaerae	WD2101_soil_group	uncultured_bacterium	unclassified	unclassified	-0.372	-0.398	0.199	0.571	0	0	1	1	0.67	0.038
**Arctic-S**	OTUp_848	Bacteria	Proteobacteria	Alphaproteobacteria	Caulobacterales	Caulobacteraceae	Phenylobacterium	unclassified	-0.514	-0.363	0.498	0.379	0	0	1	1	0.76	0.009
**Arctic-S**	OTUp_908	Bacteria	Verrucomicrobia	Spartobacteria	Chthoniobacterales	DA101_soil_group	unclassified	unclassified	0.016	-0.495	-0.266	0.745	0	0	0	1	0.74	0.016
**Arctic-S**	OTUp_911	Bacteria	Verrucomicrobia	Spartobacteria	Chthoniobacterales	Chthoniobacteraceae	Chthoniobacter	uncultured_bacterium	-0.301	-0.433	0.048	0.685	0	0	0	1	0.69	0.026
**Arctic-S**	OTUp_934	Bacteria	Actinobacteria	Acidimicrobiia	Acidimicrobiales	uncultured	uncultured_Iamiaceae_bacterium	unclassified	-0.587	-0.210	0.303	0.493	0	0	1	1	0.69	0.022
**Arctic-S**	OTUp_935	Bacteria	Proteobacteria	Betaproteobacteria	Nitrosomonadales	Nitrosomonadaceae	uncultured	uncultured_Nitrosomonadaceae_bacterium	-0.359	-0.440	0.225	0.573	0	0	1	1	0.69	0.025
**Arctic-S**	OTUp_952	Bacteria	SM2F11	uncultured_bacterium	unclassified	unclassified	unclassified	unclassified	-0.162	-0.449	-0.266	0.876	0	0	0	1	0.88	0.002
**Arctic-S**	OTUp_1001	Bacteria	Bacteroidetes	Sphingobacteriia	Sphingobacteriales	Sphingobacteriaceae	Mucilaginibacter	uncultured_Mucilaginibacter_sp.	-0.342	-0.227	-0.155	0.724	0	0	0	1	0.72	0.006
**Arctic-S**	OTUp_1016	Bacteria	Actinobacteria	Acidimicrobiia	Acidimicrobiales	uncultured	unclassified	unclassified	-0.339	-0.417	0.165	0.591	0	0	1	1	0.66	0.035
**Arctic-S**	OTUp_1022	Bacteria	Bacteroidetes	Sphingobacteriia	Sphingobacteriales	Chitinophagaceae	Terrimonas	uncultured_bacterium	-0.351	-0.229	-0.040	0.620	0	0	0	1	0.62	0.047
**Arctic-S**	OTUp_1031	Bacteria	Actinobacteria	Acidimicrobiia	Acidimicrobiales	Acidimicrobiaceae	CL500-29_marine_group	unclassified	-0.394	-0.449	0.421	0.423	0	0	1	1	0.73	0.011
**Arctic-S**	OTUp_1070	Bacteria	Planctomycetes	Planctomycetacia	Planctomycetales	Planctomycetaceae	uncultured	unclassified	-0.587	-0.130	0.048	0.669	0	0	0	1	0.67	0.034
**Arctic-S**	OTUp_1091	Bacteria	Actinobacteria	Acidimicrobiia	Acidimicrobiales	uncultured	unclassified	unclassified	-0.196	-0.566	0.497	0.265	0	0	1	1	0.66	0.039
**Arctic-S**	OTUp_1103	Bacteria	Bacteroidetes	Sphingobacteriia	Sphingobacteriales	Chitinophagaceae	uncultured	uncultured_Chitinophagaceae_bacterium	-0.417	-0.436	0.445	0.408	0	0	1	1	0.74	0.009
**Arctic-S**	OTUp_1112	Bacteria	Proteobacteria	Betaproteobacteria	SC-I-84	uncultured_Comamonadaceae_bacterium	unclassified	unclassified	-0.476	-0.569	0.307	0.737	0	0	1	1	0.90	0.000
**Arctic-S**	OTUp_1113	Bacteria	Planctomycetes	Planctomycetacia	Planctomycetales	Planctomycetaceae	uncultured	uncultured_planctomycete	-0.589	0.638	0.173	-0.222	0	1	1	0	0.70	0.022
**Arctic-S**	OTUp_1163	Bacteria	Planctomycetes	Planctomycetacia	Planctomycetales	Planctomycetaceae	uncultured	uncultured_bacterium	-0.500	-0.353	0.249	0.604	0	0	1	1	0.74	0.005
**Arctic-S**	OTUp_1236	Bacteria	Chloroflexi	Anaerolineae	Anaerolineales	Anaerolineaceae	uncultured	uncultured_sludge_bacterium_S14	-0.455	-0.418	0.195	0.678	0	0	1	1	0.76	0.011
**Arctic-S**	OTUp_1261	Bacteria	Acidobacteria	Acidobacteria	Subgroup_3	Unknown_Family	Bryobacter	uncultured_bacterium	-0.260	-0.559	0.293	0.525	0	0	1	1	0.71	0.017
**Arctic-S**	OTUp_1266	Bacteria	Planctomycetes	Phycisphaerae	WD2101_soil_group	uncultured_bacterium	unclassified	unclassified	-0.440	-0.267	0.012	0.695	0	0	0	1	0.69	0.022
**Arctic-S**	OTUp_1299	Bacteria	Acidobacteria	Acidobacteria	Subgroup_4	11-24	Acidobacteria_bacterium_Ellin7246	unclassified	-0.590	0.285	-0.197	0.501	0	1	0	1	0.68	0.024
**Arctic-S**	OTUp_1309	Bacteria	Microgenomates	uncultured_bacterium	unclassified	unclassified	unclassified	unclassified	-0.607	-0.131	0.496	0.242	0	0	1	1	0.64	0.048
**Arctic-S**	OTUp_1348	Bacteria	Proteobacteria	Betaproteobacteria	Nitrosomonadales	Nitrosomonadaceae	uncultured	uncultured_Oxalobacteraceae_bacterium	-0.336	-0.554	0.569	0.321	0	0	1	1	0.77	0.004
**Arctic-S**	OTUp_1389	Bacteria	Acidobacteria	Acidobacteria	Subgroup_4	RB41	uncultured_Acidobacteria_bacterium	unclassified	-0.470	-0.386	0.109	0.747	0	0	0	1	0.75	0.011
**Arctic-S**	OTUp_1419	Bacteria	Verrucomicrobia	Spartobacteria	Chthoniobacterales	Chthoniobacteraceae	Chthoniobacter	uncultured_bacterium	-0.138	-0.420	-0.093	0.651	0	0	0	1	0.65	0.040
**Arctic-S**	OTUp_1423	Bacteria	Planctomycetes	Planctomycetacia	Planctomycetales	Planctomycetaceae	Gemmata	uncultured_bacterium	-0.307	-0.491	0.095	0.702	0	0	0	1	0.70	0.017
**Arctic-S**	OTUp_1424	Bacteria	Acidobacteria	Holophagae	Subgroup_7	uncultured_proteobacterium	unclassified	unclassified	-0.388	-0.480	0.319	0.549	0	0	1	1	0.75	0.009
**Arctic-S**	OTUp_1430	Bacteria	Planctomycetes	Planctomycetacia	Planctomycetales	Planctomycetaceae	Telmatocola	uncultured_planctomycete	-0.397	-0.174	0.691	-0.120	0	0	1	0	0.69	0.037
**Arctic-S**	OTUp_1437	Bacteria	Proteobacteria	Betaproteobacteria	Nitrosomonadales	Nitrosomonadaceae	uncultured_alpha_proteobacterium	unclassified	-0.004	-0.452	-0.187	0.643	0	0	0	1	0.64	0.036
**Arctic-S**	OTUp_1478	Bacteria	Proteobacteria	Betaproteobacteria	SC-I-84	unclassified	unclassified	unclassified	-0.410	-0.476	0.114	0.771	0	0	0	1	0.77	0.008
**Arctic-S**	OTUp_1481	Bacteria	Planctomycetes	Planctomycetacia	Planctomycetales	Planctomycetaceae	Gemmata	uncultured_bacterium	-0.401	-0.212	-0.166	0.779	0	0	0	1	0.78	0.005
**Arctic-S**	OTUp_1492	Bacteria	Acidobacteria	Acidobacteria	Subgroup_2	uncultured_forest_soil_bacterium	unclassified	unclassified	-0.499	0.084	0.744	-0.330	0	0	1	0	0.74	0.012
**Arctic-S**	OTUp_1498	Bacteria	Chloroflexi	Anaerolineae	Anaerolineales	Anaerolineaceae	uncultured	uncultured_Gemmatimonadetes_bacterium	-0.352	-0.461	0.393	0.421	0	0	1	1	0.70	0.029
**Arctic-S**	OTUp_1545	Bacteria	Bacteroidetes	Sphingobacteriia	Sphingobacteriales	Chitinophagaceae	uncultured	uncultured_bacterium	-0.410	-0.227	-0.207	0.844	0	0	0	1	0.84	0.002
**Arctic-S**	OTUp_1569	Bacteria	Proteobacteria	Betaproteobacteria	Burkholderiales	Comamonadaceae	unclassified	unclassified	-0.148	-0.710	0.546	0.312	0	0	1	1	0.74	0.009
**Arctic-S**	OTUp_1589	Bacteria	Verrucomicrobia	Verrucomicrobiae	Verrucomicrobiales	Verrucomicrobiaceae	Luteolibacter	uncultured_bacterium	-0.177	-0.444	0.728	-0.108	0	0	1	0	0.73	0.038
**Arctic-S**	OTUp_1602	Bacteria	Verrucomicrobia	Spartobacteria	Chthoniobacterales	DA101_soil_group	uncultured_bacterium	unclassified	-0.388	0.394	-0.576	0.571	0	1	0	1	0.84	0.001
**Arctic-S**	OTUp_1605	Bacteria	Elusimicrobia	Elusimicrobia	Lineage_IIa	uncultured_bacterium	unclassified	unclassified	-0.284	-0.210	-0.128	0.622	0	0	0	1	0.62	0.037
**Arctic-S**	OTUp_1690	Bacteria	Chloroflexi	unclassified	unclassified	unclassified	unclassified	unclassified	-0.314	-0.314	0.729	-0.102	0	0	1	0	0.73	0.028
**Arctic-S**	OTUp_1753	Bacteria	Proteobacteria	Gammaproteobacteria	Xanthomonadales	uncultured	unclassified	unclassified	-0.328	-0.574	0.206	0.696	0	0	1	1	0.78	0.005
**Arctic-S**	OTUp_1754	Bacteria	Actinobacteria	Actinobacteria	Frankiales	Sporichthyaceae	Candidatus_Planktophila	uncultured_Sporichthyaceae_bacterium	-0.235	-0.081	-0.405	0.720	0	0	0	1	0.72	0.016
**Arctic-S**	OTUp_1770	Bacteria	Saccharibacteria	uncultured_Candidatus_Saccharibacteria	unclassified	unclassified	unclassified	unclassified	-0.164	-0.591	0.268	0.487	0	0	1	1	0.65	0.045
**Arctic-S**	OTUp_1820	Bacteria	Bacteroidetes	Sphingobacteriia	Sphingobacteriales	Chitinophagaceae	Ferruginibacter	unclassified	-0.398	-0.511	0.547	0.363	0	0	1	1	0.79	0.005
**Arctic-S**	OTUp_1849	Bacteria	Parcubacteria	uncultured_bacterium	unclassified	unclassified	unclassified	unclassified	-0.474	0.716	-0.373	0.131	0	1	0	1	0.73	0.006
**Arctic-S**	OTUp_1881	Bacteria	Chloroflexi	Anaerolineae	Anaerolineales	Anaerolineaceae	uncultured	uncultured_Gemmatimonadetes_bacterium	-0.414	-0.235	-0.050	0.698	0	0	0	1	0.70	0.007
**Arctic-S**	OTUp_1886	Bacteria	Planctomycetes	Planctomycetacia	Planctomycetales	Planctomycetaceae	uncultured	uncultured_bacterium	-0.372	-0.425	0.584	0.213	0	0	1	1	0.69	0.022
**Arctic-S**	OTUp_1894	Bacteria	Bacteroidetes	Cytophagia	Cytophagales	Cytophagaceae	uncultured	uncultured_Cytophagaceae_bacterium	-0.331	-0.331	-0.051	0.713	0	0	0	1	0.71	0.028
**Arctic-S**	OTUp_1906	Bacteria	Proteobacteria	Betaproteobacteria	Nitrosomonadales	Nitrosomonadaceae	uncultured	uncultured_beta_proteobacterium	-0.302	-0.508	0.025	0.785	0	0	0	1	0.79	0.004
**Arctic-S**	OTUp_1925	Bacteria	Chloroflexi	Ktedonobacteria	B12-WMSP1	uncultured_Chloroflexi_bacterium	unclassified	unclassified	-0.607	0.458	-0.181	0.329	0	1	0	1	0.68	0.021
**Arctic-S**	OTUp_1943	Bacteria	Chloroflexi	unclassified	unclassified	unclassified	unclassified	unclassified	-0.487	-0.487	0.673	0.302	0	0	1	1	0.84	0.003
**Arctic-S**	OTUp_1946	Bacteria	Actinobacteria	Acidimicrobiia	Acidimicrobiales	uncultured	Acidimicrobidae_bacterium_Ellin7143	unclassified	-0.098	-0.247	0.697	-0.352	0	0	1	0	0.70	0.019
**Arctic-S**	OTUp_2069	Bacteria	Acidobacteria	Acidobacteria	Subgroup_4	Unknown_Family	Blastocatella	uncultured_Acidobacteria_bacterium	-0.409	-0.406	0.337	0.479	0	0	1	1	0.71	0.024
**Arctic-S**	OTUp_2099	Bacteria	Proteobacteria	Betaproteobacteria	SC-I-84	uncultured_Burkholderiaceae_bacterium	unclassified	unclassified	-0.601	-0.469	0.374	0.696	0	0	1	1	0.93	0.001
**Arctic-S**	OTUp_2103	Bacteria	Proteobacteria	Alphaproteobacteria	Rhodospirillales	Acetobacteraceae	unclassified	unclassified	-0.177	-0.268	0.713	-0.268	0	0	1	0	0.71	0.018
**Arctic-S**	OTUp_2123	Bacteria	Actinobacteria	Actinobacteria	Micromonosporales	Micromonosporaceae	Luedemannella	uncultured_bacterium	-0.307	-0.589	0.349	0.547	0	0	1	1	0.78	0.006
**Arctic-S**	OTUp_2135	Bacteria	TM6	uncultured_soil_bacterium	unclassified	unclassified	unclassified	unclassified	-0.356	-0.256	-0.043	0.655	0	0	0	1	0.66	0.040
**Arctic-S**	OTUp_2171	Bacteria	Gemmatimonadetes	Gemmatimonadetes	Gemmatimonadales	Gemmatimonadaceae	Gemmatimonas	unclassified	-0.352	-0.515	0.269	0.598	0	0	1	1	0.75	0.009
**Arctic-S**	OTUp_2203	Bacteria	Chloroflexi	KD4-96	uncultured_bacterium	unclassified	unclassified	unclassified	-0.189	-0.305	0.624	-0.130	0	0	1	0	0.62	0.045
**Arctic-S**	OTUp_2234	Bacteria	Acidobacteria	Acidobacteria	Subgroup_4	Unknown_Family	Blastocatella	uncultured_Acidobacteria_bacterium	-0.502	-0.033	-0.205	0.739	0	0	0	1	0.74	0.007
**Arctic-S**	OTUp_2291	Bacteria	Firmicutes	Erysipelotrichia	Erysipelotrichales	Erysipelotrichaceae	Asteroleplasma	uncultured_bacterium	0.047	-0.364	0.667	-0.350	0	0	1	0	0.67	0.032
**Arctic-S**	OTUp_2439	Bacteria	Acidobacteria	Holophagae	Subgroup_7	unclassified	unclassified	unclassified	-0.306	-0.493	-0.054	0.853	0	0	0	1	0.85	0.002
**Arctic-S**	OTUp_2448	Bacteria	Planctomycetes	Planctomycetacia	Planctomycetales	Planctomycetaceae	uncultured	uncultured_bacterium	-0.266	-0.237	0.800	-0.297	0	0	1	0	0.80	0.005
**Arctic-S**	OTUp_2529	Bacteria	Acidobacteria	Holophagae	Subgroup_7	uncultured_bacterium	unclassified	unclassified	-0.267	-0.533	0.302	0.498	0	0	1	1	0.69	0.023
**Arctic-S**	OTUp_2541	Bacteria	Parcubacteria	uncultured_bacterium	unclassified	unclassified	unclassified	unclassified	-0.141	-0.371	-0.137	0.649	0	0	0	1	0.65	0.041
**Arctic-S**	OTUp_2594	Bacteria	Microgenomates	uncultured_Chloroflexi_bacterium	unclassified	unclassified	unclassified	unclassified	-0.324	-0.227	0.791	-0.240	0	0	1	0	0.79	0.004
**Arctic-S**	OTUp_2629	Bacteria	Chloroflexi	Anaerolineae	Anaerolineales	Anaerolineaceae	uncultured	uncultured_Gemmatimonadetes_bacterium	-0.431	-0.412	0.490	0.354	0	0	1	1	0.73	0.017
**Arctic-S**	OTUp_2651	Bacteria	Verrucomicrobia	Spartobacteria	Chthoniobacterales	DA101_soil_group	unclassified	unclassified	-0.467	0.523	-0.467	0.410	0	1	0	1	0.81	0.004
**Arctic-S**	OTUp_2741	Bacteria	Parcubacteria	unclassified	unclassified	unclassified	unclassified	unclassified	-0.370	-0.017	-0.370	0.756	0	0	0	1	0.76	0.007
**Arctic-S**	OTUp_2745	Bacteria	Acidobacteria	Acidobacteria	Acidobacteriales	Acidobacteriaceae	uncultured	unclassified	-0.277	-0.574	0.105	0.746	0	0	0	1	0.75	0.006
**Arctic-S**	OTUp_2754	Bacteria	Proteobacteria	Deltaproteobacteria	Myxococcales	Haliangiaceae	Haliangium	uncultured_bacterium	-0.294	-0.050	0.625	-0.281	0	0	1	0	0.62	0.021
**Arctic-S**	OTUp_2772	Bacteria	Bacteroidetes	Sphingobacteriia	Sphingobacteriales	NS11-12_marine_group	uncultured_bacterium	unclassified	-0.227	-0.409	0.645	-0.009	0	0	1	0	0.64	0.050
**Arctic-S**	OTUp_2811	Bacteria	Acidobacteria	Acidobacteria	Subgroup_6	uncultured_Acidobacteria_bacterium	unclassified	unclassified	-0.263	-0.167	-0.280	0.710	0	0	0	1	0.71	0.012
**Arctic-S**	OTUp_2833	Bacteria	Proteobacteria	Betaproteobacteria	Burkholderiales	unclassified	unclassified	unclassified	-0.242	-0.611	0.802	0.051	0	0	1	0	0.80	0.005
**Arctic-S**	OTUp_2884	Bacteria	Bacteroidetes	Sphingobacteriia	Sphingobacteriales	Chitinophagaceae	uncultured	unclassified	-0.262	-0.262	-0.262	0.786	0	0	0	1	0.79	0.027
**Arctic-S**	OTUp_2920	Bacteria	Planctomycetes	Phycisphaerae	WD2101_soil_group	uncultured_bacterium	unclassified	unclassified	-0.457	-0.221	0.682	-0.004	0	0	1	0	0.68	0.040
**Arctic-S**	OTUp_2979	Bacteria	Chloroflexi	JG37-AG-4	uncultured_Chloroflexi_bacterium	unclassified	unclassified	unclassified	-0.038	-0.315	0.668	-0.315	0	0	1	0	0.67	0.030
**Arctic-S**	OTUp_2983	Bacteria	WCHB1-60	uncultured_bacterium	unclassified	unclassified	unclassified	unclassified	-0.454	0.401	-0.347	0.399	0	1	0	1	0.69	0.025
**Arctic-S**	OTUp_3007	Bacteria	Bacteroidetes	Sphingobacteriia	Sphingobacteriales	Chitinophagaceae	Ferruginibacter	uncultured_bacterium	-0.380	-0.282	-0.067	0.730	0	0	0	1	0.73	0.014
**Arctic-S**	OTUp_3042	Bacteria	Bacteroidetes	Sphingobacteriia	Sphingobacteriales	Chitinophagaceae	Sediminibacterium	uncultured_bacterium	-0.383	-0.249	0.783	-0.151	0	0	1	0	0.78	0.007
**Arctic-S**	OTUp_3087	Bacteria	Proteobacteria	Alphaproteobacteria	Rhodospirillales	Rhodospirillales_Incertae_Sedis	Reyranella	uncultured_bacterium	-0.674	0.169	0.444	0.061	0	1	1	1	0.67	0.031
**Arctic-S**	OTUp_3094	Bacteria	Bacteroidetes	Sphingobacteriia	Sphingobacteriales	Chitinophagaceae	uncultured	uncultured_bacterium	-0.307	-0.404	0.055	0.657	0	0	0	1	0.66	0.036
**Arctic-S**	OTUp_3118	Bacteria	Chloroflexi	Anaerolineae	Anaerolineales	Anaerolineaceae	uncultured	uncultured_Gemmatimonadetes_bacterium	-0.008	-0.454	0.701	-0.239	0	0	1	0	0.70	0.023
**Arctic-S**	OTUp_3279	Bacteria	Actinobacteria	Acidimicrobiia	Acidimicrobiales	uncultured	uncultured_bacterium	unclassified	-0.498	-0.327	0.306	0.519	0	0	1	1	0.71	0.020
**Arctic-S**	OTUp_3361	unknown	unclassified	unclassified	unclassified	unclassified	unclassified	unclassified	-0.405	-0.281	-0.022	0.707	0	0	0	1	0.71	0.033
**Arctic-S**	OTUp_3380	Bacteria	Proteobacteria	Deltaproteobacteria	GR-WP33-30	unclassified	unclassified	unclassified	-0.494	0.414	0.417	-0.337	0	1	1	0	0.72	0.021

**Arctic-S**	OTUp_3421	Bacteria	Microgenomates	uncultured_bacterium	unclassified	unclassified	unclassified	unclassified	-0.347	0.010	-0.324	0.661	0	0	0	1	0.66	0.041
**Arctic-S**	OTUp_3454	Bacteria	Armatimonadetes	uncultured_Carnobacterium_sp.	unclassified	unclassified	unclassified	unclassified	-0.306	-0.200	0.661	-0.155	0	0	1	0	0.66	0.044
**Arctic-S**	OTUp_3484	Bacteria	Proteobacteria	Gammaproteobacteria	Xanthomonadales	Xanthomonadaceae	Dokdonella	uncultured_Dokdonella_sp.	-0.365	-0.431	0.199	0.596	0	0	1	1	0.69	0.026
**Arctic-S**	OTUp_3507	Bacteria	Chloroflexi	Thermomicrobia	JG30-KF-CM45	unclassified	unclassified	unclassified	-0.431	0.676	0.148	-0.394	0	1	1	0	0.71	0.017
**Arctic-S**	OTUp_3523	Bacteria	Acidobacteria	Acidobacteria	Subgroup_5	uncultured_Acidobacteria_bacterium	unclassified	unclassified	-0.266	-0.509	0.597	0.178	0	0	1	1	0.67	0.030
**Arctic-S**	OTUp_3555	Bacteria	Proteobacteria	Deltaproteobacteria	Myxococcales	Haliangiaceae	Haliangium	unclassified	-0.357	-0.357	0.234	0.481	0	0	1	1	0.62	0.050
**Arctic-S**	OTUp_3603	Bacteria	Gemmatimonadetes	Gemmatimonadetes	Gemmatimonadales	Gemmatimonadaceae	Gemmatimonas	uncultured_bacterium	-0.491	-0.466	0.427	0.529	0	0	1	1	0.83	0.001
**Arctic-S**	OTUp_3631	Bacteria	Acidobacteria	Acidobacteria	Subgroup_6	unclassified	unclassified	unclassified	-0.368	-0.361	0.020	0.709	0	0	0	1	0.71	0.019
**Arctic-S**	OTUp_3639	Bacteria	Acidobacteria	Holophagae	Subgroup_7	uncultured_Acidobacteria_bacterium	unclassified	unclassified	-0.044	-0.496	-0.166	0.706	0	0	0	1	0.71	0.019
**Arctic-S**	OTUp_3656	Bacteria	Firmicutes	Erysipelotrichia	Erysipelotrichales	Erysipelotrichaceae	Asteroleplasma	uncultured_bacterium	-0.345	-0.160	-0.139	0.644	0	0	0	1	0.64	0.034
**Arctic-S**	OTUp_3659	Bacteria	Proteobacteria	Deltaproteobacteria	Myxococcales	Haliangiaceae	Haliangium	unclassified	-0.243	-0.283	-0.108	0.634	0	0	0	1	0.63	0.046
**Arctic-S**	OTUp_3669	Bacteria	Bacteroidetes	Sphingobacteriia	Sphingobacteriales	Saprospiraceae	uncultured	uncultured_bacterium_PHOS-HE79	-0.311	-0.311	-0.311	0.934	0	0	0	1	0.93	0.002
**Arctic-S**	OTUp_3675	Bacteria	Bacteroidetes	Sphingobacteriia	Sphingobacteriales	Chitinophagaceae	Terrimonas	uncultured_Bacteroidetes_bacterium	-0.281	-0.542	0.416	0.408	0	0	1	1	0.71	0.012
**Arctic-S**	OTUp_3712	Bacteria	Actinobacteria	Acidimicrobiia	Acidimicrobiales	uncultured	uncultured_bacterium	unclassified	-0.228	-0.299	0.775	-0.248	0	0	1	0	0.78	0.013
**Arctic-S**	OTUp_3724	Bacteria	Verrucomicrobia	Spartobacteria	Chthoniobacterales	DA101_soil_group	uncultured_Verrucomicrobia_bacterium	unclassified	-0.242	-0.242	-0.242	0.727	0	0	0	1	0.73	0.029
**Arctic-S**	OTUp_3858	Bacteria	Hydrogenedentes	unclassified	unclassified	unclassified	unclassified	unclassified	-0.048	-0.232	-0.434	0.714	0	0	0	1	0.71	0.021
**Arctic-S**	OTUp_3905	Bacteria	Parcubacteria	uncultured_bacterium	unclassified	unclassified	unclassified	unclassified	-0.277	-0.277	-0.277	0.830	0	0	0	1	0.83	0.028
**Arctic-S**	OTUp_3915	Bacteria	Bacteroidetes	Flavobacteriia	Flavobacteriales	Flavobacteriaceae	Flavobacterium	uncultured_Flavobacterium_sp.	-0.374	-0.529	0.673	0.231	0	0	1	1	0.78	0.005
**Arctic-S**	OTUp_3917	Bacteria	Chloroflexi	Anaerolineae	Anaerolineales	Anaerolineaceae	uncultured	uncultured_sludge_bacterium_S14	-0.620	-0.407	0.505	0.522	0	0	1	1	0.89	0.000
**Arctic-S**	OTUp_3981	Bacteria	Cyanobacteria	ML635J-21	unclassified	unclassified	unclassified	unclassified	-0.330	-0.064	-0.330	0.724	0	0	0	1	0.72	0.030
**Arctic-S**	OTUp_4027	Bacteria	Saccharibacteria	uncultured_Candidatus_Saccharibacteria	unclassified	unclassified	unclassified	unclassified	-0.023	-0.343	-0.255	0.621	0	0	0	1	0.62	0.044
**Arctic-S**	OTUp_4068	Bacteria	Gemmatimonadetes	Gemmatimonadetes	Gemmatimonadales	Gemmatimonadaceae	Gemmatimonas	unclassified	-0.404	-0.404	0.554	0.255	0	0	1	1	0.70	0.023
**Arctic-S**	OTUp_4169	Bacteria	Proteobacteria	Betaproteobacteria	SC-I-84	uncultured_bacterium	unclassified	unclassified	-0.367	-0.367	0.758	-0.025	0	0	1	0	0.76	0.011
**Arctic-S**	OTUp_4272	Bacteria	Acidobacteria	Acidobacteria	Subgroup_2	bacterium_Ellin7505	unclassified	unclassified	-0.451	-0.254	-0.075	0.780	0	0	0	1	0.78	0.002
**Arctic-S**	OTUp_4293	Bacteria	Verrucomicrobia	Spartobacteria	Chthoniobacterales	DA101_soil_group	uncultured_bacterium	unclassified	-0.542	-0.296	0.224	0.614	0	0	1	1	0.73	0.005
**Arctic-S**	OTUp_4474	Bacteria	Proteobacteria	Deltaproteobacteria	Oligoflexales	uncultured_soil_bacterium	unclassified	unclassified	-0.260	-0.260	0.779	-0.260	0	0	1	0	0.78	0.030
**Arctic-S**	OTUp_4510	Bacteria	Gracilibacteria	uncultured_Microgenomates_bacterium	unclassified	unclassified	unclassified	unclassified	-0.277	-0.277	-0.277	0.831	0	0	0	1	0.83	0.030
**Arctic-S**	OTUp_4650	Bacteria	Planctomycetes	Planctomycetacia	Planctomycetales	Planctomycetaceae	Gemmata	uncultured_bacterium	-0.471	-0.270	0.347	0.394	0	0	1	1	0.64	0.049
**Arctic-S**	OTUp_4760	Bacteria	Bacteroidetes	Sphingobacteriia	Sphingobacteriales	Sphingobacteriaceae	Mucilaginibacter	uncultured_Mucilaginibacter_sp.	-0.206	-0.333	0.730	-0.190	0	0	1	0	0.73	0.030
**Arctic-S**	OTUp_4881	Bacteria	Planctomycetes	Planctomycetacia	Planctomycetales	Planctomycetaceae	uncultured	uncultured_bacterium	-0.149	-0.064	-0.433	0.646	0	0	0	1	0.65	0.049
**Arctic-S**	OTUp_4887	Bacteria	Verrucomicrobia	Spartobacteria	Chthoniobacterales	DA101_soil_group	uncultured_soil_bacterium	unclassified	-0.133	-0.286	-0.253	0.672	0	0	0	1	0.67	0.029
**Arctic-S**	OTUp_5001	Bacteria	Chloroflexi	JG30-KF-CM66	uncultured_Chloroflexi_bacterium	unclassified	unclassified	unclassified	-0.165	-0.622	0.453	0.334	0	0	1	1	0.68	0.028
**Arctic-S**	OTUp_5018	Bacteria	Proteobacteria	Alphaproteobacteria	Rhodospirillales	Rhodospirillaceae	Elstera	uncultured_alpha_proteobacterium	-0.058	-0.328	-0.247	0.633	0	0	0	1	0.63	0.041
**Arctic-S**	OTUp_5019	Bacteria	Verrucomicrobia	Spartobacteria	Chthoniobacterales	DA101_soil_group	uncultured_Verrucomicrobia_bacterium	unclassified	-0.478	-0.279	0.408	0.349	0	0	1	1	0.66	0.032
**Arctic-S**	OTUp_5066	Bacteria	Planctomycetes	Phycisphaerae	WD2101_soil_group	unclassified	unclassified	unclassified	-0.451	-0.444	0.391	0.504	0	0	1	1	0.78	0.005
**Arctic-S**	OTUp_5172	Bacteria	Acidobacteria	Acidobacteria	Subgroup_6	uncultured_Anaeromyxobacter_sp.	unclassified	unclassified	-0.383	-0.424	0.184	0.623	0	0	1	1	0.70	0.021
**Arctic-S**	OTUp_5208	Bacteria	Proteobacteria	Betaproteobacteria	Nitrosomonadales	Nitrosomonadaceae	uncultured	uncultured_delta_proteobacterium	-0.371	-0.472	0.063	0.780	0	0	0	1	0.78	0.008
**Arctic-S**	OTUp_5253	Bacteria	Proteobacteria	Betaproteobacteria	Rhodocyclales	Rhodocyclaceae	Georgfuchsia	uncultured_bacterium	-0.456	-0.304	0.226	0.534	0	0	1	1	0.66	0.027
**Arctic-S**	OTUp_5329	Bacteria	Bacteroidetes	Sphingobacteriia	Sphingobacteriales	Sphingobacteriaceae	Mucilaginibacter	Mucilaginibacter_sp._821-4-4-5	-0.367	-0.500	0.463	0.404	0	0	1	1	0.75	0.013
**Arctic-S**	OTUp_5351	Bacteria	WCHB1-60	uncultured_soil_bacterium	unclassified	unclassified	unclassified	unclassified	-0.301	-0.301	-0.101	0.703	0	0	0	1	0.70	0.028
**Arctic-S**	OTUp_5382	Bacteria	Bacteroidetes	Sphingobacteriia	Sphingobacteriales	Chitinophagaceae	uncultured	uncultured_bacterium	-0.297	-0.489	0.138	0.648	0	0	1	1	0.68	0.026
**Arctic-S**	OTUp_5458	Bacteria	Planctomycetes	Planctomycetacia	Planctomycetales	Planctomycetaceae	uncultured	uncultured_bacterium	-0.300	-0.077	-0.300	0.676	0	0	0	1	0.68	0.027
**Arctic-S**	OTUp_5498	Bacteria	Actinobacteria	Actinobacteria	Kineosporiales	Kineosporiaceae	Angustibacter	uncultured_bacterium	-0.236	-0.369	0.686	-0.081	0	0	1	0	0.69	0.015
**Arctic-S**	OTUp_5593	Bacteria	Acidobacteria	Acidobacteria	Subgroup_3	Unknown_Family	Candidatus_Solibacter	unclassified	-0.411	0.021	-0.255	0.646	0	0	0	1	0.65	0.047
**Arctic-S**	OTUp_5636	Bacteria	Acidobacteria	Acidobacteria	Subgroup_3	Unknown_Family	Bryobacter	uncultured_soil_bacterium	-0.550	-0.381	0.532	0.399	0	0	1	1	0.81	0.002
**Arctic-S**	OTUp_6162	Bacteria	Actinobacteria	MB-A2-108	unclassified	unclassified	unclassified	unclassified	-0.114	-0.277	-0.277	0.668	0	0	0	1	0.67	0.029
**Arctic-S**	OTUp_6182	Bacteria	Proteobacteria	Betaproteobacteria	Nitrosomonadales	Nitrosomonadaceae	uncultured	unclassified	-0.148	-0.285	-0.285	0.719	0	0	0	1	0.72	0.028
**Arctic-S**	OTUp_6198	Bacteria	Parcubacteria	uncultured_bacterium	unclassified	unclassified	unclassified	unclassified	-0.223	-0.228	-0.222	0.674	0	0	0	1	0.67	0.034
**Arctic-S**	OTUp_6277	Bacteria	Chloroflexi	unclassified	unclassified	unclassified	unclassified	unclassified	-0.033	-0.437	0.732	-0.262	0	0	1	0	0.73	0.020
**Arctic-S**	OTUp_6449	Bacteria	Proteobacteria	Alphaproteobacteria	Rhodospirillales	DA111	uncultured_alpha_proteobacterium	unclassified	-0.250	-0.480	0.321	0.409	0	0	1	1	0.63	0.049
**Arctic-S**	OTUp_6577	Bacteria	Bacteroidetes	Sphingobacteriia	Sphingobacteriales	Chitinophagaceae	Ferruginibacter	unclassified	-0.343	-0.445	0.332	0.456	0	0	1	1	0.68	0.031
**Arctic-S**	OTUp_6827	Bacteria	Acidobacteria	Acidobacteria	Subgroup_6	unclassified	unclassified	unclassified	-0.358	-0.358	0.863	-0.147	0	0	1	0	0.86	0.004
**Arctic-S**	OTUp_6903	Bacteria	Chloroflexi	Ktedonobacteria	Thermogemmatisporales	1921-2	uncultured_bacterium	unclassified	-0.462	0.318	0.474	-0.331	0	1	1	0	0.69	0.023
**Arctic-S**	OTUp_7092	Bacteria	Proteobacteria	Alphaproteobacteria	Rhodospirillales	Rhodospirillales_Incertae_Sedis	Reyranella	uncultured_soil_bacterium	-0.061	-0.390	-0.282	0.733	0	0	0	1	0.73	0.015
**Arctic-S**	OTUp_7186	Bacteria	Planctomycetes	Planctomycetacia	Planctomycetales	Planctomycetaceae	Gemmata	uncultured_planctomycete	-0.361	-0.350	-0.135	0.845	0	0	0	1	0.84	0.002
**Arctic-S**	OTUp_7196	Bacteria	Proteobacteria	Alphaproteobacteria	DB1-14	uncultured_bacterium	unclassified	unclassified	-0.301	-0.098	0.701	-0.301	0	0	1	0	0.70	0.028
**Arctic-S**	OTUp_7272	Bacteria	Chloroflexi	KD4-96	unclassified	unclassified	unclassified	unclassified	-0.420	-0.314	0.437	0.297	0	0	1	1	0.64	0.045
**Arctic-S**	OTUp_7348	Bacteria	Proteobacteria	Betaproteobacteria	Nitrosomonadales	Nitrosomonadaceae	uncultured	uncultured_Oxalobacteraceae_bacterium	-0.531	-0.191	0.042	0.680	0	0	0	1	0.68	0.029
**Arctic-S**	OTUp_7373	Bacteria	Proteobacteria	Deltaproteobacteria	Myxococcales	Polyangiaceae	Sorangium	uncultured_delta_proteobacterium	-0.216	-0.225	0.793	-0.352	0	0	1	0	0.79	0.003
**Arctic-S**	OTUp_7405	Bacteria	Planctomycetes	OM190	uncultured_bacterium	unclassified	unclassified	unclassified	-0.408	-0.408	0.077	0.739	0	0	0	1	0.74	0.008
**Arctic-S**	OTUp_7617	Bacteria	Planctomycetes	Planctomycetacia	Planctomycetales	Planctomycetaceae	uncultured	uncultured_bacterium	-0.537	-0.254	0.136	0.656	0	0	1	1	0.69	0.036
**Arctic-S**	OTUp_7675	Bacteria	Proteobacteria	Alphaproteobacteria	Caulobacterales	Hyphomonadaceae	Woodsholea	uncultured_proteobacterium	-0.254	-0.254	-0.254	0.763	0	0	0	1	0.76	0.030
**Arctic-S**	OTUp_7731	Bacteria	Elusimicrobia	Elusimicrobia	Lineage_IIa	uncultured_bacterium	unclassified	unclassified	-0.556	0.400	-0.340	0.496	0	1	0	1	0.78	0.006
**Arctic-S**	OTUp_7879	Bacteria	Acidobacteria	Holophagae	Subgroup_7	unclassified	unclassified	unclassified	-0.246	-0.389	-0.097	0.732	0	0	0	1	0.73	0.021
**Arctic-S**	OTUp_7891	Bacteria	Acidobacteria	Acidobacteria	Subgroup_4	RB41	uncultured_soil_bacterium	unclassified	-0.557	-0.407	0.462	0.502	0	0	1	1	0.83	0.003
**Arctic-S**	OTUp_7946	Bacteria	Verrucomicrobia	OPB35_soil_group	uncultured_bacterium	unclassified	unclassified	unclassified	-0.353	-0.164	-0.153	0.669	0	0	0	1	0.67	0.048
**Arctic-S**	OTUp_8190	Bacteria	Verrucomicrobia	Spartobacteria	Chthoniobacterales	DA101_soil_group	unclassified	unclassified	-0.555	-0.331	0.038	0.849	0	0	0	1	0.85	0.002
**Arctic-S**	OTUp_8256	Bacteria	Proteobacteria	Betaproteobacteria	Burkholderiales	Oxalobacteraceae	Undibacterium	uncultured_Oxalobacteraceae_bacterium	-0.247	-0.247	0.740	-0.247	0	0	1	0	0.74	0.029
**Arctic-S**	OTUp_8293	Bacteria	Acidobacteria	Acidobacteria	Subgroup_4	RB41	uncultured_bacterium	unclassified	-0.337	-0.381	-0.125	0.842	0	0	0	1	0.84	0.004
**Arctic-S**	OTUp_8527	Bacteria	Chloroflexi	Ktedonobacteria	JG30-KF-AS9	Chloroflexi_bacterium_Ellin7237	unclassified	unclassified	-0.255	-0.255	0.766	-0.255	0	0	1	0	0.77	0.030
**Arctic-S**	OTUp_8559	Bacteria	Chloroflexi	KD4-96	unclassified	unclassified	unclassified	unclassified	-0.359	-0.364	0.706	0.017	0	0	1	0	0.71	0.017
**Arctic-S**	OTUp_9069	Bacteria	Proteobacteria	Gammaproteobacteria	Xanthomonadales	Xanthomonadaceae	unclassified	unclassified	-0.635	-0.148	0.269	0.513	0	0	1	1	0.68	0.018
**Arctic-S**	OTUp_9151	Bacteria	Planctomycetes	Planctomycetacia	Planctomycetales	Planctomycetaceae	uncultured	uncultured_bacterium	-0.338	-0.338	-0.003	0.680	0	0	0	1	0.68	0.031
**Arctic-S**	OTUp_9193	Bacteria	Saccharibacteria	uncultured_soil_bacterium	unclassified	unclassified	unclassified	unclassified	-0.263	-0.263	-0.263	0.788	0	0	0	1	0.79	0.029
**Arctic-S**	OTUp_9226	Bacteria	Actinobacteria	Acidimicrobiia	Acidimicrobiales	uncultured	uncultured_bacterium	unclassified	-0.335	0.586	0.209	-0.460	0	1	1	0	0.69	0.047
**Arctic-S**	OTUp_9235	Bacteria	Actinobacteria	Actinobacteria	Frankiales	Acidothermaceae	Acidothermus	unclassified	-0.213	-0.219	0.803	-0.371	0	0	1	0	0.80	0.006
**Arctic-S**	OTUp_9517	Bacteria	Bacteroidetes	Sphingobacteriia	Sphingobacteriales	Chitinophagaceae	Terrimonas	uncultured_bacterium	-0.075	-0.403	-0.310	0.788	0	0	0	1	0.79	0.005
**Arctic-S**	OTUp_9577	Bacteria	Bacteroidetes	Sphingobacteriia	Sphingobacteriales	unclassified	unclassified	unclassified	-0.303	-0.006	-0.303	0.613	0	0	0	1	0.61	0.034
**Arctic-S**	OTUp_9692	Bacteria	Proteobacteria	Deltaproteobacteria	Myxococcales	Haliangiaceae	Haliangium	unclassified	-0.630	0.309	0.490	-0.169	0	1	1	0	0.69	0.019
**Arctic-S**	OTUp_9841	Bacteria	Acidobacteria	Holophagae	Subgroup_10	ABS-19	uncultured_bacterium	unclassified	-0.388	-0.388	0.215	0.561	0	0	1	1	0.67	0.025
**Arctic-S**	OTUp_10024	Bacteria	Verrucomicrobia	Spartobacteria	Chthoniobacterales	DA101_soil_group	unclassified	unclassified	-0.513	-0.469	0.423	0.559	0	0	1	1	0.85	0.000
**Arctic-S**	OTUp_10824	Bacteria	Acidobacteria	Acidobacteria	Subgroup_2	unclassified	unclassified	unclassified	-0.299	-0.365	-0.006	0.670	0	0	0	1	0.67	0.028
**Arctic-S**	OTUp_11232	Bacteria	Proteobacteria	Alphaproteobacteria	Rickettsiales	SM2D12	uncultured_bacterium	unclassified	-0.363	-0.177	0.677	-0.137	0	0	1	0	0.68	0.035
**Arctic-S**	OTUp_11251	Bacteria	Planctomycetes	Planctomycetacia	Planctomycetales	Planctomycetaceae	uncultured	uncultured_bacterium	-0.669	-0.026	0.355	0.340	0	1	1	1	0.67	0.037
**Alps-N**	OTUp_4	Bacteria	Actinobacteria	Actinobacteria	Propionibacteriales	Nocardioidaceae	Nocardioides	unclassified	0.117	-0.574	0.809	-0.353	0	0	1	0	0.81	0.002
**Alps-N**	OTUp_7	Bacteria	Proteobacteria	Alphaproteobacteria	Rhizobiales	Beijerinckiaceae	uncultured	uncultured_bacterium	-0.523	0.603	0.253	-0.333	0	1	1	0	0.74	0.010
**Alps-N**	OTUp_8	Bacteria	Chloroflexi	JG37-AG-4	uncultured_Clostridium_sp.	unclassified	unclassified	unclassified	-0.820	0.454	0.345	0.022	0	1	1	1	0.82	0.002
**Alps-N**	OTUp_9	Bacteria	Chloroflexi	JG37-AG-4	uncultured_bacterium	unclassified	unclassified	unclassified	-0.837	0.124	0.339	0.374	0	1	1	1	0.84	0.002
**Alps-N**	OTUp_11	Bacteria	Verrucomicrobia	Spartobacteria	Chthoniobacterales	DA101_soil_group	uncultured_soil_bacterium	unclassified	-0.571	-0.155	-0.062	0.788	0	0	0	1	0.79	0.002
**Alps-N**	OTUp_12	Bacteria	Verrucomicrobia	Spartobacteria	Chthoniobacterales	DA101_soil_group	uncultured_Verrucomicrobia_bacterium	unclassified	-0.529	-0.166	-0.147	0.842	0	0	0	1	0.84	0.002
**Alps-N**	OTUp_15	Bacteria	Proteobacteria	Betaproteobacteria	SC-I-84	uncultured_beta_proteobacterium	unclassified	unclassified	-0.878	0.211	0.245	0.422	0	1	1	1	0.88	0.002
**Alps-N**	OTUp_21	Bacteria	Planctomycetes	Planctomycetacia	Planctomycetales	Planctomycetaceae	uncultured	uncultured_Zavarzinella_sp.	-0.680	0.433	0.405	-0.158	0	1	1	0	0.73	0.008
**Alps-N**	OTUp_28	Bacteria	Proteobacteria	Gammaproteobacteria	Xanthomonadales	uncultured	uncultured_eubacterium_WD260	unclassified	-0.816	0.049	0.163	0.604	0	1	1	1	0.82	0.004
**Alps-N**	OTUp_31	Bacteria	Actinobacteria	Actinobacteria	Micrococcales	Micrococcaceae	Arthrobacter	unclassified	0.072	-0.567	0.763	-0.268	0	0	1	0	0.76	0.002
**Alps-N**	OTUp_43	Bacteria	Acidobacteria	Acidobacteria	Subgroup_3	Unknown_Family	Bryobacter	uncultured_Acidobacteria_bacterium	-0.646	0.457	0.105	0.083	0	1	1	1	0.65	0.041
**Alps-N**	OTUp_46	Bacteria	WD272	uncultured_Firmicutes_bacterium	unclassified	unclassified	unclassified	unclassified	-0.500	-0.218	-0.110	0.828	0	0	0	1	0.83	0.002

**Alps-N**	OTUp_47	Bacteria	Planctomycetes	Planctomycetacia	Planctomycetales	Planctomycetaceae	uncultured	unclassified	-0.637	0.624	0.280	-0.267	0	1	1	0	0.78	0.002
**Alps-N**	OTUp_50	Bacteria	WD272	unclassified	unclassified	unclassified	unclassified	unclassified	-0.639	-0.053	-0.110	0.802	0	0	0	1	0.80	0.002
**Alps-N**	OTUp_52	Bacteria	Actinobacteria	Actinobacteria	Pseudonocardiales	Pseudonocardiaceae	Crossiella	unclassified	-0.477	0.638	0.139	-0.301	0	1	1	0	0.67	0.029
**Alps-N**	OTUp_54	Bacteria	Acidobacteria	Acidobacteria	Subgroup_3	Unknown_Family	Bryobacter	uncultured_Holophaga_sp.	-0.416	0.560	0.213	-0.358	0	1	1	0	0.67	0.030
**Alps-N**	OTUp_56	Bacteria	Chloroflexi	S085	uncultured_bacterium	unclassified	unclassified	unclassified	-0.692	0.015	0.082	0.595	0	1	1	1	0.69	0.002
**Alps-N**	OTUp_59	Bacteria	Actinobacteria	Thermoleophilia	Gaiellales	Gaiellaceae	Gaiella	unclassified	-0.592	0.466	0.395	-0.270	0	1	1	0	0.75	0.009
**Alps-N**	OTUp_67	Bacteria	Actinobacteria	Acidimicrobiia	Acidimicrobiales	uncultured	uncultured_Ferrimicrobium_sp.	unclassified	-0.715	0.431	0.478	-0.194	0	1	1	0	0.79	0.005
**Alps-N**	OTUp_68	Bacteria	Chloroflexi	uncultured_Bellilinea_sp.	unclassified	unclassified	unclassified	unclassified	-0.536	0.660	0.174	-0.298	0	1	1	0	0.72	0.017
**Alps-N**	OTUp_73	Bacteria	WD272	uncultured_bacterium	unclassified	unclassified	unclassified	unclassified	-0.368	-0.004	-0.517	0.888	0	0	0	1	0.89	0.001
**Alps-N**	OTUp_75	Bacteria	Acidobacteria	Holophagae	Subgroup_7	uncultured_proteobacterium	unclassified	unclassified	-0.686	0.245	0.450	-0.009	0	1	1	1	0.69	0.015
**Alps-N**	OTUp_77	Bacteria	Verrucomicrobia	Spartobacteria	Chthoniobacterales	DA101_soil_group	uncultured_Verrucomicrobia_bacterium	unclassified	-0.606	-0.090	-0.085	0.781	0	0	0	1	0.78	0.005
**Alps-N**	OTUp_80	Bacteria	Acidobacteria	Acidobacteria	Subgroup_3	Unknown_Family	Bryobacter	uncultured_bacterium	-0.806	0.340	0.415	0.052	0	1	1	1	0.81	0.002
**Alps-N**	OTUp_82	Bacteria	WD272	uncultured_Thermoanaerobacteraceae	unclassified	unclassified	unclassified	unclassified	-0.446	-0.125	-0.221	0.791	0	0	0	1	0.79	0.002
**Alps-N**	OTUp_86	Bacteria	Chloroflexi	JG37-AG-4	uncultured_bacterium	unclassified	unclassified	unclassified	-0.616	0.194	0.581	-0.159	0	1	1	0	0.67	0.034
**Alps-N**	OTUp_88	Bacteria	Verrucomicrobia	Spartobacteria	Chthoniobacterales	DA101_soil_group	uncultured_Verrucomicrobia_bacterium	unclassified	-0.688	0.104	-0.140	0.724	0	0	0	1	0.72	0.012
**Alps-N**	OTUp_104	Bacteria	Verrucomicrobia	Spartobacteria	Chthoniobacterales	DA101_soil_group	uncultured_bacterium	unclassified	-0.533	-0.110	-0.075	0.718	0	0	0	1	0.72	0.002
**Alps-N**	OTUp_112	Bacteria	Proteobacteria	Alphaproteobacteria	Caulobacterales	Caulobacteraceae	uncultured	uncultured_bacterium	-0.578	-0.218	0.022	0.774	0	0	0	1	0.77	0.003
**Alps-N**	OTUp_113	Bacteria	Proteobacteria	Alphaproteobacteria	Sphingomonadales	Sphingomonadaceae	Sphingomonas	unclassified	-0.441	-0.533	0.349	0.624	0	0	1	1	0.84	0.000
**Alps-N**	OTUp_114	Bacteria	Proteobacteria	Betaproteobacteria	Burkholderiales	unclassified	unclassified	unclassified	-0.758	-0.034	0.201	0.592	0	1	1	1	0.76	0.004
**Alps-N**	OTUp_124	Bacteria	Verrucomicrobia	Spartobacteria	Chthoniobacterales	DA101_soil_group	uncultured_bacterium	unclassified	-0.357	-0.297	-0.148	0.801	0	0	0	1	0.80	0.002
**Alps-N**	OTUp_125	Bacteria	Planctomycetes	Planctomycetacia	Planctomycetales	Planctomycetaceae	unclassified	unclassified	-0.435	0.198	-0.427	0.664	0	1	0	1	0.75	0.005
**Alps-N**	OTUp_127	Bacteria	Acidobacteria	Holophagae	Subgroup_7	uncultured_proteobacterium	unclassified	unclassified	-0.892	0.291	0.187	0.414	0	1	1	1	0.89	0.002
**Alps-N**	OTUp_132	Bacteria	Proteobacteria	Gammaproteobacteria	Xanthomonadales	Xanthomonadaceae	Silanimonas	uncultured_Xanthomonadaceae_bacterium	-0.608	-0.121	0.235	0.495	0	0	1	1	0.63	0.044
**Alps-N**	OTUp_138	Bacteria	Proteobacteria	Gammaproteobacteria	Xanthomonadales	Xanthomonadaceae	unclassified	unclassified	-0.779	0.045	0.062	0.672	0	1	1	1	0.78	0.002
**Alps-N**	OTUp_139	Bacteria	Proteobacteria	Betaproteobacteria	Burkholderiales	Oxalobacteraceae	uncultured	uncultured_bacterium	0.040	-0.458	0.595	-0.177	0	0	1	0	0.60	0.032
**Alps-N**	OTUp_141	Bacteria	Actinobacteria	Acidimicrobiia	Acidimicrobiales	Acidimicrobiaceae	uncultured	uncultured_bacterium	-0.580	0.328	-0.227	0.479	0	1	0	1	0.70	0.024
**Alps-N**	OTUp_147	Bacteria	Proteobacteria	Alphaproteobacteria	Rhizobiales	Rhodobiaceae	uncultured	uncultured_Rhizobiales_bacterium	-0.458	0.256	0.583	-0.381	0	1	1	0	0.73	0.008
**Alps-N**	OTUp_149	Bacteria	Acidobacteria	Acidobacteria	Subgroup_3	Unknown_Family	Candidatus_Solibacter	uncultured_Acidobacteria_bacterium	-0.629	0.311	0.223	0.095	0	1	1	1	0.63	0.047
**Alps-N**	OTUp_158	Bacteria	Proteobacteria	Betaproteobacteria	Nitrosomonadales	Nitrosomonadaceae	uncultured	unclassified	-0.817	0.254	0.325	0.238	0	1	1	1	0.82	0.003
**Alps-N**	OTUp_163	Bacteria	Verrucomicrobia	S-BQ2-57_soil_group	uncultured_bacterium	unclassified	unclassified	unclassified	-0.177	-0.298	0.660	-0.185	0	0	1	0	0.66	0.024
**Alps-N**	OTUp_165	Bacteria	Actinobacteria	Acidimicrobiia	Acidimicrobiales	uncultured	uncultured_actinobacterium	unclassified	-0.652	0.202	0.333	0.117	0	1	1	1	0.65	0.046
**Alps-N**	OTUp_168	Bacteria	Acidobacteria	Holophagae	Subgroup_7	unclassified	unclassified	unclassified	-0.497	-0.096	-0.274	0.867	0	0	0	1	0.87	0.002
**Alps-N**	OTUp_171	Bacteria	Planctomycetes	Planctomycetacia	Planctomycetales	Planctomycetaceae	Gemmata	uncultured_soil_bacterium	-0.668	0.360	0.435	-0.128	0	1	1	0	0.69	0.022
**Alps-N**	OTUp_172	Bacteria	Proteobacteria	Alphaproteobacteria	Sphingomonadales	Sphingomonadaceae	Sphingomonas	Sphingomonas_faeni	-0.456	-0.181	0.641	-0.003	0	0	1	0	0.64	0.040
**Alps-N**	OTUp_174	Bacteria	Actinobacteria	Actinobacteria	Frankiales	uncultured	uncultured_bacterium	unclassified	-0.228	-0.443	0.891	-0.220	0	0	1	0	0.89	0.002
**Alps-N**	OTUp_176	Bacteria	Actinobacteria	Thermoleophilia	Gaiellales	uncultured	unclassified	unclassified	-0.591	0.226	0.559	-0.193	0	1	1	0	0.68	0.027
**Alps-N**	OTUp_183	Bacteria	Proteobacteria	Alphaproteobacteria	Rhizobiales	Rhizobiales_Incertae_Sedis	Bauldia	uncultured_Phyllobacteriaceae_bacterium	-0.818	0.444	0.222	0.152	0	1	1	1	0.82	0.005
**Alps-N**	OTUp_187	Bacteria	Gemmatimonadetes	Gemmatimonadetes	Gemmatimonadales	Gemmatimonadaceae	uncultured	uncultured_Gemmatimonadales_bacterium	-0.707	0.446	0.336	-0.076	0	1	1	1	0.71	0.014
**Alps-N**	OTUp_188	Bacteria	Verrucomicrobia	Spartobacteria	Chthoniobacterales	DA101_soil_group	uncultured_Verrucomicrobia_bacterium	unclassified	-0.546	-0.059	-0.255	0.860	0	0	0	1	0.86	0.002
**Alps-N**	OTUp_195	Bacteria	Chloroflexi	KD4-96	uncultured_Chloroflexi_bacterium	unclassified	unclassified	unclassified	-0.572	0.302	0.433	-0.163	0	1	1	0	0.64	0.045
**Alps-N**	OTUp_196	Bacteria	Bacteroidetes	Sphingobacteriia	Sphingobacteriales	Chitinophagaceae	uncultured	uncultured_Chitinophagaceae_bacterium	-0.488	-0.138	0.004	0.622	0	0	0	1	0.62	0.050
**Alps-N**	OTUp_203	Bacteria	Actinobacteria	Acidimicrobiia	Acidimicrobiales	uncultured	uncultured_Acidimicrobiaceae_bacterium	unclassified	-0.443	0.190	-0.308	0.561	0	1	0	1	0.65	0.039
**Alps-N**	OTUp_210	Bacteria	Acidobacteria	Acidobacteria	Subgroup_6	unclassified	unclassified	unclassified	-0.705	0.338	0.137	0.230	0	1	1	1	0.71	0.015
**Alps-N**	OTUp_213	Bacteria	Nitrospirae	Nitrospira	Nitrospirales	0319-6A21	uncultured_candidate_division_SPAM_bacterium	unclassified	-0.523	0.444	-0.219	0.298	0	1	0	1	0.64	0.047
**Alps-N**	OTUp_214	Bacteria	Actinobacteria	Thermoleophilia	Solirubrobacterales	TM146	uncultured_Conexibacteraceae_bacterium	unclassified	-0.657	0.406	0.286	-0.034	0	1	1	1	0.66	0.040
**Alps-N**	OTUp_216	Bacteria	Proteobacteria	Alphaproteobacteria	Rhizobiales	Xanthobacteraceae	Variibacter	uncultured_Hyphomicrobiaceae_bacterium	-0.669	0.515	0.153	0.001	0	1	1	1	0.67	0.029
**Alps-N**	OTUp_220	Bacteria	Acidobacteria	Acidobacteria	Subgroup_3	Unknown_Family	Candidatus_Solibacter	unclassified	-0.848	0.531	0.185	0.132	0	1	1	1	0.85	0.001
**Alps-N**	OTUp_227	Bacteria	WD272	uncultured_bacterium	unclassified	unclassified	unclassified	unclassified	-0.197	-0.362	-0.111	0.670	0	0	0	1	0.67	0.024
**Alps-N**	OTUp_228	Bacteria	Proteobacteria	Alphaproteobacteria	Sphingomonadales	Sphingomonadaceae	Sphingomonas	unclassified	-0.343	-0.481	0.440	0.384	0	0	1	1	0.71	0.018
**Alps-N**	OTUp_229	Bacteria	Planctomycetes	Planctomycetacia	Planctomycetales	Planctomycetaceae	uncultured	uncultured_planctomycete	-0.641	0.632	-0.243	0.252	0	1	0	1	0.77	0.004
**Alps-N**	OTUp_244	Bacteria	Planctomycetes	Planctomycetacia	Planctomycetales	Planctomycetaceae	Gemmata	uncultured_bacterium	-0.779	0.228	0.431	0.120	0	1	1	1	0.78	0.007
**Alps-N**	OTUp_251	Bacteria	WD272	uncultured_bacterium	unclassified	unclassified	unclassified	unclassified	-0.438	-0.210	-0.239	0.887	0	0	0	1	0.89	0.001
**Alps-N**	OTUp_262	Bacteria	Planctomycetes	Planctomycetacia	Planctomycetales	Planctomycetaceae	Zavarzinella	unclassified	-0.751	0.207	0.197	0.347	0	1	1	1	0.75	0.008
**Alps-N**	OTUp_266	Bacteria	Actinobacteria	Acidimicrobiia	Acidimicrobiales	uncultured	uncultured_bacterium	unclassified	-0.674	0.221	0.309	0.144	0	1	1	1	0.67	0.031
**Alps-N**	OTUp_283	Bacteria	Proteobacteria	Gammaproteobacteria	Xanthomonadales	Xanthomonadaceae	Rhodanobacter	uncultured_Xanthomonadaceae_bacterium	-0.661	0.125	0.126	0.410	0	1	1	1	0.66	0.036
**Alps-N**	OTUp_296	Bacteria	Gemmatimonadetes	Gemmatimonadetes	Gemmatimonadales	Gemmatimonadaceae	Gemmatimonas	unclassified	-0.623	0.468	0.328	-0.172	0	1	1	0	0.69	0.020
**Alps-N**	OTUp_311	Bacteria	Proteobacteria	Alphaproteobacteria	Rhizobiales	Hyphomicrobiaceae	Pedomicrobium	uncultured_bacterium	-0.358	-0.234	0.834	-0.241	0	0	1	0	0.83	0.002
**Alps-N**	OTUp_318	Bacteria	Planctomycetes	Planctomycetacia	Planctomycetales	Planctomycetaceae	uncultured	unclassified	-0.798	0.489	0.160	0.150	0	1	1	1	0.80	0.005
**Alps-N**	OTUp_364	Bacteria	Verrucomicrobia	Opitutae	Opitutales	Opitutaceae	Opitutus	uncultured_bacterium	-0.257	-0.257	-0.257	0.770	0	0	0	1	0.77	0.028
**Alps-N**	OTUp_373	Bacteria	Actinobacteria	Acidimicrobiia	Acidimicrobiales	uncultured	bacterium_enrichment_culture_clone_auto73_4W	unclassified	-0.687	0.310	0.296	0.081	0	1	1	1	0.69	0.026
**Alps-N**	OTUp_379	Bacteria	Acidobacteria	Acidobacteria	Subgroup_4	Unknown_Family	Blastocatella	uncultured_Acidobacteria_bacterium	0.024	-0.157	-0.484	0.617	0	0	0	1	0.62	0.043
**Alps-N**	OTUp_384	Bacteria	WD272	uncultured_Firmicutes_bacterium	unclassified	unclassified	unclassified	unclassified	-0.597	-0.061	-0.115	0.772	0	0	0	1	0.77	0.008
**Alps-N**	OTUp_385	Bacteria	Verrucomicrobia	Spartobacteria	Chthoniobacterales	DA101_soil_group	uncultured_Verrucomicrobia_bacterium	unclassified	-0.724	0.120	0.058	0.546	0	1	1	1	0.72	0.007
**Alps-N**	OTUp_387	Bacteria	Proteobacteria	Betaproteobacteria	TRA3-20	unclassified	unclassified	unclassified	-0.773	0.406	0.365	0.002	0	1	1	1	0.77	0.007
**Alps-N**	OTUp_389	Bacteria	Chloroflexi	Gitt-GS-136	uncultured_Caldilineaceae_bacterium	unclassified	unclassified	unclassified	-0.694	0.076	0.376	0.242	0	1	1	1	0.69	0.020
**Alps-N**	OTUp_396	Bacteria	Proteobacteria	Alphaproteobacteria	Sphingomonadales	Sphingomonadaceae	unclassified	unclassified	-0.420	-0.381	0.200	0.601	0	0	1	1	0.69	0.013
**Alps-N**	OTUp_398	Bacteria	Verrucomicrobia	Spartobacteria	Chthoniobacterales	Chthoniobacteraceae	Chthoniobacter	uncultured_bacterium	-0.277	-0.277	0.830	-0.277	0	0	1	0	0.83	0.027
**Alps-N**	OTUp_400	Bacteria	Planctomycetes	Planctomycetacia	Planctomycetales	Planctomycetaceae	uncultured	uncultured_Planctomycetia_bacterium	-0.719	0.233	0.248	0.238	0	1	1	1	0.72	0.012
**Alps-N**	OTUp_403	Bacteria	Actinobacteria	Thermoleophilia	Gaiellales	Gaiellaceae	Gaiella	uncultured_bacterium	-0.599	0.254	0.602	-0.256	0	1	1	0	0.74	0.003
**Alps-N**	OTUp_424	Bacteria	Proteobacteria	Betaproteobacteria	Burkholderiales	unclassified	unclassified	unclassified	-0.794	0.241	0.251	0.302	0	1	1	1	0.79	0.010
**Alps-N**	OTUp_428	Bacteria	Chloroflexi	Ktedonobacteria	Ktedonobacterales	JG30a-KF-32	uncultured_Ktedobacteria_bacterium	unclassified	-0.399	0.453	-0.487	0.434	0	1	0	1	0.77	0.006
**Alps-N**	OTUp_437	Bacteria	Planctomycetes	Planctomycetacia	Planctomycetales	Planctomycetaceae	Planctomyces	uncultured_bacterium	-0.657	0.567	-0.081	0.172	0	1	1	1	0.66	0.042
**Alps-N**	OTUp_441	Bacteria	Chloroflexi	KD4-96	uncultured_Chloroflexi_bacterium	unclassified	unclassified	unclassified	-0.674	0.151	0.271	0.253	0	1	1	1	0.67	0.026
**Alps-N**	OTUp_447	Bacteria	Planctomycetes	Planctomycetacia	Planctomycetales	Planctomycetaceae	Gemmata	unclassified	-0.505	0.532	0.242	-0.268	0	1	1	0	0.67	0.034
**Alps-N**	OTUp_454	Bacteria	Planctomycetes	Planctomycetacia	Planctomycetales	Planctomycetaceae	unclassified	unclassified	-0.322	0.067	-0.471	0.727	0	0	0	1	0.73	0.016
**Alps-N**	OTUp_466	Bacteria	Proteobacteria	Alphaproteobacteria	Rhodospirillales	Acetobacteraceae	uncultured	uncultured_soil_bacterium	-0.260	-0.096	0.727	-0.371	0	0	1	0	0.73	0.012
**Alps-N**	OTUp_538	Bacteria	Actinobacteria	Acidimicrobiia	Acidimicrobiales	uncultured	uncultured_bacterium	unclassified	-0.490	0.386	0.500	-0.396	0	1	1	0	0.77	0.007
**Alps-N**	OTUp_560	Bacteria	Actinobacteria	Thermoleophilia	Solirubrobacterales	unclassified	unclassified	unclassified	-0.343	0.479	0.263	-0.400	0	1	1	0	0.64	0.039
**Alps-N**	OTUp_561	Bacteria	Acidobacteria	Acidobacteria	Subgroup_3	Unknown_Family	Candidatus_Solibacter	uncultured_soil_bacterium	-0.554	-0.279	0.421	0.412	0	0	1	1	0.72	0.014
**Alps-N**	OTUp_570	Bacteria	WD272	unclassified	unclassified	unclassified	unclassified	unclassified	-0.523	0.222	-0.297	0.598	0	1	0	1	0.71	0.012
**Alps-N**	OTUp_582	Bacteria	Acidobacteria	Acidobacteria	Subgroup_6	uncultured_bacterium_270	unclassified	unclassified	-0.638	0.344	0.192	0.101	0	1	1	1	0.64	0.047
**Alps-N**	OTUp_594	Bacteria	Planctomycetes	Planctomycetacia	Planctomycetales	Planctomycetaceae	uncultured	unclassified	-0.413	-0.039	-0.209	0.660	0	0	0	1	0.66	0.029
**Alps-N**	OTUp_605	Bacteria	unclassified	unclassified	unclassified	unclassified	unclassified	unclassified	-0.414	-0.309	0.060	0.663	0	0	0	1	0.66	0.039
**Alps-N**	OTUp_612	Bacteria	Proteobacteria	Betaproteobacteria	Burkholderiales	Comamonadaceae	unclassified	unclassified	-0.453	0.166	0.603	-0.316	0	1	1	0	0.67	0.040
**Alps-N**	OTUp_619	Bacteria	Acidobacteria	Acidobacteria	Subgroup_6	unclassified	unclassified	unclassified	-0.562	0.514	0.216	-0.168	0	1	1	0	0.63	0.044
**Alps-N**	OTUp_635	Bacteria	Chloroflexi	JG30-KF-CM66	uncultured_bacterium	unclassified	unclassified	unclassified	-0.277	0.474	0.293	-0.490	0	1	1	0	0.66	0.028
**Alps-N**	OTUp_648	Bacteria	Planctomycetes	Planctomycetacia	Planctomycetales	Planctomycetaceae	uncultured	unclassified	-0.349	0.253	0.500	-0.404	0	1	1	0	0.65	0.041
**Alps-N**	OTUp_659	Bacteria	Proteobacteria	Deltaproteobacteria	Desulfuromonadales	unclassified	unclassified	unclassified	-0.343	0.095	0.592	-0.343	0	1	1	0	0.59	0.045
**Alps-N**	OTUp_666	Bacteria	Chloroflexi	JG30-KF-CM66	uncultured_bacterium	unclassified	unclassified	unclassified	-0.689	0.656	-0.051	0.084	0	1	1	1	0.69	0.017
**Alps-N**	OTUp_675	Bacteria	Planctomycetes	Planctomycetacia	Planctomycetales	Planctomycetaceae	uncultured	uncultured_bacterium	-0.675	0.399	0.010	0.266	0	1	1	1	0.67	0.023
**Alps-N**	OTUp_677	Bacteria	Verrucomicrobia	Spartobacteria	Chthoniobacterales	DA101_soil_group	uncultured_Verrucomicrobia_bacterium	unclassified	-0.429	-0.043	-0.299	0.771	0	0	0	1	0.77	0.009
**Alps-N**	OTUp_682	Bacteria	Proteobacteria	Alphaproteobacteria	Sphingomonadales	Sphingomonadaceae	Sphingomonas	unclassified	-0.402	-0.484	0.064	0.822	0	0	0	1	0.82	0.002
**Alps-N**	OTUp_683	Bacteria	Actinobacteria	Acidimicrobiia	Acidimicrobiales	uncultured	uncultured_Aciditerrimonas_sp.	unclassified	-0.362	0.320	0.518	-0.477	0	1	1	0	0.73	0.013
**Alps-N**	OTUp_706	Bacteria	Proteobacteria	Gammaproteobacteria	Xanthomonadales	Xanthomonadaceae	Lysobacter	unclassified	-0.532	-0.224	0.333	0.423	0	0	1	1	0.65	0.045
**Alps-N**	OTUp_708	Bacteria	Armatimonadetes	uncultured_bacterium	unclassified	unclassified	unclassified	unclassified	-0.494	-0.255	0.144	0.605	0	0	1	1	0.65	0.036
**Alps-N**	OTUp_716	Bacteria	WD272	uncultured_bacterium	unclassified	unclassified	unclassified	unclassified	-0.283	-0.322	-0.119	0.723	0	0	0	1	0.72	0.019

**Alps-N**	OTUp_720	Bacteria	Verrucomicrobia	Spartobacteria	Chthoniobacterales	DA101_soil_group	uncultured_Verrucomicrobia_bacterium	unclassified	-0.502	-0.200	0.010	0.691	0	0	0	1	0.69	0.012
**Alps-N**	OTUp_730	Bacteria	WD272	uncultured_cyanobacterium	unclassified	unclassified	unclassified	unclassified	-0.193	-0.124	-0.419	0.736	0	0	0	1	0.74	0.010
**Alps-N**	OTUp_742	Bacteria	WD272	uncultured_Firmicutes_bacterium	unclassified	unclassified	unclassified	unclassified	-0.541	-0.295	0.345	0.491	0	0	1	1	0.72	0.016
**Alps-N**	OTUp_751	Bacteria	Chloroflexi	JG37-AG-4	uncultured_bacterium	unclassified	unclassified	unclassified	-0.852	0.343	0.221	0.289	0	1	1	1	0.85	0.001
**Alps-N**	OTUp_756	Bacteria	Proteobacteria	Betaproteobacteria	Burkholderiales	Comamonadaceae	unclassified	unclassified	-0.534	-0.218	0.167	0.585	0	0	1	1	0.65	0.036
**Alps-N**	OTUp_760	Bacteria	Chloroflexi	KD4-96	uncultured_bacterium	unclassified	unclassified	unclassified	-0.254	0.002	0.649	-0.397	0	0	1	0	0.65	0.041
**Alps-N**	OTUp_773	Bacteria	Proteobacteria	Gammaproteobacteria	Xanthomonadales	Xanthomonadaceae	uncultured	unclassified	-0.460	0.163	-0.375	0.672	0	1	0	1	0.72	0.012
**Alps-N**	OTUp_784	Bacteria	Proteobacteria	Deltaproteobacteria	Myxococcales	Cystobacteraceae	Anaeromyxobacter	uncultured_bacterium	-0.404	0.570	-0.404	0.238	0	1	0	1	0.70	0.031
**Alps-N**	OTUp_787	Bacteria	Proteobacteria	Betaproteobacteria	SC-I-84	uncultured_beta_proteobacterium	unclassified	unclassified	-0.856	0.337	0.257	0.262	0	1	1	1	0.86	0.002
**Alps-N**	OTUp_797	Bacteria	Acidobacteria	Acidobacteria	Subgroup_3	Unknown_Family	Candidatus_Solibacter	uncultured_Acidobacteria_bacterium	-0.698	0.217	0.057	0.424	0	1	1	1	0.70	0.016
**Alps-N**	OTUp_804	Bacteria	Chloroflexi	JG37-AG-4	uncultured_bacterium	unclassified	unclassified	unclassified	-0.274	0.648	-0.480	0.105	0	1	0	1	0.65	0.039
**Alps-N**	OTUp_814	Bacteria	Gemmatimonadetes	Gemmatimonadetes	Gemmatimonadales	Gemmatimonadaceae	Gemmatimonas	uncultured_Gemmatimonas_sp.	-0.712	0.192	0.404	0.116	0	1	1	1	0.71	0.020
**Alps-N**	OTUp_817	Bacteria	Proteobacteria	Betaproteobacteria	SC-I-84	unclassified	unclassified	unclassified	-0.595	-0.181	0.057	0.719	0	0	0	1	0.72	0.015
**Alps-N**	OTUp_831	Bacteria	Proteobacteria	Deltaproteobacteria	Myxococcales	Polyangiaceae	Sorangium	uncultured_bacterium	-0.690	0.363	0.349	-0.022	0	1	1	1	0.69	0.021
**Alps-N**	OTUp_836	Bacteria	WD272	uncultured_Firmicutes_bacterium	unclassified	unclassified	unclassified	unclassified	-0.189	-0.244	-0.348	0.781	0	0	0	1	0.78	0.004
**Alps-N**	OTUp_842	Bacteria	Verrucomicrobia	Spartobacteria	Chthoniobacterales	DA101_soil_group	uncultured_Xiphinematobacteriaceae_bacterium	unclassified	-0.618	-0.230	0.060	0.789	0	0	0	1	0.79	0.005
**Alps-N**	OTUp_888	Bacteria	Actinobacteria	Acidimicrobiia	Acidimicrobiales	uncultured	bacterium_enrichment_culture_clone_auto73_4W	unclassified	-0.841	0.095	0.336	0.410	0	1	1	1	0.84	0.002
**Alps-N**	OTUp_908	Bacteria	Verrucomicrobia	Spartobacteria	Chthoniobacterales	DA101_soil_group	unclassified	unclassified	-0.636	0.005	0.386	0.245	0	1	1	1	0.64	0.047
**Alps-N**	OTUp_988	Bacteria	Proteobacteria	Deltaproteobacteria	Myxococcales	Polyangiaceae	Sorangium	unclassified	-0.458	-0.060	0.747	-0.230	0	0	1	0	0.75	0.006
**Alps-N**	OTUp_993	Bacteria	Proteobacteria	Alphaproteobacteria	Rhodospirillales	Acetobacteraceae	Acidiphilium	unclassified	-0.643	0.112	0.537	-0.006	0	1	1	1	0.64	0.042
**Alps-N**	OTUp_1005	Bacteria	Acidobacteria	Acidobacteria	Subgroup_6	unclassified	unclassified	unclassified	-0.316	-0.098	-0.375	0.789	0	0	0	1	0.79	0.006
**Alps-N**	OTUp_1036	Bacteria	Actinobacteria	Acidimicrobiia	Acidimicrobiales	uncultured	uncultured_actinobacterium	unclassified	-0.349	0.051	0.693	-0.396	0	0	1	0	0.69	0.021
**Alps-N**	OTUp_1039	Bacteria	Proteobacteria	Betaproteobacteria	Burkholderiales	Oxalobacteraceae	Noviherbaspirillum	unclassified	0.057	-0.626	0.835	-0.266	0	0	1	0	0.83	0.002
**Alps-N**	OTUp_1062	Bacteria	Acidobacteria	Holophagae	Subgroup_7	uncultured_bacterium	unclassified	unclassified	-0.637	0.062	0.401	0.174	0	1	1	1	0.64	0.048
**Alps-N**	OTUp_1085	Bacteria	Actinobacteria	Acidimicrobiia	Acidimicrobiales	uncultured	uncultured_actinobacterium	unclassified	-0.649	0.073	0.148	0.429	0	1	1	1	0.65	0.035
**Alps-N**	OTUp_1110	Bacteria	Armatimonadetes	uncultured_Armatimonadetes_bacterium	unclassified	unclassified	unclassified	unclassified	-0.526	-0.161	-0.063	0.750	0	0	0	1	0.75	0.009
**Alps-N**	OTUp_1203	Bacteria	Proteobacteria	Betaproteobacteria	Nitrosomonadales	Nitrosomonadaceae	uncultured	uncultured_Burkholderiaceae_bacterium	-0.489	-0.237	0.266	0.460	0	0	1	1	0.63	0.045
**Alps-N**	OTUp_1282	Bacteria	Planctomycetes	Planctomycetacia	Planctomycetales	Planctomycetaceae	uncultured	uncultured_bacterium	-0.481	0.379	-0.330	0.432	0	1	0	1	0.70	0.022
**Alps-N**	OTUp_1319	Bacteria	Planctomycetes	Planctomycetacia	Planctomycetales	Planctomycetaceae	uncultured	uncultured_planctomycete	-0.401	0.597	0.264	-0.460	0	1	1	0	0.75	0.008
**Alps-N**	OTUp_1355	Bacteria	Actinobacteria	Actinobacteria	Frankiales	unclassified	unclassified	unclassified	-0.371	-0.236	0.627	-0.020	0	0	1	0	0.63	0.042
**Alps-N**	OTUp_1361	Bacteria	Planctomycetes	Planctomycetacia	Planctomycetales	Planctomycetaceae	uncultured	uncultured_bacterium	-0.370	0.459	0.331	-0.420	0	1	1	0	0.68	0.027
**Alps-N**	OTUp_1379	Bacteria	Planctomycetes	Planctomycetacia	Planctomycetales	Planctomycetaceae	uncultured	unclassified	-0.559	-0.279	-0.004	0.842	0	0	0	1	0.84	0.002
**Alps-N**	OTUp_1400	Bacteria	Proteobacteria	Betaproteobacteria	TRA3-20	uncultured_beta_proteobacterium	unclassified	unclassified	-0.546	0.327	0.450	-0.232	0	1	1	0	0.67	0.033
**Alps-N**	OTUp_1424	Bacteria	Acidobacteria	Holophagae	Subgroup_7	uncultured_proteobacterium	unclassified	unclassified	-0.723	0.311	0.475	-0.063	0	1	1	1	0.72	0.010
**Alps-N**	OTUp_1471	Bacteria	Acidobacteria	Acidobacteria	Subgroup_6	unclassified	unclassified	unclassified	-0.681	0.438	0.232	0.011	0	1	1	1	0.68	0.024
**Alps-N**	OTUp_1550	Bacteria	Proteobacteria	Betaproteobacteria	SC-I-84	unclassified	unclassified	unclassified	-0.639	0.125	0.340	0.174	0	1	1	1	0.64	0.050
**Alps-N**	OTUp_1569	Bacteria	Proteobacteria	Betaproteobacteria	Burkholderiales	Comamonadaceae	unclassified	unclassified	-0.530	-0.310	0.645	0.195	0	0	1	1	0.73	0.012
**Alps-N**	OTUp_1604	Bacteria	Acidobacteria	Acidobacteria	Subgroup_6	uncultured_Acidobacteria_bacterium	unclassified	unclassified	-0.658	0.093	0.028	0.537	0	1	1	1	0.66	0.036
**Alps-N**	OTUp_1661	Bacteria	Actinobacteria	Thermoleophilia	Gaiellales	uncultured	unclassified	unclassified	-0.654	0.213	0.334	0.107	0	1	1	1	0.65	0.047
**Alps-N**	OTUp_1668	Bacteria	Proteobacteria	Gammaproteobacteria	Xanthomonadales	Xanthomonadaceae	unclassified	unclassified	-0.518	-0.271	0.523	0.266	0	0	1	1	0.68	0.019
**Alps-N**	OTUp_1733	Bacteria	Chloroflexi	Chloroflexia	Kallotenuales	AKIW781	uncultured_soil_bacterium	unclassified	-0.290	0.407	0.393	-0.509	0	1	1	0	0.69	0.024
**Alps-N**	OTUp_1859	Bacteria	Acidobacteria	Acidobacteria	Acidobacteriales	Acidobacteriaceae	Granulicella	Granulicella_arctica	-0.277	-0.277	0.831	-0.277	0	0	1	0	0.83	0.028
**Alps-N**	OTUp_1925	Bacteria	Chloroflexi	Ktedonobacteria	B12-WMSP1	uncultured_Chloroflexi_bacterium	unclassified	unclassified	-0.586	0.512	0.365	-0.290	0	1	1	0	0.76	0.004
**Alps-N**	OTUp_1938	Bacteria	Gemmatimonadetes	Gemmatimonadetes	Gemmatimonadales	Gemmatimonadaceae	uncultured	uncultured_prokaryote	-0.455	0.290	-0.312	0.476	0	1	0	1	0.66	0.047
**Alps-N**	OTUp_1939	Bacteria	unclassified	unclassified	unclassified	unclassified	unclassified	unclassified	-0.525	0.625	0.182	-0.282	0	1	1	0	0.70	0.018
**Alps-N**	OTUp_1997	Bacteria	Chloroflexi	Thermomicrobia	JG30-KF-CM45	uncultured_soil_bacterium	unclassified	unclassified	-0.339	-0.169	0.688	-0.180	0	0	1	0	0.69	0.026
**Alps-N**	OTUp_2109	Bacteria	Proteobacteria	Gammaproteobacteria	Legionellales	Coxiellaceae	Coxiella	uncultured_bacterium	-0.483	0.435	0.480	-0.432	0	1	1	0	0.79	0.007
**Alps-N**	OTUp_2128	Bacteria	Parcubacteria	uncultured_soil_bacterium	unclassified	unclassified	unclassified	unclassified	-0.269	0.457	-0.484	0.296	0	1	0	1	0.65	0.037
**Alps-N**	OTUp_2174	Bacteria	Bacteroidetes	Flavobacteriia	Flavobacteriales	Flavobacteriaceae	Flavobacterium	uncultured_Flavobacteriaceae_bacterium	-0.420	-0.062	0.660	-0.178	0	0	1	0	0.66	0.037
**Alps-N**	OTUp_2218	Bacteria	WD272	uncultured_bacterium	unclassified	unclassified	unclassified	unclassified	-0.472	-0.063	-0.210	0.744	0	0	0	1	0.74	0.002
**Alps-N**	OTUp_2228	Bacteria	unclassified	unclassified	unclassified	unclassified	unclassified	unclassified	-0.502	0.215	-0.339	0.626	0	1	0	1	0.73	0.006
**Alps-N**	OTUp_2324	Bacteria	Deinococcus-Thermus	Deinococci	Deinococcales	Deinococcaceae	Deinococcus	unclassified	-0.627	-0.096	0.654	0.069	0	0	1	0	0.65	0.035
**Alps-N**	OTUp_2464	Bacteria	Actinobacteria	Acidimicrobiia	Acidimicrobiales	uncultured_bacterium	unclassified	unclassified	-0.236	0.610	0.215	-0.589	0	1	1	0	0.71	0.016
**Alps-N**	OTUp_2619	Bacteria	Actinobacteria	Thermoleophilia	Gaiellales	uncultured	unclassified	unclassified	-0.478	0.365	0.521	-0.409	0	1	1	0	0.77	0.003
**Alps-N**	OTUp_2622	Bacteria	Proteobacteria	Deltaproteobacteria	Myxococcales	Haliangiaceae	Haliangium	uncultured_Nannocystineae_bacterium	-0.323	-0.179	-0.162	0.664	0	0	0	1	0.66	0.024
**Alps-N**	OTUp_2803	Bacteria	Gemmatimonadetes	Gemmatimonadetes	Gemmatimonadales	Gemmatimonadaceae	unclassified	unclassified	-0.232	-0.301	0.673	-0.140	0	0	1	0	0.67	0.033
**Alps-N**	OTUp_2833	Bacteria	Proteobacteria	Betaproteobacteria	Burkholderiales	unclassified	unclassified	unclassified	-0.285	-0.491	0.318	0.458	0	0	1	1	0.67	0.023
**Alps-N**	OTUp_2840	Bacteria	Acidobacteria	Holophagae	Subgroup_7	uncultured_Acidobacteria_bacterium	unclassified	unclassified	-0.391	-0.417	0.165	0.643	0	0	1	1	0.70	0.028
**Alps-N**	OTUp_2872	Bacteria	Acidobacteria	Acidobacteria	Subgroup_4	RB41	uncultured_bacterium	unclassified	-0.413	-0.015	-0.241	0.668	0	0	0	1	0.67	0.027
**Alps-N**	OTUp_2915	Bacteria	Planctomycetes	Planctomycetacia	Planctomycetales	Planctomycetaceae	Singulisphaera	uncultured_bacterium	-0.363	0.090	0.635	-0.363	0	0	1	0	0.63	0.031
**Alps-N**	OTUp_2974	Bacteria	Proteobacteria	Alphaproteobacteria	Rhodospirillales	Acetobacteraceae	Acidiphilium	unclassified	-0.509	-0.415	0.528	0.396	0	0	1	1	0.80	0.005
**Alps-N**	OTUp_2981	Bacteria	Chloroflexi	JG37-AG-4	uncultured_bacterium	unclassified	unclassified	unclassified	-0.744	0.294	0.452	-0.003	0	1	1	1	0.74	0.007
**Alps-N**	OTUp_2991	Bacteria	Proteobacteria	Gammaproteobacteria	Legionellales	Coxiellaceae	Aquicella	bacterium_enrichment_culture_clone_EBa4	-0.396	0.354	-0.396	0.438	0	1	0	1	0.69	0.048
**Alps-N**	OTUp_3011	Bacteria	Planctomycetes	Planctomycetacia	Planctomycetales	Planctomycetaceae	Singulisphaera	uncultured_planctomycete	-0.585	0.525	-0.179	0.240	0	1	0	1	0.66	0.049
**Alps-N**	OTUp_3102	Bacteria	Verrucomicrobia	Spartobacteria	Chthoniobacterales	DA101_soil_group	unclassified	unclassified	-0.495	-0.114	-0.167	0.777	0	0	0	1	0.78	0.008
**Alps-N**	OTUp_3124	Bacteria	Chloroflexi	JG37-AG-4	uncultured_bacterium	unclassified	unclassified	unclassified	-0.563	-0.047	0.708	-0.098	0	0	1	0	0.71	0.008
**Alps-N**	OTUp_3346	Bacteria	Saccharibacteria	unclassified	unclassified	unclassified	unclassified	unclassified	-0.299	0.400	0.419	-0.520	0	1	1	0	0.71	0.019
**Alps-N**	OTUp_3363	Bacteria	Proteobacteria	Deltaproteobacteria	Myxococcales	Polyangiaceae	Sorangium	unclassified	-0.677	0.092	0.530	0.055	0	1	1	1	0.68	0.034
**Alps-N**	OTUp_3447	Bacteria	Actinobacteria	Thermoleophilia	Solirubrobacterales	480-2	uncultured_bacterium	unclassified	-0.492	0.286	0.532	-0.326	0	1	1	0	0.71	0.016
**Alps-N**	OTUp_3508	Bacteria	Planctomycetes	Planctomycetacia	Planctomycetales	Planctomycetaceae	Gemmata	unclassified	-0.517	0.025	-0.211	0.704	0	0	0	1	0.70	0.025
**Alps-N**	OTUp_3533	Bacteria	Acidobacteria	Acidobacteria	Subgroup_4	DS-100	uncultured_Acidobacteria_bacterium	unclassified	-0.465	0.398	0.358	-0.291	0	1	1	0	0.66	0.038
**Alps-N**	OTUp_3603	Bacteria	Gemmatimonadetes	Gemmatimonadetes	Gemmatimonadales	Gemmatimonadaceae	Gemmatimonas	uncultured_bacterium	-0.364	-0.285	-0.032	0.681	0	0	0	1	0.68	0.030
**Alps-N**	OTUp_3608	Bacteria	Planctomycetes	Planctomycetacia	Planctomycetales	Planctomycetaceae	uncultured	uncultured_bacterium	-0.411	-0.411	0.405	0.418	0	0	1	1	0.71	0.028
**Alps-N**	OTUp_3792	Bacteria	Armatimonadetes	unclassified	unclassified	unclassified	unclassified	unclassified	-0.178	-0.347	0.871	-0.347	0	0	1	0	0.87	0.005
**Alps-N**	OTUp_3853	Bacteria	Planctomycetes	Planctomycetacia	Planctomycetales	Planctomycetaceae	Gemmata	unclassified	-0.282	-0.042	-0.296	0.620	0	0	0	1	0.62	0.044
**Alps-N**	OTUp_3859	Bacteria	Proteobacteria	Alphaproteobacteria	Sphingomonadales	Sphingomonadaceae	Sphingomonas	uncultured_alpha_proteobacterium	-0.504	-0.357	0.011	0.851	0	0	0	1	0.85	0.002
**Alps-N**	OTUp_3961	Bacteria	Verrucomicrobia	Spartobacteria	Chthoniobacterales	DA101_soil_group	unclassified	unclassified	-0.339	-0.261	-0.078	0.678	0	0	0	1	0.68	0.020
**Alps-N**	OTUp_4088	Bacteria	unclassified	unclassified	unclassified	unclassified	unclassified	unclassified	-0.357	-0.223	-0.052	0.632	0	0	0	1	0.63	0.039
**Alps-N**	OTUp_4160	Bacteria	Verrucomicrobia	OPB35_soil_group	Unknown_Order	Unknown_Family	Pedosphaera	uncultured_bacterium	-0.354	0.074	0.635	-0.354	0	0	1	0	0.63	0.033
**Alps-N**	OTUp_4194	Bacteria	Planctomycetes	Planctomycetacia	Planctomycetales	Planctomycetaceae	unclassified	unclassified	-0.426	-0.046	-0.286	0.757	0	0	0	1	0.76	0.008
**Alps-N**	OTUp_4199	Bacteria	Actinobacteria	Thermoleophilia	Gaiellales	uncultured	unclassified	unclassified	-0.475	-0.015	-0.283	0.773	0	0	0	1	0.77	0.007
**Alps-N**	OTUp_4217	Bacteria	Planctomycetes	Planctomycetacia	Planctomycetales	Planctomycetaceae	uncultured	uncultured_bacterium	-0.159	0.602	-0.654	0.211	0	1	0	1	0.70	0.011
**Alps-N**	OTUp_4293	Bacteria	Verrucomicrobia	Spartobacteria	Chthoniobacterales	DA101_soil_group	uncultured_bacterium	unclassified	-0.488	-0.142	-0.147	0.777	0	0	0	1	0.78	0.005
**Alps-N**	OTUp_4339	Bacteria	Proteobacteria	Gammaproteobacteria	Legionellales	Coxiellaceae	Coxiella	uncultured_bacterium	-0.309	-0.093	0.712	-0.309	0	0	1	0	0.71	0.028
**Alps-N**	OTUp_4377	Bacteria	Acidobacteria	Acidobacteria	Subgroup_3	Unknown_Family	Bryobacter	unclassified	-0.666	0.598	-0.049	0.117	0	1	1	1	0.67	0.031
**Alps-N**	OTUp_4412	Bacteria	unclassified	unclassified	unclassified	unclassified	unclassified	unclassified	-0.315	-0.068	-0.315	0.698	0	0	0	1	0.70	0.028
**Alps-N**	OTUp_4581	Bacteria	Gemmatimonadetes	Gemmatimonadetes	Gemmatimonadales	Gemmatimonadaceae	uncultured	uncultured_alpha_proteobacterium	-0.239	0.492	0.233	-0.485	0	1	1	0	0.63	0.048
**Alps-N**	OTUp_4600	Bacteria	Verrucomicrobia	Spartobacteria	Chthoniobacterales	DA101_soil_group	uncultured_soil_bacterium	unclassified	-0.404	-0.129	-0.141	0.674	0	0	0	1	0.67	0.045
**Alps-N**	OTUp_4870	Bacteria	Chloroflexi	JG37-AG-4	uncultured_Thermomicrobia_bacterium	unclassified	unclassified	unclassified	-0.332	-0.332	-0.332	0.996	0	0	0	1	1.00	0.002
**Alps-N**	OTUp_4887	Bacteria	Verrucomicrobia	Spartobacteria	Chthoniobacterales	DA101_soil_group	uncultured_soil_bacterium	unclassified	-0.491	0.102	-0.284	0.673	0	0	0	1	0.67	0.026
**Alps-N**	OTUp_4987	Bacteria	Proteobacteria	Deltaproteobacteria	Desulfobacterales	Nitrospinaceae	Candidatus_Entotheonella	uncultured_bacterium	-0.472	0.000	-0.288	0.760	0	0	0	1	0.76	0.002
**Alps-N**	OTUp_5705	Bacteria	Actinobacteria	Acidimicrobiia	Acidimicrobiales	uncultured	uncultured_Aciditerrimonas_sp.	unclassified	-0.594	0.431	0.572	-0.410	0	1	1	0	0.87	0.001
**Alps-N**	OTUp_6336	Bacteria	Acidobacteria	Acidobacteria	Subgroup_4	Unknown_Family	Blastocatella	uncultured_bacterium	-0.469	-0.411	0.463	0.417	0	0	1	1	0.76	0.007
**Alps-N**	OTUp_6596	Bacteria	Proteobacteria	Deltaproteobacteria	Myxococcales	P3OB-42	uncultured_bacterium	unclassified	-0.364	-0.158	0.885	-0.364	0	0	1	0	0.89	0.002
**Alps-N**	OTUp_6715	Bacteria	Actinobacteria	Actinobacteria	Corynebacteriales	Mycobacteriaceae	Mycobacterium	unclassified	-0.170	0.590	0.215	-0.636	0	1	1	0	0.70	0.017
**Alps-N**	OTUp_7177	Bacteria	Bacteroidetes	Sphingobacteriia	Sphingobacteriales	Chitinophagaceae	uncultured	uncultured_bacterium	-0.482	0.224	0.615	-0.357	0	1	1	0	0.73	0.016

**Alps-N**	OTUp_7368	Bacteria	Acidobacteria	Holophagae	Subgroup_7	uncultured_bacterium	unclassified	unclassified	-0.616	0.050	-0.099	0.666	0	0	0	1	0.67	0.033
**Alps-N**	OTUp_7677	Bacteria	Proteobacteria	Betaproteobacteria	Nitrosomonadales	unclassified	unclassified	unclassified	-0.291	-0.291	-0.149	0.732	0	0	0	1	0.73	0.031
**Alps-N**	OTUp_7834	Bacteria	Acidobacteria	Subgroup_22	uncultured_bacterium	unclassified	unclassified	unclassified	-0.260	-0.260	0.780	-0.260	0	0	1	0	0.78	0.027
**Alps-N**	OTUp_7879	Bacteria	Acidobacteria	Holophagae	Subgroup_7	unclassified	unclassified	unclassified	-0.239	-0.684	0.320	0.602	0	0	1	1	0.80	0.002
**Alps-N**	OTUp_8190	Bacteria	Verrucomicrobia	Spartobacteria	Chthoniobacterales	DA101_soil_group	unclassified	unclassified	-0.582	-0.014	-0.134	0.730	0	0	0	1	0.73	0.005
**Alps-N**	OTUp_8559	Bacteria	Chloroflexi	KD4-96	unclassified	unclassified	unclassified	unclassified	-0.640	0.538	-0.057	0.160	0	1	1	1	0.64	0.050
**Alps-N**	OTUp_8575	Bacteria	Verrucomicrobia	Spartobacteria	Chthoniobacterales	DA101_soil_group	uncultured_bacterium	unclassified	-0.405	-0.463	0.224	0.644	0	0	1	1	0.75	0.011
**Alps-N**	OTUp_8777	Bacteria	Chloroflexi	SHA-26	uncultured_bacterium	unclassified	unclassified	unclassified	-0.386	-0.077	-0.218	0.681	0	0	0	1	0.68	0.017
**Alps-N**	OTUp_8778	Bacteria	Cyanobacteria	Melainabacteria	Vampirovibrionales	uncultured_bacterium	unclassified	unclassified	-0.284	-0.284	-0.101	0.669	0	0	0	1	0.67	0.028
**Alps-N**	OTUp_9275	Bacteria	Acidobacteria	Acidobacteria	Subgroup_6	uncultured_Acidobacteria_bacterium	unclassified	unclassified	-0.306	-0.092	0.703	-0.306	0	0	1	0	0.70	0.028
**Alps-N**	OTUp_9280	Bacteria	Proteobacteria	Betaproteobacteria	Burkholderiales	Oxalobacteraceae	Noviherbaspirillum	unclassified	-0.294	-0.237	0.924	-0.393	0	0	1	0	0.92	0.002
**Alps-N**	OTUp_9553	Bacteria	Verrucomicrobia	Spartobacteria	Chthoniobacterales	DA101_soil_group	uncultured_bacterium	unclassified	-0.569	-0.204	0.071	0.702	0	0	0	1	0.70	0.012
**Alps-N**	OTUp_9782	Bacteria	Chloroflexi	JG37-AG-4	uncultured_soil_bacterium	unclassified	unclassified	unclassified	-0.391	-0.276	-0.036	0.704	0	0	0	1	0.70	0.043
**Alps-N**	OTUp_10119	Bacteria	Planctomycetes	Planctomycetacia	Planctomycetales	Planctomycetaceae	uncultured	unclassified	-0.482	0.357	0.526	-0.400	0	1	1	0	0.76	0.007
**Alps-N**	OTUp_10173	Bacteria	Planctomycetes	Planctomycetacia	Planctomycetales	Planctomycetaceae	uncultured	uncultured_Gemmata_sp.	-0.332	-0.032	0.697	-0.332	0	0	1	0	0.70	0.028
**Alps-N**	OTUp_10412	Bacteria	Acidobacteria	Acidobacteria	Subgroup_3	Unknown_Family	Bryobacter	unclassified	-0.579	-0.318	0.456	0.441	0	0	1	1	0.78	0.003
**Alps-N**	OTUp_10434	Bacteria	Verrucomicrobia	Spartobacteria	Chthoniobacterales	DA101_soil_group	unclassified	unclassified	-0.150	-0.365	-0.334	0.849	0	0	0	1	0.85	0.002
**Alps-N**	OTUp_11077	Bacteria	Verrucomicrobia	Spartobacteria	Chthoniobacterales	Chthoniobacteraceae	Chthoniobacter	bacterium_Ellin506	-0.388	0.584	-0.388	0.192	0	1	0	1	0.67	0.029
**Alps-S**	OTUp_1	Bacteria	Proteobacteria	Alphaproteobacteria	Rhizobiales	Bradyrhizobiaceae	Bradyrhizobium	Bradyrhizobium_canariense	-0.350	0.243	-0.509	0.615	0	1	0	1	0.74	0.002
**Alps-S**	OTUp_4	Bacteria	Actinobacteria	Actinobacteria	Propionibacteriales	Nocardioidaceae	Nocardioides	unclassified	0.067	-0.505	0.874	-0.436	0	0	1	0	0.87	0.002
**Alps-S**	OTUp_8	Bacteria	Chloroflexi	JG37-AG-4	uncultured_Clostridium_sp.	unclassified	unclassified	unclassified	-0.555	0.765	0.245	-0.455	0	1	1	0	0.87	0.001
**Alps-S**	OTUp_9	Bacteria	Chloroflexi	JG37-AG-4	uncultured_bacterium	unclassified	unclassified	unclassified	-0.893	0.607	0.007	0.280	0	1	1	1	0.89	0.003
**Alps-S**	OTUp_11	Bacteria	Verrucomicrobia	Spartobacteria	Chthoniobacterales	DA101_soil_group	uncultured_soil_bacterium	unclassified	-0.199	-0.374	-0.218	0.790	0	0	0	1	0.79	0.009
**Alps-S**	OTUp_12	Bacteria	Verrucomicrobia	Spartobacteria	Chthoniobacterales	DA101_soil_group	uncultured_Verrucomicrobia_bacterium	unclassified	-0.156	-0.337	-0.226	0.719	0	0	0	1	0.72	0.019
**Alps-S**	OTUp_13	Bacteria	Acidobacteria	Acidobacteria	Acidobacteriales	Acidobacteriaceae	uncultured	unclassified	-0.014	-0.517	-0.116	0.647	0	0	0	1	0.65	0.043
**Alps-S**	OTUp_15	Bacteria	Proteobacteria	Betaproteobacteria	SC-I-84	uncultured_beta_proteobacterium	unclassified	unclassified	-0.390	-0.246	-0.003	0.639	0	0	0	1	0.64	0.047
**Alps-S**	OTUp_20	Bacteria	Verrucomicrobia	Spartobacteria	Chthoniobacterales	DA101_soil_group	uncultured_Verrucomicrobia_bacterium	unclassified	-0.336	-0.164	-0.178	0.678	0	0	0	1	0.68	0.045
**Alps-S**	OTUp_26	Bacteria	Proteobacteria	Gammaproteobacteria	Xanthomonadales	Xanthomonadales_Incertae_Sedis	Acidibacter	uncultured_gamma_proteobacterium	-0.844	-0.004	0.422	0.426	0	1	1	1	0.84	0.003
**Alps-S**	OTUp_27	Bacteria	Chloroflexi	JG37-AG-4	uncultured_bacterium	unclassified	unclassified	unclassified	-0.692	0.660	-0.005	0.037	0	1	1	1	0.69	0.027
**Alps-S**	OTUp_28	Bacteria	Proteobacteria	Gammaproteobacteria	Xanthomonadales	uncultured	uncultured_eubacterium_WD260	unclassified	-0.716	-0.291	0.568	0.438	0	0	1	1	0.87	0.001
**Alps-S**	OTUp_29	Bacteria	Chloroflexi	JG37-AG-4	uncultured_bacterium	unclassified	unclassified	unclassified	-0.584	0.568	0.245	-0.229	0	1	1	0	0.70	0.025
**Alps-S**	OTUp_31	Bacteria	Actinobacteria	Actinobacteria	Micrococcales	Micrococcaceae	Arthrobacter	unclassified	-0.107	-0.446	0.965	-0.412	0	0	1	0	0.96	0.001
**Alps-S**	OTUp_34	Bacteria	Proteobacteria	Gammaproteobacteria	Xanthomonadales	Xanthomonadaceae	Rhodanobacter	uncultured_Xanthomonadaceae_bacterium	-0.473	-0.280	0.058	0.695	0	0	0	1	0.69	0.017
**Alps-S**	OTUp_35	Bacteria	Planctomycetes	Planctomycetacia	Planctomycetales	Planctomycetaceae	uncultured	unclassified	-0.716	0.523	0.118	0.076	0	1	1	1	0.72	0.013
**Alps-S**	OTUp_36	Bacteria	Verrucomicrobia	Spartobacteria	Chthoniobacterales	DA101_soil_group	uncultured_Prosthecobacter_sp.	unclassified	-0.348	-0.380	-0.099	0.827	0	0	0	1	0.83	0.003
**Alps-S**	OTUp_43	Bacteria	Acidobacteria	Acidobacteria	Subgroup_3	Unknown_Family	Bryobacter	uncultured_Acidobacteria_bacterium	-0.471	-0.315	-0.048	0.834	0	0	0	1	0.83	0.002
**Alps-S**	OTUp_46	Bacteria	WD272	uncultured_Firmicutes_bacterium	unclassified	unclassified	unclassified	unclassified	-0.455	-0.208	-0.094	0.757	0	0	0	1	0.76	0.015
**Alps-S**	OTUp_47	Bacteria	Planctomycetes	Planctomycetacia	Planctomycetales	Planctomycetaceae	uncultured	unclassified	-0.833	0.297	0.117	0.419	0	1	1	1	0.83	0.002
**Alps-S**	OTUp_48	Bacteria	Verrucomicrobia	Spartobacteria	Chthoniobacterales	DA101_soil_group	uncultured_bacterium	unclassified	-0.412	-0.207	-0.153	0.772	0	0	0	1	0.77	0.007
**Alps-S**	OTUp_50	Bacteria	WD272	unclassified	unclassified	unclassified	unclassified	unclassified	-0.520	-0.145	-0.205	0.869	0	0	0	1	0.87	0.003
**Alps-S**	OTUp_51	Bacteria	Bacteroidetes	Sphingobacteriia	Sphingobacteriales	Chitinophagaceae	uncultured	unclassified	-0.245	-0.522	0.032	0.735	0	0	0	1	0.73	0.013
**Alps-S**	OTUp_61	Bacteria	Verrucomicrobia	Spartobacteria	Chthoniobacterales	DA101_soil_group	uncultured_Spartobacteria_bacterium	unclassified	-0.325	-0.176	-0.178	0.679	0	0	0	1	0.68	0.032
**Alps-S**	OTUp_64	Bacteria	Proteobacteria	Alphaproteobacteria	Rhodospirillales	Acetobacteraceae	unclassified	unclassified	-0.337	0.313	0.536	-0.512	0	1	1	0	0.74	0.014
**Alps-S**	OTUp_67	Bacteria	Actinobacteria	Acidimicrobiia	Acidimicrobiales	uncultured	uncultured_Ferrimicrobium_sp.	unclassified	-0.583	0.564	0.215	-0.196	0	1	1	0	0.67	0.030
**Alps-S**	OTUp_73	Bacteria	WD272	uncultured_bacterium	unclassified	unclassified	unclassified	unclassified	-0.266	-0.353	-0.255	0.874	0	0	0	1	0.87	0.003
**Alps-S**	OTUp_76	Bacteria	Proteobacteria	Betaproteobacteria	Burkholderiales	Oxalobacteraceae	Noviherbaspirillum	uncultured_bacterium	0.074	-0.584	0.886	-0.377	0	0	1	0	0.89	0.002
**Alps-S**	OTUp_82	Bacteria	WD272	uncultured_Thermoanaerobacteraceae_bacterium	unclassified	unclassified	unclassified	unclassified	-0.434	-0.155	-0.124	0.713	0	0	0	1	0.71	0.005
**Alps-S**	OTUp_83	Bacteria	WD272	uncultured_bacterium	unclassified	unclassified	unclassified	unclassified	-0.705	0.036	0.288	0.382	0	1	1	1	0.71	0.009
**Alps-S**	OTUp_86	Bacteria	Chloroflexi	JG37-AG-4	uncultured_bacterium	unclassified	unclassified	unclassified	-0.718	0.655	-0.117	0.180	0	1	0	1	0.72	0.016
**Alps-S**	OTUp_89	Bacteria	Chloroflexi	unclassified	unclassified	unclassified	unclassified	unclassified	-0.722	0.556	0.031	0.135	0	1	1	1	0.72	0.010
**Alps-S**	OTUp_90	Bacteria	Planctomycetes	Planctomycetacia	Planctomycetales	Planctomycetaceae	uncultured	uncultured_bacterium	-0.411	-0.335	0.158	0.588	0	0	1	1	0.65	0.044
**Alps-S**	OTUp_94	Bacteria	Actinobacteria	Actinobacteria	Corynebacteriales	Mycobacteriaceae	Mycobacterium	unclassified	-0.126	-0.118	0.862	-0.618	0	0	1	0	0.86	0.002
**Alps-S**	OTUp_99	Bacteria	Proteobacteria	Alphaproteobacteria	Rhodospirillales	Rhodospirillales_Incertae_Sedis	Reyranella	uncultured_alpha_proteobacterium	-0.528	-0.220	0.050	0.699	0	0	0	1	0.70	0.009
**Alps-S**	OTUp_101	Bacteria	Proteobacteria	Alphaproteobacteria	Rhizobiales	MNG7	uncultured_Phyllobacteriaceae_bacterium	unclassified	0.015	-0.510	-0.239	0.734	0	0	0	1	0.73	0.016
**Alps-S**	OTUp_106	Bacteria	Proteobacteria	Alphaproteobacteria	Rhizobiales	Rhizobiales_Incertae_Sedis	Rhizomicrobium	uncultured_alpha_proteobacterium	-0.277	-0.233	-0.347	0.858	0	0	0	1	0.86	0.003
**Alps-S**	OTUp_107	Bacteria	Planctomycetes	Planctomycetacia	Planctomycetales	Planctomycetaceae	uncultured	uncultured_Gemmata_sp.	-0.596	-0.176	0.216	0.555	0	0	1	1	0.67	0.028
**Alps-S**	OTUp_108	Bacteria	Planctomycetes	Planctomycetacia	Planctomycetales	Planctomycetaceae	Gemmata	uncultured_bacterium	-0.284	-0.127	-0.257	0.668	0	0	0	1	0.67	0.013
**Alps-S**	OTUp_109	Bacteria	Proteobacteria	Betaproteobacteria	Nitrosomonadales	Nitrosomonadaceae	uncultured	uncultured_Oxalobacteraceae_bacterium	-0.651	0.259	-0.287	0.679	0	1	0	1	0.81	0.003
**Alps-S**	OTUp_110	Bacteria	Planctomycetes	Planctomycetacia	Planctomycetales	Planctomycetaceae	uncultured	uncultured_bacterium	-0.643	0.357	-0.249	0.536	0	1	0	1	0.77	0.005
**Alps-S**	OTUp_112	Bacteria	Proteobacteria	Alphaproteobacteria	Caulobacterales	Caulobacteraceae	uncultured	uncultured_bacterium	-0.660	-0.107	0.228	0.540	0	0	1	1	0.66	0.031
**Alps-S**	OTUp_113	Bacteria	Proteobacteria	Alphaproteobacteria	Sphingomonadales	Sphingomonadaceae	Sphingomonas	unclassified	-0.515	-0.319	0.189	0.645	0	0	1	1	0.72	0.017
**Alps-S**	OTUp_114	Bacteria	Proteobacteria	Betaproteobacteria	Burkholderiales	unclassified	unclassified	unclassified	-0.431	-0.388	0.170	0.649	0	0	1	1	0.71	0.017
**Alps-S**	OTUp_123	Bacteria	Proteobacteria	Alphaproteobacteria	Rhizobiales	Xanthobacteraceae	Variibacter	uncultured_bacterium	-0.261	0.551	-0.686	0.396	0	1	0	1	0.82	0.004
**Alps-S**	OTUp_125	Bacteria	Planctomycetes	Planctomycetacia	Planctomycetales	Planctomycetaceae	unclassified	unclassified	-0.771	0.329	0.252	0.190	0	1	1	1	0.77	0.004
**Alps-S**	OTUp_127	Bacteria	Acidobacteria	Holophagae	Subgroup_7	uncultured_proteobacterium	unclassified	unclassified	-0.844	0.456	0.331	0.057	0	1	1	1	0.84	0.003
**Alps-S**	OTUp_130	Bacteria	Planctomycetes	Planctomycetacia	Planctomycetales	Planctomycetaceae	Gemmata	uncultured_bacterium	-0.651	-0.008	0.000	0.659	0	0	0	1	0.66	0.044
**Alps-S**	OTUp_133	Bacteria	Planctomycetes	Planctomycetacia	Planctomycetales	Planctomycetaceae	uncultured	unclassified	-0.117	-0.260	-0.326	0.704	0	0	0	1	0.70	0.022
**Alps-S**	OTUp_134	Bacteria	Verrucomicrobia	Spartobacteria	Chthoniobacterales	DA101_soil_group	unclassified	unclassified	-0.508	-0.058	-0.251	0.817	0	0	0	1	0.82	0.003
**Alps-S**	OTUp_137	Bacteria	Planctomycetes	Planctomycetacia	Planctomycetales	Planctomycetaceae	uncultured	unclassified	-0.818	0.456	0.073	0.289	0	1	1	1	0.82	0.004
**Alps-S**	OTUp_138	Bacteria	Proteobacteria	Gammaproteobacteria	Xanthomonadales	Xanthomonadaceae	unclassified	unclassified	-0.570	-0.085	-0.008	0.664	0	0	0	1	0.66	0.035
**Alps-S**	OTUp_141	Bacteria	Actinobacteria	Acidimicrobiia	Acidimicrobiales	Acidimicrobiaceae	uncultured	uncultured_bacterium	-0.687	0.550	-0.063	0.200	0	1	1	1	0.69	0.028
**Alps-S**	OTUp_149	Bacteria	Acidobacteria	Acidobacteria	Subgroup_3	Unknown_Family	Candidatus_Solibacter	uncultured_Acidobacteria_bacterium	-0.410	-0.134	-0.283	0.827	0	0	0	1	0.83	0.005
**Alps-S**	OTUp_150	Bacteria	Actinobacteria	Actinobacteria	Streptomycetales	Streptomycetaceae	Streptacidiphilus	unclassified	0.055	-0.102	0.665	-0.617	0	0	1	0	0.66	0.034
**Alps-S**	OTUp_151	Bacteria	Proteobacteria	Gammaproteobacteria	Xanthomonadales	uncultured	uncultured_proteobacterium	unclassified	-0.453	0.228	-0.283	0.508	0	1	0	1	0.64	0.049
**Alps-S**	OTUp_158	Bacteria	Proteobacteria	Betaproteobacteria	Nitrosomonadales	Nitrosomonadaceae	uncultured	unclassified	-0.711	0.615	-0.022	0.118	0	1	1	1	0.71	0.020
**Alps-S**	OTUp_172	Bacteria	Proteobacteria	Alphaproteobacteria	Sphingomonadales	Sphingomonadaceae	Sphingomonas	Sphingomonas_faeni	-0.379	-0.416	0.439	0.356	0	0	1	1	0.69	0.019
**Alps-S**	OTUp_175	Bacteria	Planctomycetes	Planctomycetacia	Planctomycetales	Planctomycetaceae	uncultured	unclassified	-0.501	0.548	0.197	-0.244	0	1	1	0	0.65	0.036
**Alps-S**	OTUp_177	Bacteria	Verrucomicrobia	Spartobacteria	Chthoniobacterales	DA101_soil_group	uncultured_soil_bacterium	unclassified	-0.113	-0.351	-0.299	0.763	0	0	0	1	0.76	0.014
**Alps-S**	OTUp_188	Bacteria	Verrucomicrobia	Spartobacteria	Chthoniobacterales	DA101_soil_group	uncultured_Verrucomicrobia_bacterium	unclassified	-0.168	-0.260	-0.325	0.754	0	0	0	1	0.75	0.003
**Alps-S**	OTUp_194	Bacteria	Acidobacteria	Acidobacteria	Acidobacteriales	Acidobacteriaceae	uncultured	uncultured_bacterium	-0.101	-0.460	-0.089	0.650	0	0	0	1	0.65	0.046
**Alps-S**	OTUp_199	Bacteria	Planctomycetes	Planctomycetacia	Planctomycetales	Planctomycetaceae	uncultured	uncultured_Planctomycetia_bacterium	-0.575	-0.041	-0.112	0.729	0	0	0	1	0.73	0.007
**Alps-S**	OTUp_205	Bacteria	Acidobacteria	Acidobacteria	Subgroup_4	RB41	unclassified	unclassified	-0.291	-0.277	-0.256	0.824	0	0	0	1	0.82	0.005
**Alps-S**	OTUp_210	Bacteria	Acidobacteria	Acidobacteria	Subgroup_6	unclassified	unclassified	unclassified	-0.833	0.417	0.028	0.388	0	1	1	1	0.83	0.000
**Alps-S**	OTUp_214	Bacteria	Actinobacteria	Thermoleophilia	Solirubrobacterales	TM146	uncultured_Conexibacteraceae_bacterium	unclassified	-0.641	0.606	0.195	-0.160	0	1	1	0	0.69	0.026
**Alps-S**	OTUp_215	Bacteria	Proteobacteria	Alphaproteobacteria	Rhodospirillales	DA111	uncultured_bacterium	unclassified	-0.722	0.320	0.193	0.210	0	1	1	1	0.72	0.010
**Alps-S**	OTUp_216	Bacteria	Proteobacteria	Alphaproteobacteria	Rhizobiales	Xanthobacteraceae	Variibacter	uncultured_Hyphomicrobiaceae_bacterium	-0.356	0.464	-0.367	0.260	0	1	0	1	0.63	0.048
**Alps-S**	OTUp_218	Bacteria	Proteobacteria	Alphaproteobacteria	Rhizobiales	unclassified	unclassified	unclassified	-0.655	0.573	0.104	-0.022	0	1	1	1	0.66	0.042
**Alps-S**	OTUp_222	Bacteria	Planctomycetes	Planctomycetacia	Planctomycetales	Planctomycetaceae	Gemmata	uncultured_planctomycete	-0.352	-0.262	-0.198	0.812	0	0	0	1	0.81	0.003
**Alps-S**	OTUp_228	Bacteria	Proteobacteria	Alphaproteobacteria	Sphingomonadales	Sphingomonadaceae	Sphingomonas	unclassified	-0.679	-0.260	0.288	0.651	0	0	1	1	0.81	0.001
**Alps-S**	OTUp_237	Bacteria	Bacteroidetes	Sphingobacteriia	Sphingobacteriales	Chitinophagaceae	uncultured	unclassified	-0.398	-0.276	-0.021	0.695	0	0	0	1	0.70	0.024
**Alps-S**	OTUp_239	Bacteria	Nitrospirae	Nitrospira	Nitrospirales	Nitrospiraceae	Nitrospira	uncultured_Nitrospira_sp.	-0.285	-0.541	0.233	0.593	0	0	1	1	0.72	0.011
**Alps-S**	OTUp_244	Bacteria	Planctomycetes	Planctomycetacia	Planctomycetales	Planctomycetaceae	Gemmata	uncultured_bacterium	-0.804	0.237	0.159	0.407	0	1	1	1	0.80	0.003
**Alps-S**	OTUp_251	Bacteria	WD272	uncultured_bacterium	unclassified	unclassified	unclassified	unclassified	-0.358	-0.496	0.201	0.653	0	0	1	1	0.74	0.008
**Alps-S**	OTUp_252	Bacteria	Proteobacteria	Alphaproteobacteria	Rhizobiales	Hyphomicrobiaceae	Hyphomicrobium	uncultured_bacterium	-0.519	0.164	0.681	-0.326	0	1	1	0	0.73	0.015
**Alps-S**	OTUp_259	Bacteria	Bacteroidetes	Sphingobacteriia	Sphingobacteriales	Chitinophagaceae	uncultured	uncultured_soil_bacterium	-0.096	-0.171	-0.315	0.583	0	0	0	1	0.58	0.044
**Alps-S**	OTUp_264	Bacteria	Acidobacteria	Acidobacteria	Subgroup_2	uncultured_bacterium	unclassified	unclassified	-0.211	-0.325	-0.195	0.732	0	0	0	1	0.73	0.010

**Alps-S**	OTUp_272	Bacteria	Proteobacteria	Alphaproteobacteria	Rhodospirillales	Acetobacteraceae	Acidiphilium	uncultured_bacterium	-0.456	0.473	0.434	-0.451	0	1	1	0	0.79 0.007
**Alps-S**	OTUp_273	Bacteria	Proteobacteria	Gammaproteobacteria	Xanthomonadales	Xanthomonadales_Incertae_Sedis	Acidibacter	unclassified	-0.683	0.117	0.038	0.527	0	1	1	1	0.68 0.023
**Alps-S**	OTUp_280	Bacteria	Actinobacteria	Actinobacteria	Propionibacteriales	Nocardioidaceae	Aeromicrobium	unclassified	-0.362	-0.068	0.818	-0.388	0	0	1	0	0.82 0.005
**Alps-S**	OTUp_290	Bacteria	Proteobacteria	Alphaproteobacteria	Rhodospirillales	Rhodospirillales_Incertae_Sedis	Reyranella	uncultured_alpha_proteobacterium	-0.680	0.546	-0.049	0.183	0	1	1	1	0.68 0.030
**Alps-S**	OTUp_300	Bacteria	Planctomycetes	Phycisphaerae	WD2101_soil_group	uncultured_eubacterium_WD2101	unclassified	unclassified	-0.234	-0.196	-0.351	0.781	0	0	0	1	0.78 0.004
**Alps-S**	OTUp_311	Bacteria	Proteobacteria	Alphaproteobacteria	Rhizobiales	Hyphomicrobiaceae	Pedomicrobium	uncultured_bacterium	-0.705	0.422	0.011	0.272	0	1	1	1	0.71 0.011
**Alps-S**	OTUp_319	Bacteria	Proteobacteria	Gammaproteobacteria	Xanthomonadales	Xanthomonadales_Incertae_Sedis	uncultured	uncultured_bacterium	-0.781	0.326	0.382	0.073	0	1	1	1	0.78 0.005
**Alps-S**	OTUp_341	Bacteria	Acidobacteria	Acidobacteria	Acidobacteriales	Acidobacteriaceae	Granulicella	uncultured_Acidobacteria_bacterium	-0.570	-0.191	0.155	0.606	0	0	1	1	0.66 0.028
**Alps-S**	OTUp_379	Bacteria	Acidobacteria	Acidobacteria	Subgroup_4	Unknown_Family	Blastocatella	uncultured_Acidobacteria_bacterium	-0.152	-0.343	-0.362	0.857	0	0	0	1	0.86 0.005
**Alps-S**	OTUp_381	Bacteria	Chloroflexi	JG37-AG-4	unclassified	unclassified	unclassified	unclassified	-0.269	0.351	0.480	-0.561	0	1	1	0	0.72 0.011
**Alps-S**	OTUp_384	Bacteria	WD272	uncultured_Firmicutes_bacterium	unclassified	unclassified	unclassified	unclassified	-0.697	0.075	0.200	0.422	0	1	1	1	0.70 0.017
**Alps-S**	OTUp_385	Bacteria	Verrucomicrobia	Spartobacteria	Chthoniobacterales	DA101_soil_group	uncultured_Verrucomicrobia_bacterium	unclassified	-0.251	-0.303	-0.186	0.740	0	0	0	1	0.74 0.012
**Alps-S**	OTUp_387	Bacteria	Proteobacteria	Betaproteobacteria	TRA3-20	unclassified	unclassified	unclassified	-0.267	-0.140	-0.290	0.697	0	0	0	1	0.70 0.028
**Alps-S**	OTUp_390	Bacteria	Chloroflexi	JG37-AG-4	uncultured_bacterium	unclassified	unclassified	unclassified	-0.530	0.586	0.286	-0.342	0	1	1	0	0.76 0.007
**Alps-S**	OTUp_391	Bacteria	Acidobacteria	Acidobacteria	Subgroup_6	unclassified	unclassified	unclassified	-0.838	0.410	0.041	0.387	0	1	1	1	0.84 0.000
**Alps-S**	OTUp_416	Bacteria	Planctomycetes	Planctomycetacia	Planctomycetales	Planctomycetaceae	uncultured	unclassified	-0.424	-0.142	-0.167	0.733	0	0	0	1	0.73 0.011
**Alps-S**	OTUp_420	Bacteria	Verrucomicrobia	Opitutae	Opitutales	Opitutaceae	Opitutus	uncultured_Verrucomicrobia_bacterium	-0.260	-0.102	-0.341	0.702	0	0	0	1	0.70 0.017
**Alps-S**	OTUp_424	Bacteria	Proteobacteria	Betaproteobacteria	Burkholderiales	unclassified	unclassified	unclassified	-0.757	0.510	0.315	-0.068	0	1	1	1	0.76 0.007
**Alps-S**	OTUp_442	Bacteria	Proteobacteria	Gammaproteobacteria	Xanthomonadales	Xanthomonadaceae	Arenimonas	unclassified	-0.305	-0.226	-0.283	0.813	0	0	0	1	0.81 0.003
**Alps-S**	OTUp_456	Bacteria	Acidobacteria	Acidobacteria	Subgroup_3	Unknown_Family	Bryobacter	uncultured_bacterium	-0.447	-0.258	0.045	0.660	0	0	0	1	0.66 0.032
**Alps-S**	OTUp_465	Bacteria	Acidobacteria	Acidobacteria	Subgroup_4	Unknown_Family	Blastocatella	uncultured_bacterium_gp4	-0.307	-0.389	0.024	0.673	0	0	0	1	0.67 0.027
**Alps-S**	OTUp_474	Bacteria	Proteobacteria	Alphaproteobacteria	Sphingomonadales	Sphingomonadaceae	Sphingomonas	Sphingomonas_wittichii	-0.432	-0.132	-0.093	0.658	0	0	0	1	0.66 0.033
**Alps-S**	OTUp_478	Bacteria	Planctomycetes	Planctomycetacia	Planctomycetales	Planctomycetaceae	Planctomyces	unclassified	-0.530	-0.388	0.404	0.515	0	0	1	1	0.80 0.004
**Alps-S**	OTUp_480	Bacteria	Planctomycetes	Planctomycetacia	Planctomycetales	Planctomycetaceae	uncultured	uncultured_bacterium	-0.525	-0.208	0.179	0.554	0	0	1	1	0.64 0.045
**Alps-S**	OTUp_484	Bacteria	Actinobacteria	Actinobacteria	Micrococcales	Micrococcaceae	Arthrobacter	unclassified	-0.131	-0.533	0.927	-0.263	0	0	1	0	0.93 0.002
**Alps-S**	OTUp_486	Bacteria	Verrucomicrobia	OPB35_soil_group	unclassified	unclassified	unclassified	unclassified	-0.360	-0.336	-0.053	0.748	0	0	0	1	0.75 0.012
**Alps-S**	OTUp_491	Bacteria	SM2F11	uncultured_bacterium	unclassified	unclassified	unclassified	unclassified	-0.261	-0.195	-0.389	0.845	0	0	0	1	0.84 0.003
**Alps-S**	OTUp_495	Bacteria	Proteobacteria	Alphaproteobacteria	Rhodospirillales	DA111	uncultured_alpha_proteobacterium	unclassified	-0.367	0.089	-0.402	0.680	0	0	0	1	0.68 0.028
**Alps-S**	OTUp_510	Bacteria	Proteobacteria	Alphaproteobacteria	Rhodospirillales	Rhodospirillales_Incertae_Sedis	Reyranella	uncultured_bacterium	-0.677	0.170	0.468	0.039	0	1	1	1	0.68 0.034
**Alps-S**	OTUp_517	Bacteria	Chlorobi	Chlorobia	Chlorobiales	SJA-28	uncultured_bacterium	unclassified	-0.345	-0.121	-0.257	0.723	0	0	0	1	0.72 0.013
**Alps-S**	OTUp_524	Bacteria	Verrucomicrobia	OPB35_soil_group	uncultured_bacterium	unclassified	unclassified	unclassified	-0.779	0.111	0.314	0.353	0	1	1	1	0.78 0.006
**Alps-S**	OTUp_541	Bacteria	Proteobacteria	Betaproteobacteria	Burkholderiales	Comamonadaceae	unclassified	unclassified	-0.469	-0.142	0.828	-0.217	0	0	1	0	0.83 0.000
**Alps-S**	OTUp_542	Bacteria	Planctomycetes	Phycisphaerae	WD2101_soil_group	uncultured_Planctomycetaceae_bacterium	unclassified	unclassified	-0.358	-0.165	-0.327	0.850	0	0	0	1	0.85 0.003
**Alps-S**	OTUp_560	Bacteria	Actinobacteria	Thermoleophilia	Solirubrobacterales	unclassified	unclassified	unclassified	-0.574	0.587	0.220	-0.233	0	1	1	0	0.70 0.021
**Alps-S**	OTUp_567	Bacteria	Verrucomicrobia	Spartobacteria	Chthoniobacterales	Chthoniobacteraceae	Chthoniobacter	uncultured_Verrucomicrobia_bacterium	-0.337	0.294	-0.413	0.456	0	1	0	1	0.65 0.048
**Alps-S**	OTUp_570	Bacteria	WD272	unclassified	unclassified	unclassified	unclassified	unclassified	-0.415	-0.166	-0.239	0.820	0	0	0	1	0.82 0.003
**Alps-S**	OTUp_582	Bacteria	Acidobacteria	Acidobacteria	Subgroup_6	uncultured_bacterium_270	unclassified	unclassified	-0.643	0.342	-0.081	0.382	0	1	1	1	0.64 0.043
**Alps-S**	OTUp_586	Bacteria	Proteobacteria	Betaproteobacteria	unclassified	unclassified	unclassified	unclassified	-0.431	-0.242	-0.233	0.906	0	0	0	1	0.91 0.003
**Alps-S**	OTUp_588	Bacteria	SM2F11	uncultured_bacterium	unclassified	unclassified	unclassified	unclassified	-0.170	0.056	-0.555	0.669	0	0	0	1	0.67 0.034
**Alps-S**	OTUp_596	Bacteria	Chloroflexi	Ktedonobacteria	C0119	uncultured_bacterium	unclassified	unclassified	-0.513	0.385	0.462	-0.335	0	1	1	0	0.73 0.011
**Alps-S**	OTUp_600	Bacteria	Proteobacteria	Deltaproteobacteria	GR-WP33-30	uncultured_bacterium	unclassified	unclassified	-0.424	-0.294	-0.017	0.735	0	0	0	1	0.74 0.012
**Alps-S**	OTUp_602	Bacteria	Planctomycetes	Planctomycetacia	Planctomycetales	Planctomycetaceae	uncultured	unclassified	-0.498	0.671	0.107	-0.280	0	1	1	0	0.67 0.037
**Alps-S**	OTUp_621	Bacteria	Chloroflexi	uncultured	unclassified	unclassified	unclassified	unclassified	-0.365	0.619	-0.479	0.225	0	1	0	1	0.73 0.010
**Alps-S**	OTUp_625	Bacteria	Verrucomicrobia	OPB35_soil_group	unclassified	unclassified	unclassified	unclassified	-0.149	-0.066	-0.535	0.750	0	0	0	1	0.75 0.003
**Alps-S**	OTUp_637	Bacteria	Planctomycetes	Planctomycetacia	Planctomycetales	Planctomycetaceae	Singulisphaera	unclassified	-0.692	0.460	0.024	0.208	0	1	1	1	0.69 0.023
**Alps-S**	OTUp_640	Bacteria	Chloroflexi	Anaerolineae	Anaerolineales	Anaerolineaceae	uncultured	unclassified	-0.182	-0.237	-0.182	0.601	0	0	0	1	0.60 0.050
**Alps-S**	OTUp_648	Bacteria	Planctomycetes	Planctomycetacia	Planctomycetales	Planctomycetaceae	uncultured	unclassified	-0.240	0.523	-0.551	0.269	0	1	0	1	0.69 0.029
**Alps-S**	OTUp_672	Bacteria	Proteobacteria	Betaproteobacteria	SC-I-84	unclassified	unclassified	unclassified	-0.445	-0.282	0.502	0.225	0	0	1	1	0.63 0.047
**Alps-S**	OTUp_677	Bacteria	Verrucomicrobia	Spartobacteria	Chthoniobacterales	DA101_soil_group	uncultured_Verrucomicrobia_bacterium	unclassified	-0.405	-0.284	-0.127	0.816	0	0	0	1	0.82 0.003
**Alps-S**	OTUp_681	Bacteria	Actinobacteria	Acidimicrobiia	Acidimicrobiales	uncultured	uncultured_bacterium	unclassified	-0.496	0.396	-0.256	0.356	0	1	0	1	0.65 0.046
**Alps-S**	OTUp_682	Bacteria	Proteobacteria	Alphaproteobacteria	Sphingomonadales	Sphingomonadaceae	Sphingomonas	unclassified	-0.619	-0.185	0.045	0.760	0	0	0	1	0.76 0.010
**Alps-S**	OTUp_690	Bacteria	Armatimonadetes	uncultured_bacterium	unclassified	unclassified	unclassified	unclassified	-0.487	-0.098	-0.165	0.749	0	0	0	1	0.75 0.008
**Alps-S**	OTUp_691	Bacteria	Verrucomicrobia	Spartobacteria	Chthoniobacterales	Chthoniobacteraceae	Chthoniobacter	uncultured_Verrucomicrobia_bacterium	-0.321	0.332	-0.524	0.513	0	1	0	1	0.73 0.001
**Alps-S**	OTUp_695	Bacteria	Verrucomicrobia	Spartobacteria	Chthoniobacterales	DA101_soil_group	unclassified	unclassified	-0.393	-0.262	-0.269	0.924	0	0	0	1	0.92 0.003
**Alps-S**	OTUp_718	Bacteria	unclassified	unclassified	unclassified	unclassified	unclassified	unclassified	-0.382	0.214	0.549	-0.382	0	1	1	0	0.66 0.044
**Alps-S**	OTUp_724	Bacteria	Bacteroidetes	Sphingobacteriia	Sphingobacteriales	Chitinophagaceae	Ferruginibacter	unclassified	0.005	-0.315	-0.333	0.643	0	0	0	1	0.64 0.049
**Alps-S**	OTUp_730	Bacteria	WD272	uncultured_cyanobacterium	unclassified	unclassified	unclassified	unclassified	-0.220	-0.223	-0.195	0.638	0	0	0	1	0.64 0.049
**Alps-S**	OTUp_737	Bacteria	Bacteroidetes	Sphingobacteriia	Sphingobacteriales	Sphingobacteriaceae	Mucilaginibacter	uncultured_Mucilaginibacter_sp.	-0.136	-0.329	-0.280	0.744	0	0	0	1	0.74 0.012
**Alps-S**	OTUp_751	Bacteria	Chloroflexi	JG37-AG-4	uncultured_bacterium	unclassified	unclassified	unclassified	-0.627	0.490	-0.255	0.392	0	1	0	1	0.76 0.007
**Alps-S**	OTUp_756	Bacteria	Proteobacteria	Betaproteobacteria	Burkholderiales	Comamonadaceae	unclassified	unclassified	-0.438	-0.247	0.801	-0.116	0	0	1	0	0.80 0.002
**Alps-S**	OTUp_768	Bacteria	Proteobacteria	Alphaproteobacteria	Rhizobiales	Xanthobacteraceae	Pseudolabrys	uncultured_bacterium	-0.673	0.397	-0.088	0.364	0	1	1	1	0.67 0.028
**Alps-S**	OTUp_772	Bacteria	Planctomycetes	Planctomycetacia	Planctomycetales	Planctomycetaceae	Gemmata	uncultured_planctomycete	-0.330	-0.092	-0.274	0.696	0	0	0	1	0.70 0.011
**Alps-S**	OTUp_773	Bacteria	Proteobacteria	Gammaproteobacteria	Xanthomonadales	Xanthomonadaceae	uncultured	unclassified	-0.340	-0.019	-0.372	0.731	0	0	0	1	0.73 0.013
**Alps-S**	OTUp_784	Bacteria	Proteobacteria	Deltaproteobacteria	Myxococcales	Cystobacteraceae	Anaeromyxobacter	uncultured_bacterium	-0.662	0.722	0.200	-0.260	0	1	1	0	0.80 0.003
**Alps-S**	OTUp_804	Bacteria	Chloroflexi	JG37-AG-4	uncultured_bacterium	unclassified	unclassified	unclassified	-0.522	0.384	-0.244	0.382	0	1	0	1	0.66 0.043
**Alps-S**	OTUp_805	Bacteria	Gemmatimonadetes	Gemmatimonadetes	Gemmatimonadales	Gemmatimonadaceae	unclassified	unclassified	-0.378	-0.010	-0.283	0.672	0	0	0	1	0.67 0.028
**Alps-S**	OTUp_821	Bacteria	Thermotogae	Thermotogae	Thermotogales	Thermotogaceae	GAL15	uncultured_Firmicutes_bacterium	-0.666	0.609	0.146	-0.090	0	1	1	1	0.67 0.038
**Alps-S**	OTUp_829	Bacteria	Actinobacteria	Acidimicrobiia	Acidimicrobiales	uncultured	uncultured_Aciditerrimonas_sp.	unclassified	-0.658	0.329	-0.086	0.415	0	1	1	1	0.66 0.047
**Alps-S**	OTUp_830	Bacteria	Planctomycetes	Planctomycetacia	Planctomycetales	Planctomycetaceae	Singulisphaera	uncultured_bacterium	-0.582	0.472	-0.227	0.337	0	1	0	1	0.70 0.018
**Alps-S**	OTUp_831	Bacteria	Proteobacteria	Deltaproteobacteria	Myxococcales	Polyangiaceae	Sorangium	uncultured_bacterium	-0.653	0.321	0.246	0.085	0	1	1	1	0.65 0.032
**Alps-S**	OTUp_834	Bacteria	Proteobacteria	Deltaproteobacteria	Myxococcales	Cystobacteraceae	Anaeromyxobacter	uncultured_Anaeromyxobacter_sp.	-0.670	0.385	0.092	0.193	0	1	1	1	0.67 0.025
**Alps-S**	OTUp_836	Bacteria	WD272	uncultured_Firmicutes_bacterium	unclassified	unclassified	unclassified	unclassified	-0.204	-0.245	-0.421	0.870	0	0	0	1	0.87 0.001
**Alps-S**	OTUp_848	Bacteria	Proteobacteria	Alphaproteobacteria	Caulobacterales	Caulobacteraceae	Phenylobacterium	unclassified	-0.332	-0.420	0.461	0.291	0	0	1	1	0.65 0.031
**Alps-S**	OTUp_860	Bacteria	Chloroflexi	JG37-AG-4	unclassified	unclassified	unclassified	unclassified	-0.677	0.556	-0.147	0.269	0	1	0	1	0.71 0.017
**Alps-S**	OTUp_876	Bacteria	Actinobacteria	Acidimicrobiia	Acidimicrobiales	Iamiaceae	Iamia	uncultured_bacterium	-0.655	0.633	0.031	-0.009	0	1	1	1	0.65 0.043
**Alps-S**	OTUp_888	Bacteria	Actinobacteria	Acidimicrobiia	Acidimicrobiales	uncultured	bacterium_enrichment_culture_clone_auto73_4W	unclassified	-0.638	0.098	-0.198	0.738	0	0	0	1	0.74 0.006
**Alps-S**	OTUp_905	Bacteria	Chloroflexi	Anaerolineae	Anaerolineales	Anaerolineaceae	uncultured	uncultured_Gemmatimonadetes_bacterium	-0.276	-0.087	-0.272	0.635	0	0	0	1	0.63 0.045
**Alps-S**	OTUp_944	Bacteria	Planctomycetes	Pla4_lineage	uncultured_bacterium	unclassified	unclassified	unclassified	-0.652	0.336	0.208	0.109	0	1	1	1	0.65 0.041
**Alps-S**	OTUp_994	Bacteria	Planctomycetes	Planctomycetacia	Planctomycetales	Planctomycetaceae	uncultured	uncultured_bacterium	-0.440	-0.283	0.112	0.611	0	0	1	1	0.63 0.035
**Alps-S**	OTUp_1005	Bacteria	Acidobacteria	Acidobacteria	Subgroup_6	unclassified	unclassified	unclassified	-0.724	0.394	0.184	0.147	0	1	1	1	0.72 0.015
**Alps-S**	OTUp_1050	Bacteria	Planctomycetes	Planctomycetacia	Planctomycetales	Planctomycetaceae	uncultured	unclassified	-0.618	0.383	-0.153	0.388	0	1	0	1	0.67 0.036
**Alps-S**	OTUp_1054	Bacteria	Planctomycetes	Planctomycetacia	Planctomycetales	Planctomycetaceae	uncultured	uncultured_bacterium	-0.508	-0.450	0.210	0.748	0	0	1	1	0.83 0.000
**Alps-S**	OTUp_1093	Bacteria	Cyanobacteria	Chloroplast	unclassified	unclassified	unclassified	unclassified	-0.394	0.264	0.524	-0.394	0	1	1	0	0.68 0.049
**Alps-S**	OTUp_1103	Bacteria	Bacteroidetes	Sphingobacteriia	Sphingobacteriales	Chitinophagaceae	uncultured	uncultured_Chitinophagaceae_bacterium	-0.378	-0.339	-0.030	0.747	0	0	0	1	0.75 0.005
**Alps-S**	OTUp_1122	Bacteria	WD272	uncultured_bacterium	unclassified	unclassified	unclassified	unclassified	-0.345	-0.322	-0.111	0.778	0	0	0	1	0.78 0.005
**Alps-S**	OTUp_1127	Bacteria	Planctomycetes	Planctomycetacia	Planctomycetales	Planctomycetaceae	Gemmata	uncultured_bacterium	-0.414	0.348	-0.378	0.444	0	1	0	1	0.69 0.027
**Alps-S**	OTUp_1129	Bacteria	Verrucomicrobia	Spartobacteria	Chthoniobacterales	DA101_soil_group	uncultured_Prosthecobacter_sp.	unclassified	-0.358	0.059	-0.358	0.658	0	0	0	1	0.66 0.035
**Alps-S**	OTUp_1188	Bacteria	Acidobacteria	Acidobacteria	Subgroup_4	RB41	uncultured_bacterium	unclassified	-0.338	-0.230	-0.269	0.837	0	0	0	1	0.84 0.003
**Alps-S**	OTUp_1234	Bacteria	Chloroflexi	JG37-AG-4	uncultured_bacterium	unclassified	unclassified	unclassified	-0.323	0.319	-0.476	0.480	0	1	0	1	0.69 0.010
**Alps-S**	OTUp_1236	Bacteria	Chloroflexi	Anaerolineae	Anaerolineales	Anaerolineaceae	uncultured	uncultured_sludge_bacterium_S14	-0.323	-0.194	-0.220	0.738	0	0	0	1	0.74 0.007
**Alps-S**	OTUp_1261	Bacteria	Acidobacteria	Acidobacteria	Subgroup_3	Unknown_Family	Bryobacter	uncultured_bacterium	-0.433	-0.323	0.001	0.754	0	0	0	1	0.75 0.011
**Alps-S**	OTUp_1274	Bacteria	Chloroflexi	uncultured	uncultured_Caldilineaceae_bacterium	unclassified	unclassified	unclassified	-0.361	-0.361	-0.203	0.925	0	0	0	1	0.92 0.003
**Alps-S**	OTUp_1290	Bacteria	Bacteroidetes	Sphingobacteriia	Sphingobacteriales	env.OPS_17	unclassified	unclassified	-0.480	0.568	-0.323	0.235	0	1	0	1	0.70 0.026
**Alps-S**	OTUp_1348	Bacteria	Proteobacteria	Betaproteobacteria	Nitrosomonadales	Nitrosomonadaceae	uncultured	uncultured_Oxalobacteraceae_bacterium	-0.728	0.211	0.043	0.473	0	1	1	1	0.73 0.012
**Alps-S**	OTUp_1366	Bacteria	Planctomycetes	Planctomycetacia	Planctomycetales	Planctomycetaceae	uncultured	uncultured_bacterium	-0.202	-0.207	-0.325	0.734	0	0	0	1	0.73 0.018
**Alps-S**	OTUp_1428	Bacteria	Planctomycetes	Planctomycetacia	Planctomycetales	Planctomycetaceae	Gemmata	unclassified	-0.656	0.077	0.340	0.238	0	1	1	1	0.66 0.037

**Alps-S**	OTUp_1438	Bacteria	Planctomycetes	Planctomycetacia	Planctomycetales	Planctomycetaceae	Gemmata	uncultured_bacterium	-0.441	-0.227	0.009	0.659	0	0	0	1	0.66	0.042
**Alps-S**	OTUp_1473	Bacteria	Proteobacteria	Betaproteobacteria	Neisseriales	Neisseriaceae	Vogesella	uncultured_beta_proteobacterium	-0.352	-0.047	0.678	-0.278	0	0	1	0	0.68	0.029
**Alps-S**	OTUp_1536	Bacteria	Chlamydiae	Chlamydiae	Chlamydiales	Parachlamydiaceae	Candidatus_Metachlamydia	Candidatus_Metachlamydia_lacustris	-0.308	-0.117	-0.308	0.733	0	0	0	1	0.73	0.029
**Alps-S**	OTUp_1598	Bacteria	Parcubacteria	Parcubacteria_bacterium_SCGC_AAA011-A08	unclassified	unclassified	unclassified	unclassified	-0.140	-0.201	-0.339	0.680	0	0	0	1	0.68	0.037
**Alps-S**	OTUp_1602	Bacteria	Verrucomicrobia	Spartobacteria	Chthoniobacterales	DA101_soil_group	uncultured_bacterium	unclassified	-0.452	-0.123	-0.181	0.756	0	0	0	1	0.76	0.007
**Alps-S**	OTUp_1612	Bacteria	Chlorobi	Chlorobia	Chlorobiales	SJA-28	uncultured_bacterium	unclassified	-0.413	-0.374	0.386	0.402	0	0	1	1	0.68	0.032
**Alps-S**	OTUp_1617	Bacteria	Chloroflexi	JG30-KF-CM66	uncultured_soil_bacterium	unclassified	unclassified	unclassified	-0.446	0.608	-0.304	0.143	0	1	0	1	0.65	0.035
**Alps-S**	OTUp_1650	Bacteria	Verrucomicrobia	OPB35_soil_group	uncultured_bacterium	unclassified	unclassified	unclassified	-0.442	-0.033	-0.229	0.704	0	0	0	1	0.70	0.016
**Alps-S**	OTUp_1668	Bacteria	Proteobacteria	Gammaproteobacteria	Xanthomonadales	Xanthomonadaceae	unclassified	unclassified	-0.516	-0.218	0.046	0.688	0	0	0	1	0.69	0.040
**Alps-S**	OTUp_1683	Bacteria	Chloroflexi	JG37-AG-4	uncultured_soil_bacterium	unclassified	unclassified	unclassified	-0.187	-0.081	-0.390	0.658	0	0	0	1	0.66	0.041
**Alps-S**	OTUp_1700	Bacteria	Actinobacteria	Thermoleophilia	Gaiellales	uncultured	uncultured_Rubrobacterales_bacterium	unclassified	-0.668	0.651	0.097	-0.079	0	1	1	1	0.67	0.034
**Alps-S**	OTUp_1724	Bacteria	Planctomycetes	Planctomycetacia	Planctomycetales	Planctomycetaceae	Singulisphaera	uncultured_bacterium	-0.409	0.401	0.545	-0.537	0	1	1	0	0.82	0.002
**Alps-S**	OTUp_1759	Bacteria	Planctomycetes	Planctomycetacia	Planctomycetales	Planctomycetaceae	uncultured	uncultured_bacterium	-0.716	0.313	-0.009	0.412	0	1	1	1	0.72	0.019
**Alps-S**	OTUp_1768	Bacteria	Actinobacteria	Thermoleophilia	Solirubrobacterales	0319-6M6	uncultured_actinobacterium	unclassified	-0.251	-0.070	0.644	-0.324	0	0	1	0	0.64	0.043
**Alps-S**	OTUp_1771	Bacteria	Chloroflexi	uncultured_Bellilinea_sp.	unclassified	unclassified	unclassified	unclassified	-0.363	0.554	0.230	-0.421	0	1	1	0	0.68	0.022
**Alps-S**	OTUp_1784	Bacteria	Firmicutes	Bacilli	Bacillales	Planococcaceae	Psychrobacillus	Psychrobacillus_psychrodurans	-0.378	0.570	0.165	-0.356	0	1	1	0	0.64	0.047
**Alps-S**	OTUp_1813	Bacteria	Bacteroidetes	Sphingobacteriia	Sphingobacteriales	env.OPS_17	uncultured_bacterium	unclassified	-0.414	0.492	-0.421	0.343	0	1	0	1	0.72	0.013
**Alps-S**	OTUp_1820	Bacteria	Bacteroidetes	Sphingobacteriia	Sphingobacteriales	Chitinophagaceae	Ferruginibacter	unclassified	-0.520	-0.201	0.059	0.662	0	0	0	1	0.66	0.035
**Alps-S**	OTUp_1881	Bacteria	Chloroflexi	Anaerolineae	Anaerolineales	Anaerolineaceae	uncultured	uncultured_Gemmatimonadetes_bacterium	-0.263	-0.079	-0.408	0.750	0	0	0	1	0.75	0.009
**Alps-S**	OTUp_1931	Bacteria	Planctomycetes	Planctomycetacia	Planctomycetales	Planctomycetaceae	uncultured	uncultured_bacterium	-0.254	0.238	0.599	-0.582	0	1	1	0	0.72	0.012
**Alps-S**	OTUp_1999	Bacteria	Proteobacteria	Alphaproteobacteria	Rhodospirillales	DA111	uncultured_alpha_proteobacterium	unclassified	-0.253	-0.275	-0.168	0.696	0	0	0	1	0.70	0.036
**Alps-S**	OTUp_2009	Bacteria	Proteobacteria	Alphaproteobacteria	Rhodospirillales	Acetobacteraceae	uncultured	uncultured_bacterium	-0.429	-0.127	0.655	-0.099	0	0	1	0	0.66	0.041
**Alps-S**	OTUp_2015	Bacteria	Planctomycetes	Planctomycetacia	Planctomycetales	Planctomycetaceae	Planctomyces	unclassified	-0.575	-0.314	0.286	0.602	0	0	1	1	0.77	0.014
**Alps-S**	OTUp_2050	Bacteria	Gemmatimonadetes	Gemmatimonadetes	S0134_terrestrial_group	unclassified	unclassified	unclassified	-0.331	0.649	-0.470	0.151	0	1	0	1	0.69	0.013
**Alps-S**	OTUp_2070	Bacteria	Chloroflexi	Ktedonobacteria	B12-WMSP1	uncultured_Chloroflexi_bacterium	unclassified	unclassified	-0.253	0.555	0.309	-0.610	0	1	1	0	0.75	0.007
**Alps-S**	OTUp_2086	Bacteria	Planctomycetes	Phycisphaerae	WD2101_soil_group	uncultured_bacterium	unclassified	unclassified	-0.356	-0.360	-0.002	0.718	0	0	0	1	0.72	0.021
**Alps-S**	OTUp_2088	Bacteria	Chlorobi	Chlorobia	Chlorobiales	OPB56	uncultured_bacterium	unclassified	-0.422	0.300	-0.422	0.543	0	1	0	1	0.73	0.035
**Alps-S**	OTUp_2145	Bacteria	Planctomycetes	vadinHA49	unclassified	unclassified	unclassified	unclassified	-0.375	0.211	-0.385	0.548	0	1	0	1	0.66	0.044
**Alps-S**	OTUp_2171	Bacteria	Gemmatimonadetes	Gemmatimonadetes	Gemmatimonadales	Gemmatimonadaceae	Gemmatimonas	unclassified	-0.530	-0.313	0.197	0.645	0	0	1	1	0.73	0.011
**Alps-S**	OTUp_2177	Bacteria	Chloroflexi	unclassified	unclassified	unclassified	unclassified	unclassified	-0.281	-0.308	-0.173	0.761	0	0	0	1	0.76	0.007
**Alps-S**	OTUp_2204	Bacteria	Acidobacteria	Acidobacteria	Subgroup_6	unclassified	unclassified	unclassified	-0.682	0.029	0.170	0.483	0	1	1	1	0.68	0.029
**Alps-S**	OTUp_2215	Bacteria	Chlorobi	Chlorobia	Chlorobiales	OPB56	uncultured_bacterium	unclassified	-0.467	-0.109	-0.150	0.726	0	0	0	1	0.73	0.011
**Alps-S**	OTUp_2234	Bacteria	Acidobacteria	Acidobacteria	Subgroup_4	Unknown_Family	Blastocatella	uncultured_Acidobacteria_bacterium	-0.231	-0.531	0.031	0.731	0	0	0	1	0.73	0.012
**Alps-S**	OTUp_2255	Bacteria	Verrucomicrobia	Verrucomicrobia_Incertae_Sedis	Unknown_Order	Unknown_Family	Candidatus_Methylacidiphilum	uncultured_bacterium	-0.345	0.374	-0.515	0.486	0	1	0	1	0.74	0.010
**Alps-S**	OTUp_2315	Bacteria	Chloroflexi	JG30-KF-CM66	uncultured_Caldilinea_sp.	unclassified	unclassified	unclassified	-0.474	0.077	-0.325	0.722	0	0	0	1	0.72	0.009
**Alps-S**	OTUp_2326	Bacteria	Parcubacteria	uncultured_bacterium	unclassified	unclassified	unclassified	unclassified	-0.230	0.515	-0.597	0.312	0	1	0	1	0.72	0.018
**Alps-S**	OTUp_2336	Bacteria	Chloroflexi	Chloroflexia	Chloroflexales	Roseiflexaceae	Roseiflexus	uncultured_bacterium	-0.295	-0.154	-0.257	0.706	0	0	0	1	0.71	0.017
**Alps-S**	OTUp_2353	Bacteria	SM2F11	uncultured_bacterium	unclassified	unclassified	unclassified	unclassified	-0.259	-0.259	-0.259	0.777	0	0	0	1	0.78	0.031
**Alps-S**	OTUp_2374	Bacteria	Cyanobacteria	Melainabacteria	Obscuribacterales	uncultured_bacterium	unclassified	unclassified	-0.392	-0.060	-0.267	0.719	0	0	0	1	0.72	0.015
**Alps-S**	OTUp_2424	Bacteria	SHA-109	uncultured_bacterium	unclassified	unclassified	unclassified	unclassified	-0.401	-0.314	0.717	-0.002	0	0	1	0	0.72	0.011
**Alps-S**	OTUp_2428	Bacteria	Proteobacteria	Alphaproteobacteria	Caulobacterales	Caulobacteraceae	uncultured	uncultured_Rhizobiales_bacterium	0.032	-0.276	-0.449	0.694	0	0	0	1	0.69	0.022
**Alps-S**	OTUp_2462	Bacteria	Planctomycetes	Planctomycetacia	Planctomycetales	Planctomycetaceae	uncultured	uncultured_bacterium	-0.351	-0.169	-0.168	0.688	0	0	0	1	0.69	0.032
**Alps-S**	OTUp_2501	Bacteria	Verrucomicrobia	Spartobacteria	Chthoniobacterales	01D2Z36	uncultured_cyanobacterium	unclassified	-0.353	-0.234	-0.190	0.777	0	0	0	1	0.78	0.010
**Alps-S**	OTUp_2512	Bacteria	Proteobacteria	Deltaproteobacteria	Myxococcales	Sandaracinaceae	uncultured	uncultured_bacterium	-0.268	-0.276	-0.169	0.713	0	0	0	1	0.71	0.010
**Alps-S**	OTUp_2541	Bacteria	Parcubacteria	uncultured_bacterium	unclassified	unclassified	unclassified	unclassified	-0.179	-0.190	-0.415	0.784	0	0	0	1	0.78	0.007
**Alps-S**	OTUp_2565	Bacteria	Bacteroidetes	Sphingobacteriia	Sphingobacteriales	Chitinophagaceae	Flavisolibacter	uncultured_bacterium	-0.359	0.003	-0.290	0.647	0	0	0	1	0.65	0.040
**Alps-S**	OTUp_2630	Bacteria	Proteobacteria	Deltaproteobacteria	Myxococcales	Polyangiaceae	Sorangium	uncultured_bacterium	-0.191	-0.322	-0.054	0.567	0	0	0	1	0.57	0.043
**Alps-S**	OTUp_2752	Bacteria	Chlorobi	Chlorobia	Chlorobiales	OPB56	uncultured_bacterium	unclassified	-0.400	0.426	-0.397	0.371	0	1	0	1	0.69	0.022
**Alps-S**	OTUp_2762	Bacteria	Actinobacteria	Actinobacteria	Corynebacteriales	Nocardiaceae	Rhodococcus	Rhodococcus_qingshengii	-0.110	-0.322	0.867	-0.435	0	0	1	0	0.87	0.002
**Alps-S**	OTUp_2802	Bacteria	WD272	unclassified	unclassified	unclassified	unclassified	unclassified	-0.471	0.460	0.376	-0.365	0	1	1	0	0.72	0.011
**Alps-S**	OTUp_2900	Bacteria	Planctomycetes	Phycisphaerae	WD2101_soil_group	unclassified	unclassified	unclassified	-0.345	-0.130	-0.332	0.807	0	0	0	1	0.81	0.006
**Alps-S**	OTUp_2908	Bacteria	Planctomycetes	Planctomycetacia	Planctomycetales	Planctomycetaceae	Isosphaera	uncultured_bacterium	-0.456	0.006	-0.260	0.710	0	0	0	1	0.71	0.022
**Alps-S**	OTUp_2915	Bacteria	Planctomycetes	Planctomycetacia	Planctomycetales	Planctomycetaceae	Singulisphaera	uncultured_bacterium	-0.501	0.621	0.117	-0.237	0	1	1	0	0.64	0.036
**Alps-S**	OTUp_2946	Bacteria	Proteobacteria	Deltaproteobacteria	Myxococcales	Polyangiaceae	Byssovorax	uncultured_bacterium	-0.439	-0.269	0.778	-0.069	0	0	1	0	0.78	0.009
**Alps-S**	OTUp_2948	Bacteria	Chloroflexi	Anaerolineae	Anaerolineales	Anaerolineaceae	uncultured	uncultured_Anaerolinea_sp.	-0.404	-0.185	-0.230	0.819	0	0	0	1	0.82	0.007
**Alps-S**	OTUp_2981	Bacteria	Chloroflexi	JG37-AG-4	uncultured_bacterium	unclassified	unclassified	unclassified	-0.578	0.667	0.182	-0.270	0	1	1	0	0.74	0.019
**Alps-S**	OTUp_3011	Bacteria	Planctomycetes	Planctomycetacia	Planctomycetales	Planctomycetaceae	Singulisphaera	uncultured_planctomycete	-0.290	-0.331	-0.197	0.817	0	0	0	1	0.82	0.005
**Alps-S**	OTUp_3028	Bacteria	Proteobacteria	Alphaproteobacteria	Rhodospirillales	Acetobacteraceae	unclassified	unclassified	-0.461	0.434	0.355	-0.328	0	1	1	0	0.68	0.025
**Alps-S**	OTUp_3068	Bacteria	Verrucomicrobia	OPB35_soil_group	uncultured_bacterium	unclassified	unclassified	unclassified	-0.240	0.562	-0.573	0.251	0	1	0	1	0.70	0.032
**Alps-S**	OTUp_3074	Bacteria	Planctomycetes	Planctomycetacia	Planctomycetales	Planctomycetaceae	Gemmata	uncultured_planctomycete	-0.375	-0.245	-0.122	0.743	0	0	0	1	0.74	0.029
**Alps-S**	OTUp_3125	Bacteria	Firmicutes	Bacilli	Bacillales	Paenibacillaceae	Paenibacillus	Paenibacillus_pectinilyticus	-0.326	-0.315	-0.019	0.659	0	0	0	1	0.66	0.036
**Alps-S**	OTUp_3166	Bacteria	Parcubacteria	uncultured_bacterium	unclassified	unclassified	unclassified	unclassified	0.080	-0.506	-0.245	0.671	0	0	0	1	0.67	0.027
**Alps-S**	OTUp_3174	Bacteria	Proteobacteria	Deltaproteobacteria	Myxococcales	P3OB-42	uncultured_bacterium	unclassified	-0.731	0.304	0.303	0.124	0	1	1	1	0.73	0.015
**Alps-S**	OTUp_3185	Bacteria	Acidobacteria	Acidobacteria	Subgroup_17	uncultured_Acidobacteria_bacterium	unclassified	unclassified	-0.260	0.479	0.363	-0.582	0	1	1	0	0.73	0.022
**Alps-S**	OTUp_3213	Bacteria	Proteobacteria	Alphaproteobacteria	Sphingomonadales	Sphingomonadaceae	Novosphingobium	uncultured_alpha_proteobacterium	-0.564	-0.278	0.367	0.475	0	0	1	1	0.73	0.010
**Alps-S**	OTUp_3247	Bacteria	Proteobacteria	Deltaproteobacteria	Myxococcales	Haliangiaceae	Haliangium	uncultured_bacterium	-0.295	-0.295	-0.145	0.735	0	0	0	1	0.73	0.031
**Alps-S**	OTUp_3258	Bacteria	Chloroflexi	Anaerolineae	Anaerolineales	Anaerolineaceae	uncultured	uncultured_Gemmatimonadetes_bacterium	-0.396	-0.310	0.009	0.698	0	0	0	1	0.70	0.039
**Alps-S**	OTUp_3261	Bacteria	Planctomycetes	Planctomycetacia	Planctomycetales	Planctomycetaceae	uncultured	uncultured_bacterium	-0.381	-0.381	0.857	-0.096	0	0	1	0	0.86	0.008
**Alps-S**	OTUp_3266	Bacteria	Actinobacteria	Thermoleophilia	Gaiellales	uncultured	unclassified	unclassified	-0.753	0.262	0.435	0.057	0	1	1	1	0.75	0.003
**Alps-S**	OTUp_3288	Bacteria	Verrucomicrobia	OPB35_soil_group	unclassified	unclassified	unclassified	unclassified	-0.260	-0.208	-0.172	0.641	0	0	0	1	0.64	0.029
**Alps-S**	OTUp_3322	Bacteria	Chloroflexi	Ktedonobacteria	C0119	uncultured_bacterium	unclassified	unclassified	-0.412	-0.272	0.849	-0.165	0	0	1	0	0.85	0.007
**Alps-S**	OTUp_3335	Bacteria	Planctomycetes	Planctomycetacia	Planctomycetales	Planctomycetaceae	uncultured	uncultured_bacterium	-0.343	-0.343	0.002	0.685	0	0	0	1	0.68	0.036
**Alps-S**	OTUp_3379	Bacteria	Actinobacteria	Thermoleophilia	Solirubrobacterales	Patulibacteraceae	Patulibacter	uncultured_endolithic_bacterium	-0.332	-0.332	-0.039	0.702	0	0	0	1	0.70	0.031
**Alps-S**	OTUp_3443	Bacteria	Planctomycetes	Planctomycetacia	Planctomycetales	Planctomycetaceae	uncultured	unclassified	-0.168	-0.194	0.708	-0.346	0	0	1	0	0.71	0.031
**Alps-S**	OTUp_3575	Bacteria	Proteobacteria	Deltaproteobacteria	Myxococcales	uncultured	uncultured_bacterium	unclassified	-0.671	0.365	0.348	-0.042	0	1	1	1	0.67	0.041
**Alps-S**	OTUp_3626	Bacteria	Chloroflexi	Anaerolineae	Anaerolineales	Anaerolineaceae	uncultured	uncultured_Gemmatimonadetes_bacterium	-0.495	0.235	-0.378	0.638	0	1	0	1	0.76	0.009
**Alps-S**	OTUp_3630	Bacteria	Bacteroidetes	Cytophagia	Cytophagales	Cytophagaceae	Spirosoma	uncultured_bacterium	-0.257	-0.450	0.039	0.668	0	0	0	1	0.67	0.040
**Alps-S**	OTUp_3675	Bacteria	Bacteroidetes	Sphingobacteriia	Sphingobacteriales	Chitinophagaceae	Terrimonas	uncultured_Bacteroidetes_bacterium	-0.245	-0.245	-0.245	0.735	0	0	0	1	0.73	0.031
**Alps-S**	OTUp_3740	Bacteria	Proteobacteria	Alphaproteobacteria	Rhizobiales	Xanthobacteraceae	unclassified	unclassified	-0.425	0.593	0.191	-0.360	0	1	1	0	0.68	0.037
**Alps-S**	OTUp_3741	Bacteria	Planctomycetes	Phycisphaerae	CPla-3_termite_group	uncultured_bacterium	unclassified	unclassified	-0.136	-0.154	-0.340	0.631	0	0	0	1	0.63	0.031
**Alps-S**	OTUp_3752	Bacteria	unclassified	unclassified	unclassified	unclassified	unclassified	unclassified	-0.242	-0.225	0.671	-0.204	0	0	1	0	0.67	0.036
**Alps-S**	OTUp_3782	Bacteria	Planctomycetes	Phycisphaerae	Phycisphaerales	Phycisphaeraceae	SM1A02	uncultured_bacterium	-0.211	-0.211	-0.139	0.560	0	0	0	1	0.56	0.029
**Alps-S**	OTUp_3792	Bacteria	Armatimonadetes	unclassified	unclassified	unclassified	unclassified	unclassified	-0.249	-0.214	-0.414	0.878	0	0	0	1	0.88	0.003
**Alps-S**	OTUp_3863	Bacteria	Armatimonadetes	uncultured_bacterium	unclassified	unclassified	unclassified	unclassified	-0.277	-0.277	-0.277	0.831	0	0	0	1	0.83	0.028
**Alps-S**	OTUp_3907	Bacteria	Parcubacteria	uncultured_bacterium	unclassified	unclassified	unclassified	unclassified	-0.142	-0.286	-0.286	0.715	0	0	0	1	0.71	0.031
**Alps-S**	OTUp_3980	Bacteria	Verrucomicrobia	OPB35_soil_group	uncultured_bacterium	unclassified	unclassified	unclassified	-0.586	-0.268	0.443	0.411	0	0	1	1	0.74	0.007
**Alps-S**	OTUp_3988	Bacteria	Verrucomicrobia	Verrucomicrobia_Incertae_Sedis	Unknown_Order	Unknown_Family	Candidatus_Methylacidiphilum	uncultured_bacterium	-0.079	-0.311	-0.311	0.702	0	0	0	1	0.70	0.028
**Alps-S**	OTUp_4002	Bacteria	Firmicutes	Clostridia	Clostridiales	Ruminococcaceae	Candidatus_Soleaferrea	uncultured_bacterium	-0.163	-0.322	-0.183	0.668	0	0	0	1	0.67	0.025
**Alps-S**	OTUp_4259	Bacteria	Acidobacteria	Acidobacteria	Subgroup_6	uncultured_bacterium	unclassified	unclassified	-0.299	-0.299	-0.093	0.690	0	0	0	1	0.69	0.031
**Alps-S**	OTUp_4293	Bacteria	Verrucomicrobia	Spartobacteria	Chthoniobacterales	DA101_soil_group	uncultured_bacterium	unclassified	-0.319	-0.038	-0.282	0.638	0	0	0	1	0.64	0.041
**Alps-S**	OTUp_4415	Bacteria	Planctomycetes	Planctomycetacia	Planctomycetales	Planctomycetaceae	uncultured	unclassified	-0.352	0.501	-0.396	0.247	0	1	0	1	0.65	0.040
**Alps-S**	OTUp_4713	Bacteria	Cyanobacteria	Chloroplast	unclassified	unclassified	unclassified	unclassified	-0.241	-0.089	0.655	-0.325	0	0	1	0	0.66	0.038
**Alps-S**	OTUp_4790	Bacteria	Proteobacteria	Deltaproteobacteria	Myxococcales	Polyangiaceae	Sorangium	uncultured_bacterium	-0.233	-0.239	0.714	-0.242	0	0	1	0	0.71	0.035
**Alps-S**	OTUp_4887	Bacteria	Verrucomicrobia	Spartobacteria	Chthoniobacterales	DA101_soil_group	uncultured_soil_bacterium	unclassified	-0.125	-0.419	-0.255	0.800	0	0	0	1	0.80	0.005
**Alps-S**	OTUp_5025	Bacteria	Acidobacteria	Acidobacteria	Subgroup_3	Unknown_Family	Bryobacter	unclassified	-0.612	0.421	0.459	-0.268	0	1	1	0	0.76	0.005
**Alps-S**	OTUp_5159	Bacteria	Verrucomicrobia	Spartobacteria	Chthoniobacterales	DA101_soil_group	unclassified	unclassified	-0.606	0.105	-0.168	0.669	0	1	0	1	0.67	0.033

**Alps-S**	OTUp_5190	Bacteria	Proteobacteria	Deltaproteobacteria	Myxococcales	Polyangiaceae	Sorangium	uncultured_bacterium	-0.335	-0.206	-0.218	0.759	0	0	0	1	0.76	0.003
**Alps-S**	OTUp_5208	Bacteria	Proteobacteria	Betaproteobacteria	Nitrosomonadales	Nitrosomonadaceae	uncultured	uncultured_delta_proteobacterium	-0.654	0.072	0.076	0.505	0	1	1	1	0.65	0.040
**Alps-S**	OTUp_5490	Bacteria	Proteobacteria	Deltaproteobacteria	Myxococcales	Cystobacteraceae	Anaeromyxobacter	unclassified	-0.519	0.508	0.309	-0.298	0	1	1	0	0.71	0.018
**Alps-S**	OTUp_5525	Bacteria	WD272	uncultured_Firmicutes_bacterium	unclassified	unclassified	unclassified	unclassified	-0.283	-0.427	0.006	0.704	0	0	0	1	0.70	0.022
**Alps-S**	OTUp_5683	Bacteria	Chlorobi	Chlorobia	Chlorobiales	SJA-28	uncultured_bacterium	unclassified	-0.120	-0.067	-0.494	0.681	0	0	0	1	0.68	0.024
**Alps-S**	OTUp_5755	Bacteria	Proteobacteria	TA18	uncultured_Firmicutes_bacterium	unclassified	unclassified	unclassified	-0.492	-0.268	0.273	0.488	0	0	1	1	0.66	0.037
**Alps-S**	OTUp_6035	Bacteria	Verrucomicrobia	Spartobacteria	Chthoniobacterales	DA101_soil_group	uncultured_Prosthecobacter_sp.	unclassified	-0.436	-0.139	-0.133	0.708	0	0	0	1	0.71	0.020
**Alps-S**	OTUp_6455	Bacteria	Armatimonadetes	uncultured_Armatimonadetes_bacterium	unclassified	unclassified	unclassified	unclassified	-0.311	-0.356	-0.060	0.727	0	0	0	1	0.73	0.019
**Alps-S**	OTUp_6596	Bacteria	Proteobacteria	Deltaproteobacteria	Myxococcales	P3OB-42	uncultured_bacterium	unclassified	-0.366	0.028	0.652	-0.314	0	0	1	0	0.65	0.039
**Alps-S**	OTUp_6917	Bacteria	Chloroflexi	JG30-KF-CM66	uncultured_soil_bacterium	unclassified	unclassified	unclassified	-0.447	0.517	0.376	-0.447	0	1	1	0	0.77	0.013
**Alps-S**	OTUp_6938	Bacteria	Actinobacteria	Acidimicrobiia	Acidimicrobiales	uncultured	uncultured_bacterium	unclassified	-0.388	-0.060	-0.388	0.837	0	0	0	1	0.84	0.009
**Alps-S**	OTUp_7048	Bacteria	Proteobacteria	Deltaproteobacteria	Bdellovibrionales	Bdellovibrionaceae	Bdellovibrio	unclassified	-0.230	-0.230	0.689	-0.230	0	0	1	0	0.69	0.031
**Alps-S**	OTUp_7262	Bacteria	Verrucomicrobia	Opitutae	Opitutales	Opitutaceae	Opitutus	uncultured_bacterium	-0.170	-0.171	-0.396	0.737	0	0	0	1	0.74	0.009
**Alps-S**	OTUp_7409	Bacteria	Proteobacteria	Deltaproteobacteria	Bdellovibrionales	Bdellovibrionaceae	Bdellovibrio	uncultured_bacterium	-0.359	0.043	-0.359	0.676	0	0	0	1	0.68	0.032
**Alps-S**	OTUp_7509	Bacteria	Actinobacteria	Actinobacteria	Micrococcales	Micrococcaceae	Arthrobacter	Arthrobacter_alkaliphilus	-0.144	-0.418	0.955	-0.393	0	0	1	0	0.96	0.002
**Alps-S**	OTUp_7554	Bacteria	Planctomycetes	Planctomycetacia	Planctomycetales	Planctomycetaceae	uncultured	uncultured_bacterium	-0.568	0.405	0.511	-0.347	0	1	1	0	0.79	0.004
**Alps-S**	OTUp_7664	Bacteria	Planctomycetes	Planctomycetacia	Planctomycetales	Planctomycetaceae	unclassified	unclassified	-0.182	-0.051	0.656	-0.423	0	0	1	0	0.66	0.036
**Alps-S**	OTUp_8190	Bacteria	Verrucomicrobia	Spartobacteria	Chthoniobacterales	DA101_soil_group	unclassified	unclassified	-0.547	-0.178	-0.057	0.782	0	0	0	1	0.78	0.008
**Alps-S**	OTUp_8305	Bacteria	Acidobacteria	Acidobacteria	Acidobacteriales	Acidobacteriaceae	uncultured	unclassified	-0.328	-0.324	-0.009	0.662	0	0	0	1	0.66	0.023
**Alps-S**	OTUp_8472	Bacteria	SHA-109	uncultured_alpha_proteobacterium	unclassified	unclassified	unclassified	unclassified	-0.411	-0.411	0.015	0.806	0	0	0	1	0.81	0.003
**Alps-S**	OTUp_8572	Bacteria	Proteobacteria	Alphaproteobacteria	Rhodospirillales	Acetobacteraceae	uncultured	uncultured_proteobacterium	-0.282	-0.467	0.348	0.400	0	0	1	1	0.65	0.049
**Alps-S**	OTUp_9069	Bacteria	Proteobacteria	Gammaproteobacteria	Xanthomonadales	Xanthomonadaceae	unclassified	unclassified	-0.199	-0.058	-0.570	0.827	0	0	0	1	0.83	0.003
**Alps-S**	OTUp_9406	Bacteria	Gemmatimonadetes	Gemmatimonadetes	Gemmatimonadales	Gemmatimonadaceae	Gemmatimonas	unclassified	-0.252	-0.252	-0.252	0.755	0	0	0	1	0.75	0.031
**Alps-S**	OTUp_9877	Bacteria	Planctomycetes	Planctomycetacia	Planctomycetales	Planctomycetaceae	unclassified	unclassified	-0.277	-0.277	-0.277	0.831	0	0	0	1	0.83	0.028
**Alps-S**	OTUp_9988	Bacteria	Bacteroidetes	Sphingobacteriia	Sphingobacteriales	Chitinophagaceae	Segetibacter	uncultured_bacterium	0.056	-0.411	-0.411	0.766	0	0	0	1	0.77	0.012
**Alps-S**	OTUp_10124	Bacteria	Bacteroidetes	Sphingobacteriia	Sphingobacteriales	Chitinophagaceae	Ferruginibacter	uncultured_Bacteroidetes_bacterium	-0.441	-0.324	-0.058	0.824	0	0	0	1	0.82	0.003
**Alps-S**	OTUp_10412	Bacteria	Acidobacteria	Acidobacteria	Subgroup_3	Unknown_Family	Bryobacter	unclassified	-0.207	-0.104	-0.362	0.673	0	0	0	1	0.67	0.032
**Alps-S**	OTUp_10594	Bacteria	Proteobacteria	Alphaproteobacteria	Rhodospirillales	Acetobacteraceae	Acidiphilium	unclassified	-0.315	0.395	0.435	-0.515	0	1	1	0	0.72	0.015
**Alps-S**	OTUp_11054	Bacteria	Planctomycetes	Planctomycetacia	Planctomycetales	Planctomycetaceae	Gemmata	uncultured_Gemmata_sp.	-0.176	-0.279	-0.289	0.743	0	0	0	1	0.74	0.007

1: significantly associated ($p < 0.05$), 0: non-significantly associated.
Arctic: Arctic soils, Alps: alpine soils, -N: north-exposed, -S: south-exposed
D-FTC: daily freeze-thaw cycles, W-FTC: weekly freeze-thaw cycles, ctrl +5°C: controls +5°C, ctrl -5°C: controls -5°C
