# Supplementary material for: Functional and Structural Responses of Arctic and Alpine Soil Prokaryotic and Fungal Communities Under Freeze-Thaw Cycles of Different Frequencies
Source: Front Microbiol. 2020 May 25;11:982. doi: 10.3389/fmicb.2020.00982 (PMC7261861; doi:10.3389/fmicb.2020.00982)
Supplement: Supplementary file 8 [file Data_Sheet_2.zip › Supplementary Table 9.PDF]

**Soil**	**Otus**	**Kingdom**	**Phylum**	**Class**	**Order**	**Family**	**Genus**	**Species**	**ctrl +5°C**	**ctrl -5°C**	**D-FTC**	**W-FTC**	**s.ctrl +5°C**	**s.ctrl -5°C**	**s.D-FTC**	**s.W-FTC**	**stat**	***p***
**Arctic-N**	OTUe_24	Fungi	Basidiomycota	Agaricomycetes	Thelephorales	Thelephoraceae	Tomentella	Tomentella_sp_29B	-0.203	-0.127	-0.382	0.712	0	0	0	1	0.71	0.017
**Arctic-N**	OTUe_186	Fungi	unclassified_Fungi	unclassified_Fungi	unclassified_Fungi	unclassified_Fungi	unclassified_Fungi	fungal_sp_ANTELF7	-0.266	-0.183	-0.286	0.734	0	0	0	1	0.73	0.001
**Arctic-N**	OTUe_289	Fungi	Basidiomycota	Microbotryomycetes	Leucosporidiales	Leucosporidiaceae	Leucosporidium	Leucosporidium_drummii	-0.114	-0.307	0.677	-0.256	0	0	1	0	0.68	0.027
**Arctic-N**	OTUe_354	Fungi	Ascomycota	unclassified	unclassified	unclassified	unclassified	unclassified	-0.332	-0.443	-0.043	0.818	0	0	0	1	0.82	0.007
**Arctic-N**	OTUe_367	Fungi	Ascomycota	unclassified	unclassified	unclassified	unclassified	unclassified	-0.163	-0.152	-0.177	0.492	0	0	0	1	0.49	0.045
**Arctic-N**	OTUe_517	Fungi	Ascomycota	unclassified	unclassified	unclassified	unclassified	unclassified	-0.096	-0.269	-0.268	0.633	0	0	0	1	0.63	0.036
**Arctic-N**	OTUe_541	Fungi	Basidiomycota	Agaricomycetes	Thelephorales	Thelephoraceae	unclassified	unclassified	-0.330	-0.335	-0.070	0.735	0	0	0	1	0.74	0.008
**Arctic-N**	OTUe_567	Fungi	Ascomycota	unclassified	unclassified	unclassified	unclassified	unclassified	-0.191	-0.187	-0.142	0.520	0	0	0	1	0.52	0.033
**Arctic-N**	OTUe_808	Fungi	Basidiomycota	Agaricomycetes	Cantharellales	Clavulinaceae	Clavulina	Clavulina_rugosa	-0.015	-0.343	-0.298	0.656	0	0	0	1	0.66	0.047
**Arctic-N**	OTUe_1061	Fungi	Ascomycota	Leotiomycetes	Helotiales	unclassified_Helotiales	unclassified_Helotiales	Helotiales_sp_SB53	-0.139	-0.162	0.709	-0.407	0	0	1	0	0.71	0.015
**Arctic-N**	OTUe_1064	Fungi	unclassified	unclassified	unclassified	unclassified	unclassified	unclassified	-0.235	-0.193	-0.188	0.615	0	0	0	1	0.62	0.032
**Arctic-N**	OTUe_1113	Fungi	unclassified_Fungi	unclassified_Fungi	unclassified_Fungi	unclassified_Fungi	unclassified_Fungi	fungal_endophyte_sp_AP770	0.095	-0.369	0.622	-0.348	0	0	1	0	0.62	0.045
**Arctic-N**	OTUe_1116	Fungi	Ascomycota	unclassified	unclassified	unclassified	unclassified	unclassified	-0.399	-0.009	0.624	-0.216	0	0	1	0	0.62	0.038
**Arctic-N**	OTUe_1129	Fungi	Basidiomycota	unclassified_Basidiomycota	unclassified_Basidiomycota	unclassified_Basidiomycota	unclassified_Basidiomycota	Basidiomycota_sp_CC_15_08	-0.133	-0.425	0.676	-0.118	0	0	1	0	0.68	0.033
**Arctic-N**	OTUe_1148	Fungi	Ascomycota	unclassified	unclassified	unclassified	unclassified	unclassified	-0.185	-0.214	-0.214	0.613	0	0	0	1	0.61	0.001
**Arctic-N**	OTUe_3640	Fungi	Ascomycota	Eurotiomycetes	Chaetothyriales	Herpotrichiellaceae	Cladophialophora	Cladophialophora_sp_Leo1	-0.207	-0.207	0.621	-0.207	0	0	1	0	0.62	0.031
**Arctic-N**	OTUe_3706	Fungi	Ascomycota	Sordariomycetes	unclassified	unclassified	unclassified	unclassified	-0.515	0.662	0.115	-0.262	0	1	1	0	0.67	0.032
**Arctic-N**	OTUe_3727	Fungi	Ascomycota	Eurotiomycetes	Eurotiales	Trichocomaceae	Penicillium	Penicillium_janczewskii	-0.195	0.596	0.160	-0.560	0	1	1	0	0.65	0.038
**Arctic-S**	OTUe_2	Fungi	Ascomycota	Leotiomycetes	Leotiomycetes_order_Incertae_sedis	Leotiomycetes_family_Incertae_sedis	Geomyces	unclassified	-0.338	-0.406	0.575	0.169	0	0	1	1	0.64	0.048
**Arctic-S**	OTUe_5	Fungi	unclassified	unclassified	unclassified	unclassified	unclassified	unclassified	-0.354	-0.415	0.822	-0.053	0	0	1	0	0.82	0.006
**Arctic-S**	OTUe_32	Fungi	Basidiomycota	Agaricomycetes	Agaricales	Hygrophoraceae	Hygrocybe	Hygrocybe_cf_conica_DJ_Lodge_DJL05TN89_	-0.375	-0.424	0.116	0.684	0	0	1	1	0.69	0.033
**Arctic-S**	OTUe_42	Fungi	Ascomycota	Dothideomycetes	Pleosporales	unclassified	unclassified	unclassified	-0.226	-0.252	-0.205	0.682	0	0	0	1	0.68	0.024
**Arctic-S**	OTUe_56	Fungi	unclassified_Fungi	unclassified_Fungi	unclassified_Fungi	unclassified_Fungi	unclassified_Fungi	uncultured_fungus	-0.319	-0.457	0.304	0.472	0	0	1	1	0.67	0.023
**Arctic-S**	OTUe_70	Fungi	Basidiomycota	Tremellomycetes	Cystofilobasidiales	Cystofilobasidiaceae	Mrakia	Mrakia_sp_ABS_2	-0.220	-0.393	0.743	-0.129	0	0	1	0	0.74	0.008
**Arctic-S**	OTUe_76	Fungi	Ascomycota	Dothideomycetes	Capnodiales	Davidiellaceae	Cladosporium	Cladosporium_nigrellum	-0.097	-0.288	0.659	-0.274	0	0	1	0	0.66	0.046
**Arctic-S**	OTUe_90	Fungi	Ascomycota	unclassified	unclassified	unclassified	unclassified	unclassified	-0.385	-0.505	0.436	0.454	0	0	1	1	0.77	0.002
**Arctic-S**	OTUe_113	Fungi	Ascomycota	unclassified	unclassified	unclassified	unclassified	unclassified	-0.464	-0.467	0.538	0.393	0	0	1	1	0.81	0.001
**Arctic-S**	OTUe_120	Fungi	Ascomycota	unclassified	unclassified	unclassified	unclassified	unclassified	-0.392	-0.531	0.293	0.630	0	0	1	1	0.80	0.005
**Arctic-S**	OTUe_154	Fungi	Ascomycota	Leotiomycetes	Helotiales	Helotiales_family_Incertae_sedis	Cadophora	Cadophora_sp_29d	-0.364	-0.448	0.417	0.395	0	0	1	1	0.70	0.008
**Arctic-S**	OTUe_161	Fungi	Ascomycota	unclassified	unclassified	unclassified	unclassified	unclassified	-0.344	-0.530	0.419	0.455	0	0	1	1	0.76	0.010
**Arctic-S**	OTUe_194	Fungi	unclassified	unclassified	unclassified	unclassified	unclassified	unclassified	-0.424	-0.485	0.545	0.363	0	0	1	1	0.79	0.001
**Arctic-S**	OTUe_198	Fungi	Ascomycota	Dothideomycetes	unclassified	unclassified	unclassified	unclassified	-0.172	-0.388	0.797	-0.237	0	0	1	0	0.80	0.007
**Arctic-S**	OTUe_212	Fungi	Basidiomycota	Agaricomycetes	Agaricales	Inocybaceae	Inocybe	Inocybe_squarrosa	-0.320	-0.294	-0.107	0.720	0	0	0	1	0.72	0.025
**Arctic-S**	OTUe_226	Fungi	Ascomycota	Leotiomycetes	Helotiales	unclassified	unclassified	unclassified	-0.466	-0.522	0.599	0.389	0	0	1	1	0.86	0.001
**Arctic-S**	OTUe_245	Fungi	unclassified	unclassified	unclassified	unclassified	unclassified	unclassified	-0.422	-0.483	0.434	0.471	0	0	1	1	0.78	0.007
**Arctic-S**	OTUe_258	Fungi	Ascomycota	Ascomycota_class_Incertae_sedis	Ascomycota_order_Incertae_sedis	Ascomycota_family_Incertae_sedis	Furcaspora	Furcaspora_eucalypti	-0.389	-0.427	0.245	0.571	0	0	1	1	0.71	0.017
**Arctic-S**	OTUe_265	Fungi	Ascomycota	Leotiomycetes	Helotiales	unclassified_Helotiales	unclassified_Helotiales	Helotiales_sp_SB53	-0.359	-0.184	-0.041	0.584	0	0	0	1	0.58	0.050
**Arctic-S**	OTUe_281	Fungi	Ascomycota	unclassified	unclassified	unclassified	unclassified	unclassified	-0.355	-0.466	0.563	0.259	0	0	1	1	0.71	0.010
**Arctic-S**	OTUe_282	Fungi	unclassified	unclassified	unclassified	unclassified	unclassified	unclassified	-0.377	-0.493	0.233	0.637	0	0	1	1	0.75	0.008
**Arctic-S**	OTUe_296	Fungi	Ascomycota	unclassified	unclassified	unclassified	unclassified	unclassified	-0.283	-0.432	0.008	0.707	0	0	0	1	0.71	0.002
**Arctic-S**	OTUe_323	Fungi	unclassified	unclassified	unclassified	unclassified	unclassified	unclassified	-0.434	-0.299	0.447	0.285	0	0	1	1	0.63	0.029
**Arctic-S**	OTUe_342	Fungi	Basidiomycota	Agaricomycetes	Agaricales	Clavariaceae	Clavaria	Clavaria_sp	-0.337	-0.329	0.002	0.664	0	0	0	1	0.66	0.011
**Arctic-S**	OTUe_351	Fungi	Ascomycota	Leotiomycetes	Helotiales	unclassified_Helotiales	unclassified_Helotiales	Helotiales_sp_SB53	-0.325	-0.442	0.266	0.501	0	0	1	1	0.66	0.042
**Arctic-S**	OTUe_363	Fungi	Ascomycota	unclassified	unclassified	unclassified	unclassified	unclassified	-0.291	-0.541	0.427	0.405	0	0	1	1	0.72	0.020
**Arctic-S**	OTUe_364	Fungi	Ascomycota	unclassified	unclassified	unclassified	unclassified	unclassified	-0.145	-0.336	0.775	-0.294	0	0	1	0	0.77	0.006
**Arctic-S**	OTUe_400	Fungi	Ascomycota	Leotiomycetes	Helotiales	unclassified	unclassified	unclassified	-0.342	-0.425	0.183	0.584	0	0	1	1	0.66	0.035
**Arctic-S**	OTUe_451	Fungi	Ascomycota	unclassified	unclassified	unclassified	unclassified	unclassified	-0.320	-0.555	0.290	0.585	0	0	1	1	0.76	0.008
**Arctic-S**	OTUe_473	Fungi	Basidiomycota	Agaricomycetes	Sebacinales	Sebacinaceae	Sebacina	salal_root_associated_fungus_UBCTRA_154210	-0.211	-0.643	0.368	0.485	0	0	1	1	0.74	0.003
**Arctic-S**	OTUe_476	Fungi	Ascomycota	Leotiomycetes	unclassified	unclassified	unclassified	unclassified	-0.296	-0.432	0.790	-0.062	0	0	1	0	0.79	0.006
**Arctic-S**	OTUe_486	Fungi	Ascomycota	Leotiomycetes	Helotiales	unclassified	unclassified	unclassified	-0.053	-0.161	0.719	-0.506	0	0	1	0	0.72	0.012
**Arctic-S**	OTUe_559	Fungi	Ascomycota	Leotiomycetes	unclassified	unclassified	unclassified	unclassified	-0.193	-0.477	0.685	-0.015	0	0	1	0	0.68	0.029
**Arctic-S**	OTUe_581	Fungi	Ascomycota	Dothideomycetes	Capnodiales	unclassified	unclassified	unclassified	-0.210	-0.239	0.559	-0.110	0	0	1	0	0.56	0.034
**Arctic-S**	OTUe_609	Fungi	unclassified	unclassified	unclassified	unclassified	unclassified	unclassified	-0.246	-0.321	-0.101	0.668	0	0	0	1	0.67	0.009
**Arctic-S**	OTUe_716	Fungi	Ascomycota	Sordariomycetes	Hypocreales	Hypocreales_family_Incertae_sedis	Stilbella	Stilbella_sp_RM5_6	-0.296	-0.514	0.356	0.454	0	0	1	1	0.70	0.020
**Arctic-S**	OTUe_854	Fungi	Ascomycota	Leotiomycetes	Helotiales	unclassified_Helotiales	unclassified_Helotiales	unclassified	-0.453	-0.499	0.250	0.703	0	0	1	1	0.82	0.000
**Arctic-S**	OTUe_910	Fungi	Basidiomycota	Agaricomycetes	Agaricales	Clavariaceae	Ramariopsis	unclassified	-0.307	-0.203	0.770	-0.259	0	0	1	0	0.77	0.010
**Arctic-S**	OTUe_939	Fungi	Basidiomycota	Agaricomycetes	Agaricales	Clavariaceae	Clavaria	Clavaria_acuta	-0.304	-0.468	0.193	0.579	0	0	1	1	0.67	0.038
**Arctic-S**	OTUe_986	Fungi	Ascomycota	Dothideomycetes	unclassified	unclassified	unclassified	unclassified	-0.248	-0.248	0.743	-0.248	0	0	1	0	0.74	0.029
**Arctic-S**	OTUe_1077	Fungi	Ascomycota	Eurotiomycetes	Chaetothyriales	Herpotrichiellaceae	Capronia	Capronia_sp_96003a	-0.268	-0.238	0.807	-0.301	0	0	1	0	0.81	0.005
**Arctic-S**	OTUe_1130	Fungi	Ascomycota	unclassified	unclassified	unclassified	unclassified	unclassified	-0.323	-0.164	-0.227	0.713	0	0	0	1	0.71	0.032
**Arctic-S**	OTUe_1187	Fungi	Basidiomycota	Agaricomycetes	Agaricales	Clavariaceae	Ramariopsis	Ramariopsis_laeticolor	-0.412	-0.293	-0.076	0.780	0	0	0	1	0.78	0.004
**Arctic-S**	OTUe_1189	Fungi	Ascomycota	Dothideomycetes	Capnodiales	Teratosphaeriaceae	Devriesia	Devriesia_sp	-0.272	0.004	0.690	-0.422	0	0	1	0	0.69	0.042
**Arctic-S**	OTUe_1306	Fungi	Ascomycota	Leotiomycetes	Helotiales	unclassified_Helotiales	unclassified_Helotiales	unclassified	-0.276	-0.276	-0.276	0.828	0	0	0	1	0.83	0.029
**Arctic-S**	OTUe_1466	Fungi	Ascomycota	Leotiomycetes	Helotiales	unclassified_Helotiales	unclassified_Helotiales	unclassified	-0.366	-0.483	0.431	0.418	0	0	1	1	0.74	0.015
**Arctic-S**	OTUe_1552	Fungi	unclassified_Fungi	unclassified_Fungi	unclassified_Fungi	unclassified_Fungi	unclassified_Fungi	fungal_sp_6_GM_15_19	-0.169	-0.218	0.639	-0.252	0	0	1	0	0.64	0.019
**Arctic-S**	OTUe_1736	Fungi	Ascomycota	Eurotiomycetes	Chaetothyriales	Herpotrichiellaceae	Cladophialophora	Cladophialophora_chaetospira	-0.261	-0.484	0.525	0.220	0	0	1	1	0.65	0.042
**Arctic-S**	OTUe_1872	Fungi	Ascomycota	Lecanoromycetes	Lecideales	Porpidiaceae	Porpidia	Porpidia_flavocruenta	-0.238	-0.238	0.714	-0.238	0	0	1	0	0.71	0.029
**Arctic-S**	OTUe_1913	Fungi	Ascomycota	unclassified	unclassified	unclassified	unclassified	unclassified	-0.366	-0.512	0.589	0.290	0	0	1	1	0.76	0.009
**Arctic-S**	OTUe_2179	Fungi	Basidiomycota	Agaricomycetes	Agaricales	Clavariaceae	unclassified	unclassified	-0.716	0.370	0.207	0.139	0	1	1	1	0.72	0.014
**Arctic-S**	OTUe_2353	Fungi	Ascomycota	Eurotiomycetes	Eurotiales	Trichocomaceae	Penicillium	Penicillium_sp_NRRL_28156	-0.517	-0.231	0.419	0.328	0	0	1	1	0.65	0.046
**Arctic-S**	OTUe_2498	Fungi	Zygomycota	Zygomycota_class_Incertae_sedis	Mortierellales	Mortierellaceae	Mortierella	Mortierella_sp_4019_LB2012	-0.375	-0.237	-0.250	0.861	0	0	0	1	0.86	0.002
**Arctic-S**	OTUe_2690	Fungi	Ascomycota	Dothideomycetes	unclassified	unclassified	unclassified	unclassified	-0.240	-0.240	-0.130	0.609	0	0	0	1	0.61	0.029
**Arctic-S**	OTUe_2712	Fungi	Ascomycota	unclassified	unclassified	unclassified	unclassified	unclassified	-0.353	-0.203	-0.172	0.727	0	0	0	1	0.73	0.010
**Arctic-S**	OTUe_2735	Fungi	Ascomycota	Leotiomycetes	Helotiales	Helotiales_family_Incertae_sedis	Tetracladium	Tetracladium_sp_GPO_LL_01_C8	-0.353	-0.330	0.708	-0.024	0	0	1	0	0.71	0.013
**Arctic-S**	OTUe_2867	Fungi	Ascomycota	unclassified_Ascomycota	unclassified_Ascomycota	unclassified_Ascomycota	unclassified_Ascomycota	leotiomyceta_sp_GM_13_23	-0.413	-0.218	0.787	-0.156	0	0	1	0	0.79	0.007
**Arctic-S**	OTUe_2871	Fungi	Ascomycota	Eurotiomycetes	Chaetothyriales	Herpotrichiellaceae	unclassified	unclassified	-0.367	-0.278	0.694	-0.049	0	0	1	0	0.69	0.026
**Arctic-S**	OTUe_2974	Fungi	Ascomycota	Eurotiomycetes	Chaetothyriales	Herpotrichiellaceae	unclassified	unclassified	-0.164	-0.256	0.660	-0.240	0	0	1	0	0.66	0.007
**Arctic-S**	OTUe_3179	Fungi	Basidiomycota	Agaricomycetes	Sebacinales	Sebacinaceae	Sebacina	salal_root_associated_fungus_UBCTRA_154210	-0.443	-0.434	0.259	0.618	0	0	1	1	0.76	0.006
**Arctic-S**	OTUe_3298	Fungi	Ascomycota	Leotiomycetes	Helotiales	unclassified	unclassified	unclassified	-0.517	-0.243	0.466	0.294	0	0	1	1	0.66	0.037
**Alps-N**	OTUe_16	Fungi	Zygomycota	Zygomycota_class_Incertae_sedis	Mortierellales	Mortierellaceae	Mortierella	Mortierella_amoeboidea	-0.569	0.159	-0.171	0.581	0	1	0	1	0.64	0.031

**Alps-N**	OTUe_39	Fungi	Zygomycota	Zygomycota_class_Incertae_sedis	Mortierellales	Mortierellaceae	Mortierella	unclassified	-0.705	0.583	0.046	0.075	0	1	1	1	0.70 0.013
**Alps-N**	OTUe_48	Fungi	Ascomycota	Dothideomycetes	Capnodiales	Davidiellaceae	Cladosporium	unclassified	-0.626	0.047	0.649	-0.069	0	0	1	0	0.65 0.045
**Alps-N**	OTUe_66	Fungi	unclassified_Fungi	unclassified_Fungi	unclassified_Fungi	unclassified_Fungi	unclassified_Fungi	fungal_endophyte_sp_JP124	-0.748	0.568	0.010	0.171	0	1	1	1	0.75 0.010
**Alps-N**	OTUe_105	Fungi	Ascomycota	unclassified	unclassified	unclassified	unclassified	unclassified	-0.592	0.561	-0.253	0.284	0	1	0	1	0.73 0.013
**Alps-N**	OTUe_106	Fungi	Ascomycota	Lecanoromycetes	Acarosporales	Acarosporaceae	Acarospora	unclassified	-0.757	0.414	0.070	0.274	0	1	1	1	0.76 0.003
**Alps-N**	OTUe_206	Fungi	unclassified	unclassified	unclassified	unclassified	unclassified	unclassified	-0.659	0.568	-0.056	0.147	0	1	1	1	0.66 0.035
**Alps-N**	OTUe_242	Fungi	unclassified	unclassified	unclassified	unclassified	unclassified	unclassified	-0.526	0.365	-0.352	0.513	0	1	0	1	0.76 0.011
**Alps-N**	OTUe_272	Fungi	Ascomycota	unclassified	unclassified	unclassified	unclassified	unclassified	-0.517	0.485	-0.238	0.271	0	1	0	1	0.65 0.043
**Alps-N**	OTUe_302	Fungi	Zygomycota	Zygomycota_class_Incertae_sedis	Mortierellales	Mortierellaceae	Mortierella	Mortierella_sp_GW20_2	-0.641	0.477	0.272	-0.107	0	1	1	0	0.65 0.049
**Alps-N**	OTUe_307	Fungi	Ascomycota	unclassified	unclassified	unclassified	unclassified	unclassified	-0.315	-0.188	-0.148	0.651	0	0	0	1	0.65 0.038
**Alps-N**	OTUe_322	Fungi	Basidiomycota	unclassified_Basidiomycota	unclassified_Basidiomycota	unclassified_Basidiomycota	unclassified_Basidiomycota	Basidiomycota_sp_TP_Snow_Y1	-0.503	0.473	-0.286	0.316	0	1	0	1	0.68 0.031
**Alps-N**	OTUe_340	Fungi	Ascomycota	Dothideomycetes	unclassified	unclassified	unclassified	unclassified	-0.672	0.022	0.615	0.036	0	1	1	1	0.67 0.031
**Alps-N**	OTUe_610	Fungi	Basidiomycota	Tremellomycetes	Tremellales	Tremellales_family_Incertae_sedis	Dioszegia	Dioszegia_sp_TP_Snow_Y51	-0.209	-0.095	-0.369	0.673	0	0	0	1	0.67 0.039
**Alps-N**	OTUe_711	Fungi	unclassified_Fungi	unclassified_Fungi	unclassified_Fungi	unclassified_Fungi	unclassified_Fungi	fungal_sp_JH_68	-0.435	0.651	0.212	-0.429	0	1	1	0	0.75 0.006
**Alps-N**	OTUe_759	Fungi	Basidiomycota	Tremellomycetes	Tremellales	Tremellales_family_Incertae_sedis	Tremella	Tremella_sp_H_08013	-0.270	-0.270	-0.270	0.811	0	0	0	1	0.81 0.031
**Alps-N**	OTUe_768	Fungi	Ascomycota	Lecanoromycetes	Acarosporales	Acarosporaceae	Acarospora	unclassified	-0.660	0.424	0.144	0.091	0	1	1	1	0.66 0.042
**Alps-N**	OTUe_987	Fungi	Ascomycota	unclassified	unclassified	unclassified	unclassified	unclassified	-0.319	-0.124	-0.257	0.700	0	0	0	1	0.70 0.017
**Alps-N**	OTUe_1006	Fungi	Ascomycota	Dothideomycetes	Pleosporales	Phaeosphaeriaceae	Phaeosphaeria	Phaeosphaeria_sp_SS_F28	-0.224	-0.177	0.588	-0.187	0	0	1	0	0.59 0.034
**Alps-N**	OTUe_1162	Fungi	Ascomycota	Lecanoromycetes	Lecanorales	Lecanoraceae	Lecanora	unclassified	-0.202	-0.156	0.561	-0.202	0	0	1	0	0.56 0.024
**Alps-N**	OTUe_1568	Fungi	Ascomycota	unclassified	unclassified	unclassified	unclassified	unclassified	-0.145	-0.213	-0.213	0.571	0	0	0	1	0.57 0.029
**Alps-N**	OTUe_1783	Fungi	unclassified	unclassified	unclassified	unclassified	unclassified	unclassified	-0.452	0.598	-0.313	0.167	0	1	0	1	0.66 0.038
**Alps-N**	OTUe_1930	Fungi	Ascomycota	Lecanoromycetes	Lecideales	Lecideaceae	Lecidea	unclassified	-0.386	-0.269	0.012	0.644	0	0	0	1	0.64 0.048
**Alps-N**	OTUe_2000	Fungi	Ascomycota	Sordariomycetes	Hypocreales	unclassified_Hypocreales	unclassified_Hypocreales	Hypocreales_sp_Di59_5	-0.391	-0.391	0.221	0.561	0	0	1	1	0.68 0.031
**Alps-N**	OTUe_2011	Fungi	unclassified	unclassified	unclassified	unclassified	unclassified	unclassified	-0.505	0.459	-0.325	0.371	0	1	0	1	0.72 0.027
**Alps-N**	OTUe_2157	Fungi	Zygomycota	Zygomycota_class_Incertae_sedis	Mortierellales	Mortierellaceae	Mortierella	unclassified	-0.283	-0.283	0.724	-0.159	0	0	1	0	0.72 0.030
**Alps-N**	OTUe_2748	Fungi	Ascomycota	unclassified	unclassified	unclassified	unclassified	unclassified	-0.227	-0.227	-0.227	0.680	0	0	0	1	0.68 0.031
**Alps-N**	OTUe_3013	Fungi	Ascomycota	Leotiomycetes	Helotiales	Hyaloscyphaceae	Hyaloscypha	Hyaloscypha_sp_2_13c	-0.319	0.033	-0.371	0.657	0	0	0	1	0.66 0.019
**Alps-S**	OTUe_51	Fungi	Ascomycota	unclassified	unclassified	unclassified	unclassified	unclassified	-0.525	0.207	-0.361	0.679	0	1	0	1	0.77 0.007
**Alps-S**	OTUe_66	Fungi	unclassified_Fungi	unclassified_Fungi	unclassified_Fungi	unclassified_Fungi	unclassified_Fungi	fungal_endophyte_sp_JP124	-0.545	0.515	-0.266	0.297	0	1	0	1	0.70 0.018
**Alps-S**	OTUe_70	Fungi	Basidiomycota	Tremellomycetes	Cystofilobasidiales	Cystofilobasidiaceae	Mrakia	Mrakia_sp_ABS_2	-0.138	-0.314	0.675	-0.223	0	0	1	0	0.68 0.035
**Alps-S**	OTUe_71	Fungi	Zygomycota	Zygomycota_class_Incertae_sedis	Mortierellales	unclassified_Mortierellales	unclassified_Mortierellales	Mortierellales_sp_GD9A	-0.395	0.675	-0.409	0.128	0	1	0	1	0.70 0.022
**Alps-S**	OTUe_163	Fungi	Ascomycota	Leotiomycetes	Helotiales	unclassified	unclassified	unclassified	-0.273	-0.045	-0.339	0.656	0	0	0	1	0.66 0.044
**Alps-S**	OTUe_289	Fungi	Basidiomycota	Microbotryomycetes	Leucosporidiales	Leucosporidiaceae	Leucosporidium	Leucosporidium_drummii	-0.105	-0.295	0.823	-0.424	0	0	1	0	0.82 0.009
**Alps-S**	OTUe_340	Fungi	Ascomycota	Dothideomycetes	unclassified	unclassified	unclassified	unclassified	-0.439	0.563	0.201	-0.324	0	1	1	0	0.66 0.040
**Alps-S**	OTUe_420	Fungi	Ascomycota	Dothideomycetes	Pleosporales	Sporormiaceae	Preussia	Preussia_sp_AU_CryP01	-0.397	0.086	-0.346	0.657	0	0	0	1	0.66 0.044
**Alps-S**	OTUe_421	Fungi	Ascomycota	Sordariomycetes	unclassified_Sordariomycetes	unclassified_Sordariomycetes	unclassified_Sordariomycetes	Sordariomycetes_sp_genotype_165	-0.255	-0.082	-0.343	0.680	0	0	0	1	0.68 0.031
**Alps-S**	OTUe_431	Fungi	Ascomycota	Leotiomycetes	Helotiales	unclassified_Helotiales	unclassified_Helotiales	Helotiales_sp_3_MV_2011	-0.360	-0.047	-0.285	0.692	0	0	0	1	0.69 0.027
**Alps-S**	OTUe_1015	Fungi	unclassified_Fungi	unclassified_Fungi	unclassified_Fungi	unclassified_Fungi	unclassified_Fungi	unclassified	-0.282	0.643	0.111	-0.472	0	1	1	0	0.65 0.030
**Alps-S**	OTUe_1034	Fungi	Ascomycota	unclassified	unclassified	unclassified	unclassified	unclassified	-0.365	-0.190	-0.198	0.754	0	0	0	1	0.75 0.008
**Alps-S**	OTUe_1058	Fungi	Ascomycota	Dothideomycetes	Pleosporales	unclassified	unclassified	unclassified	-0.015	-0.223	-0.520	0.758	0	0	0	1	0.76 0.012
**Alps-S**	OTUe_1070	Fungi	unclassified	unclassified	unclassified	unclassified	unclassified	unclassified	-0.162	-0.136	0.554	-0.256	0	0	1	0	0.55 0.048
**Alps-S**	OTUe_1115	Fungi	Ascomycota	Dothideomycetes	Pleosporales	unclassified	unclassified	unclassified	-0.174	-0.255	-0.173	0.602	0	0	0	1	0.60 0.012
**Alps-S**	OTUe_1298	Fungi	unclassified	unclassified	unclassified	unclassified	unclassified	unclassified	-0.436	0.099	-0.482	0.818	0	0	0	1	0.82 0.005
**Alps-S**	OTUe_1341	Fungi	Ascomycota	unclassified	unclassified	unclassified	unclassified	unclassified	-0.173	-0.173	-0.173	0.518	0	0	0	1	0.52 0.028
**Alps-S**	OTUe_1537	Fungi	Ascomycota	Leotiomycetes	Helotiales	unclassified_Helotiales	unclassified_Helotiales	unclassified	-0.154	-0.217	-0.261	0.632	0	0	0	1	0.63 0.041
**Alps-S**	OTUe_2366	Fungi	Basidiomycota	Agaricomycetes	unclassified	unclassified	unclassified	unclassified	-0.266	-0.266	-0.266	0.797	0	0	0	1	0.80 0.028
**Alps-S**	OTUe_2615	Fungi	Ascomycota	Leotiomycetes	Leotiomycetes_order_Incertae_sedis	Leotiomycetes_family_Incertae_sedis	Geomyces	Geomyces_sp_AS3_1	-0.337	0.529	0.382	-0.574	0	1	1	0	0.79 0.001
**Alps-S**	OTUe_2633	Fungi	Ascomycota	Leotiomycetes	Leotiomycetes_order_Incertae_sedis	Leotiomycetes_family_Incertae_sedis	Geomyces	Geomyces_sp_AS3_1	-0.156	0.593	0.152	-0.588	0	1	1	0	0.65 0.048
**Alps-S**	OTUe_2979	Fungi	Ascomycota	Leotiomycetes	Helotiales	Helotiales_family_Incertae_sedis	Tetracladium	Tetracladium_sp_GPO_LL_01_C8	-0.141	-0.280	0.809	-0.388	0	0	1	0	0.81 0.005
**Alps-S**	OTUe_3335	Fungi	unclassified	unclassified	unclassified	unclassified	unclassified	unclassified	-0.478	-0.109	0.815	-0.228	0	0	1	0	0.82 0.009

1: significantly associated ($p < 0.05$), 0: non-significantly associated.
Arctic: Arctic soils, Alps: alpine soils, -N: north-exposed, -S: south-exposed
D-FTC: daily freeze-thaw cycles, W-FTC: weekly freeze-thaw cycles, ctrl +5°C: controls +5°C, ctrl -5°C: controls -5°C
